# HTLV-1 Integration into Transcriptionally Active Genomic Regions Is Associated with Proviral Expression and with HAM/TSP

**DOI:** 10.1371/journal.ppat.1000027

**Published:** 2008-03-21

**Authors:** Kiran N. Meekings, Jeremy Leipzig, Frederic D. Bushman, Graham P. Taylor, Charles R. M. Bangham

**Affiliations:** 1 Department of Immunology, Wright-Fleming Institute, Imperial College London, London, United Kingdom; 2 Department of Microbiology, University of Pennsylvania School of Medicine, Philadelphia, Pennsylvania, United States of America; 3 Department of Genitourinary Medicine and Communicable Diseases, Wright-Fleming Institute, Imperial College London, London, United Kingdom; King's College London, United Kingdom

## Abstract

Human T-lymphotropic virus type 1 (HTLV-1) causes leukaemia or chronic inflammatory disease in ∼5% of infected hosts. The level of proviral expression of HTLV-1 differs significantly among infected people, even at the same proviral load (proportion of infected mononuclear cells in the circulation). A high level of expression of the HTLV-1 provirus is associated with a high proviral load and a high risk of the inflammatory disease of the central nervous system known as HTLV-1-associated myelopathy/tropical spastic paraparesis (HAM/TSP). But the factors that control the rate of HTLV-1 proviral expression remain unknown. Here we show that proviral integration sites of HTLV-1 *in vivo* are not randomly distributed within the human genome but are associated with transcriptionally active regions. Comparison of proviral integration sites between individuals with high and low levels of proviral expression, and between provirus-expressing and provirus non-expressing cells from within an individual, demonstrated that frequent integration into transcription units was associated with an increased rate of proviral expression. An increased frequency of integration sites in transcription units in individuals with high proviral expression was also associated with the inflammatory disease HAM/TSP. By comparing the distribution of integration sites in human lymphocytes infected in short-term cell culture with those from persistent infection *in vivo*, we infer the action of two selective forces that shape the distribution of integration sites *in vivo*: positive selection for cells containing proviral integration sites in transcriptionally active regions of the genome, and negative selection against cells with proviral integration sites within transcription units.

## Author Summary

The human leukaemia virus HTLV-1 causes a lifelong infection that cannot be cleared by the immune system. By integrating into the host's DNA, the virus can lie dormant within the cell. The virus can then be reactivated, by processes that are only partly understood, causing the infected cell to multiply and leading to an increase in the quantity of virus in the infected person. In some infected people, the virus is reactivated much faster than in others, and such people are more likely to develop HTLV-1-associated inflammatory diseases such as HAM/TSP, which results in paralysis of the legs. It is not understood what determines this rate of viral reactivation in each person. In this study, we found that integration of HTLV-1 in the host's DNA close to other genes was associated with faster viral reactivation and a higher probability of HAM/TSP. By comparing the viral integration site positions in samples from patients and in cells infected with HTLV-1 in the laboratory, we can identify some of the major forces that allow the virus to persist lifelong whilst avoiding eradication by the immune response.

## Introduction

The completion of the human genome sequence has made genome-wide studies of retroviral integration possible. Such studies have led to a good understanding of the targeting preferences of retroviruses for integration. However, the genomic distribution of integration sites has not been widely studied in the context of persistent infection, where infected cells are subjected to additional selection forces such as the immune response. Here we tested the hypothesis that *in vivo* selection influences the genomic distribution of proviral integration sites in persistent HTLV-1 infection.

Genome-wide studies have revealed that proviral integration is not random, but that each retrovirus has distinct target site preferences [1]–[4]. For instance, HIV shows a bias towards integration into genes, whereas MLV integration is biased towards CpG islands and the transcriptional start sites of genes [1]–[4]. Each retrovirus also targets a characteristic inverted repeat consensus nucleotide sequence at the site of integration [5]–[11]. The different targeting preferences of the individual retroviruses are determined by several known factors, including the properties of the viral integrase [12], DNA binding proteins [13],[14], cellular targeting proteins [15]–[19] and the chromatin structure at the point of integration [20],[21].

A recent study of HTLV-1 integration sites isolated from HeLa cells infected *in vitro* showed that HTLV-1 has integration site preferences most similar to those of avian sarcoma-leukosis virus (ASLV) [22]. Both ASLV and HTLV-1 were found to target genes, transcriptional start sites and CpG islands. Although in each case the bias was statistically significant, the magnitude of the effect was lower than observed in integration with other retroviruses (HIV-1, SIV, MLV and FV) [22]. Since the Integrases of ASLV and HTLV-1 are more closely related in sequence to each other than to the other retroviruses, this observation supported the idea that Integrase is a major determinant of retroviral integration site targeting.

HTLV-1 is associated with the neoplastic disease Adult T cell Leukemia (ATL) and the inflammatory condition HTLV-1-associated myelopathy/ tropical spastic paraparesis (HAM/TSP). The mechanisms of pathogenesis of HAM/TSP have not been elucidated. However, it is well established that the proviral load of HTLV-1–the proportion of peripheral blood mononuclear cells (PBMCs) that contain a provirus–varies widely among HTLV-1-infected individuals and is strongly correlated with the risk of HAM/TSP [23]. In addition, there is evidence from the study of the cell-mediated immune response that the rate of HTLV-1 proviral expression at a given proviral load correlates with the outcome of infection [24]. An individual patient's proviral load remains constant over time [25]. In contrast, proviral loads show large variation between patients [23]. The causes of this proviral load variation are not yet clear. It was recently demonstrated that variation between individuals in the efficiency of the cytotoxic T lymphocyte (CTL) response determined approximately 30% of the observed between-individual variation in proviral load [26]. An additional 13% of proviral load was determined by the rate of provirus expression, independently of CTL efficiency [24]. We hypothesised that the CTL-independent variation in proviral load was due to molecular factors that affect proviral load and provirus expression. We postulated that one such factor was the integration site of the virus.

The proviral load of HTLV-1 appears to be determined by a dynamic balance between viral replication and the host immune response [27]. HTLV-1 has two potential routes of infection: infectious transmission (across the virological synapse [28]) or mitotic replication of an infected cell (instigated by the viral transactivating protein Tax [29],[30]). The sequence of HTLV-1 is stable within an individual, indicating that the proviral load *in vivo* is maintained chiefly by proliferation of infected cells [31]. This interpretation is supported by the observation of large clones of infected cells with a common integration site *in vivo* [29],[30]. We have hypothesised that infectious transmission of HTLV-1 is important early in infection whilst mitotic replication may be responsible for maintaining proviral load later in infection once persistent infection has been established and reached equilibrium with the immune response [27].

There are few existing data on the distribution of HTLV-1 integration sites in non-malignant cases of infection *in vivo.* Previous studies suggested that genes are favoured targets for the integration of HTLV-1 in ATL [32]–[34] whereas integration sites isolated from asymptomatic carriers (ACs) and from patients with HAM/TSP showed no preference for transcriptionally active regions [32],[35]. The power of these studies was, however, limited by the small number of integration sites and by investigation of few genomic parameters. In this study we compared the genomic characteristics of HTLV-1 integration sites resulting from infection in a cell culture system *in vitro* with those of integration sites from freshly isolated PBMCs, in which the integration sites have been subject to years of *in vivo* selection. The results suggest positive selection for cells that possess a provirus integrated in a transcriptionally active genomic region and negative selection against cells with a provirus integrated in a transcriptional unit (i.e. a gene) during persistent infection *in vivo*. Moreover, frequent integration in transcriptionally active genomic regions was associated with a high level of HTLV-1 proviral expression and with the inflammatory disease HAM/TSP.

## Results

The datasets used in this analysis are summarised in Table 1 and can be downloaded from http://bushmanlab.pbwiki.com/f/Meekings.fa. Using linker-mediated PCR (LM-PCR) we cloned the genomic integration sites in human lymphocytes (Jurkat) resulting from co-incubation with a chronically HTLV-1-infected cell line, MT-2. For each observed integration site, ten matched random control sites (MRC) were generated. Each MRC lay the same distance from a randomly chosen NlaIII site in the genome as the observed integration site from its respective NlaIII site. This procedure controlled for any potential bias resulting from a non-random distribution of the restriction enzyme cleavage sites in the genome [36]. The experimental HTLV-1 integration sites were then compared to the MRCs using a logistic regression model and ?-squared analysis. A detailed description of the statistical analysis is presented in Protocol S1. These sites were compared to an existing set of 527 integration sites generated by the *in vitro* infection of HeLa cells by HTLV-1 [22] and with 5270 corresponding MRCs. Our stringent selection criteria (see Materials and Methods) enabled us to map the genomic sites of 527 of the 541 sites reported [22]. The comparison of these two datasets from *in vitro* infection, using a logistic regression model, showed no significant differences in any of the genomic parameters analysed. We therefore combined the two datasets in subsequent analysis.

**Table 1:** Datasets used in this study

Author	Reference	Source of integrations	Number of sites	Use of Sites
Derse et al.	22	*In vitro* infection of HeLa cells with virus like particles	527	Comparison of in vitro and in vivo integration sites (Fig 1).
Meekings et al.	This Study	*In vitro* infection of Jurkat cells by co-culture with infected cell line	266	Comparison of in vitro and in vivo integration sites (Fig 1).
Meekings et al.	This Study	*In vivo* infected PBMCs isolated from naturally infected HAM/TSP patients and Acs blood samples	313	Comparison of *in vitro* and *in vivo* integration sites (Fig 1). Investigation of the association of the distribution of integration sites and Tax expression between individuals (Fig 3).
Meekings et al.	This Study	Flow cytometric sorting of provirus expressing vs. non-expressing naturally infected patient PBMCs	40	Investigation of the association of the distribution of integration sites and Tax expression within an individual (Fig 4)

We then isolated and analysed 313 integration sites from 24 individuals infected with HTLV-1. The individuals comprised 11 ACs and 13 patients with HAM/TSP with proviral loads ranging from 0.49 to 36.3 proviral copies/ 100 PBMCs. Details on individuals used in this study and the number of integration sites contributed by each individual are given in Table S1. Many integration sites were detected more than once in the same individual, reflecting the T cell proliferation that maintains the HTLV-1 proviral load (see Introduction). In the ensuing analysis, each distinct integration site was counted once. Analysis of the relationship between integration site distribution and the relative clonal abundance of HTLV-1 proviruses will require a high-throughput sequencing study: such a study is now underway.

In this paper we use the term ‘*in vitro* integration*’* to refer to proviral integration generated by infection in cell culture , and ‘*in vivo* integration*’* to refer to integration sites isolated from individuals persistently infected with the virus.

### HTLV-1 integration *in vivo* and *in vitro* is identical at the nucleotide level

Studies of other retroviruses have shown that integration occurs in a statistically-defined consensus sequence, identified by analysis of a large number of integration sites [5]–[11],[22]. It has been suggested that this consensus nucleotide sequence preference is an inherent property of the retrovirus [5]. Therefore, it should not be influenced by *in vivo* selection. In agreement with this, both *in vivo* and *in vitro* integration sites showed an identical consensus sequence at the point of integration (Figure S1).

### Distribution of HTLV-1 proviral integration sites *in vitro* and *in vivo*

**Figure 1:**
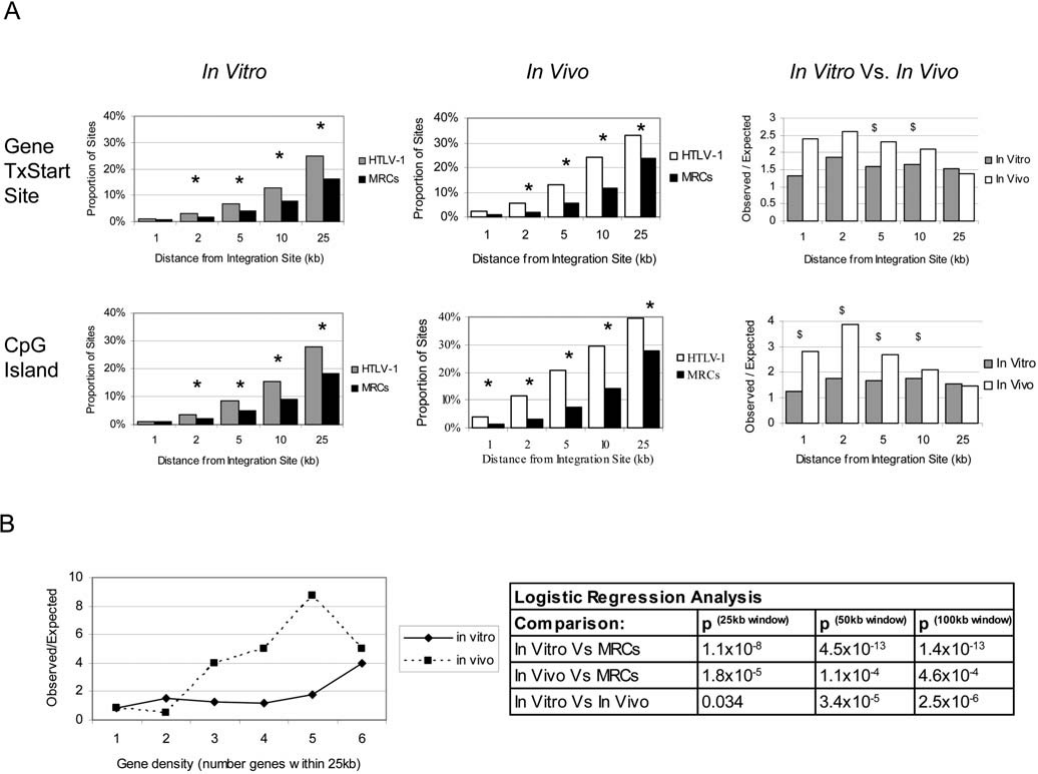
Integration of HTLV-1 in transcriptionally active genomic regions. Panel A: Near gene transcriptional start sites and CpG islands. HTLV-1 showed significantly greater than expected frequency of integration in the vicinity of RefSeq transcriptional start sites (TxStart sites) and CpG islands in cell culture *in vitro* and in persistent infection *in vivo*. The vertical axes for the *in vitro* and *in vivo* datasets show the proportion of observed HTLV-1 integration sites or expected MRC sites in the vicinity of the respective genomic feature. * indicates a significant difference between HTLV-1 sites and MRCs, by ?-squared analysis (p<0.05). There was a significantly greater association with integration in the proximity of TxStart sites and CpG islands comparing the distribution of observed HTLV-1 integration sites to expected MRC sites *in vivo* and *in vitro* (vertical axis, *in vitro* vs *in vivo*). ^$^ indicates a significant difference between the frequency of HTLV-1 *in vivo* sites and *in vitro* sites, by logistic regression (p<0.005). Panel B: In regions of high gene density. HTLV-1 showed an increased frequency of integration in gene dense regions. Gene density in regions from 25 kb to 1 Mb around the integration site was analysed. In all region sizes, there was a greater association of HTLV-1 integration in gene dense regions both *in vivo* and *in vitro.* However, there was a significantly greater association between proviral integration frequency and gene density in persistent infection *in vivo* than was seen *in vitro.* A graphical illustration of the 25 kb region and data on the logistic regression results comparing the *in vivo* and *in vitro* HTLV-1 datasets for the first three region sizes is given (Panel B).

The datasets of HTLV-1 integration sites were analysed for proximity to genomic features of transcriptional activity including transcription units, transcriptional start sites and CpG islands [3]. Here ‘transcriptional unit’ denotes a full-length unprocessed RNA transcript. The observed frequency of HTLV-1 integration near each feature was compared to the expected frequency derived from analysis of the MRC sites using statistical analyses (?-squared and logistic regression). A recent study of the integration sites of HTLV-1 *in vitro* reported significant targeting of transcription units and a weak but significant targeting of regions containing transcriptional start sites and CpG islands [22]. Our *in vitro* co-culture system revealed identical integration preferences. We therefore combined both sets of HTLV-1 *in vitro* integration sites and compared the combined dataset to the MRC sites for proximity to genomic features of transcriptional activity. Analysis of the proportions of HTLV-1 integration sites and MRC sites within a specified distance (window size between 1 kb and 25 kb) of a CpG island showed a significantly higher frequency of HTLV-1 integration sites than MRC sites at all distances above 2 kb (3.5% of HTLV-1 integration sites lying ±2 kb of a CpG island compared with 2.0% of MRC sites; observed/expected (O/E) ratio of 1.75; p = 0.0059, ?-squared, Figure 1A). *In vitro* there was no significant difference in the frequency of HTLV-1 proviral integration sites within CpG islands themselves, and ±1 kb of a CpG island, compared to MRCs, but statistical power was limited by the small numbers of sites in this very small genomic region (0.13% vs 0.24% within a CpG island, Table 2; 1.13% and 0.91% ±1 kb, Figure 1).

**Table 2:** Percentage of integration sites residing within gene coding regions/CpG islands *in vitro* and *in vivo*

Genomic Feature:	*In Vitro*: Cell Culture		*In Vivo*: Persistent Infection	
	(N = 793):		(N = 313):	
	Sites	MRCs	Sites	MRCs
CpG island$	0.13	0.24	1.92*	0.61
Acembly gene$	64.6*	52.0	51.4	52.2
GenScan gene	70.7	69.5	69.0	71.4
Known gene	47.9*	38.1	40.3	39.5
RefSeq gene¥	44.5*	34.0	36.1	35.2
Unigene gene	48.8*	40.8	41.2	41.4
Ensembl gene$	49.3*	37.5	38.7	38.9

The proportion of HTLV-1 integration sites lying within a CpG island or the coding region of a gene (as defined by specified databases) in cell culture *in vitro* and in persistent infection *in vivo* were compared to the proportion of Matched Random Control (MRC) sites derived *in silico* (see Materials and Methods).. Human gene annotation tables from the UCSC genome database include Acembly genes, GenScan genes, Known genes, RefSeq genes, Unigene and Ensembl genes. * indicates a significant difference between the HTLV-1 proviral dataset and MRCs (p<0.01). ^$ ^and ^¥^ indicate a significant difference between *in vivo* and *in vitro* HTLV-1 proviral datasets (p<0.01 and p<0.05 respectively).

Compared to the MRC frequencies, there were also significantly more HTLV-1 proviral integration sites in the vicinity of gene transcriptional start sites. This was evident at all distances investigated (2–25 kb from a transcriptional start site) (i.e. 3.2% of HTLV-1 integration sites lay ±2 kb of a RefSeq gene transcriptional start site compared with 1.7% of MRCs; O/E = 1.88; p = 0.026, ?-squared, Figure 1A). There was also a statistically significant excess of proviral integration sites present in transcription units compared to MRC frequencies using a number of different gene annotation databases (e.g. 44.5% in RefSeq genes compared with 34.0% of MRCs; O/E = 1.31; p = 3.3×10^−9^, ?-squared, Table 2). Further, by investigating the gene density in genomic regions containing an HTLV-1 provirus, we observed that the proviral integration sites were associated with regions of high gene density: there was a significantly increased frequency of HTLV-1 provirus integration compared to MRC sites in regions of higher gene density at all region sizes investigated (from 25 kb to 8 Mb from the integration site; p values ranged from 3.1×10^−19 ^to 1.1×10^−8^; logistic regression analysis, Figure 1B).

The integration sites identified in persistent infection *in vivo* were also associated with transcriptionally active regions as shown by their proximity to CpG islands, transcriptional start sites and their positioning in gene-dense regions. There were three times as many HTLV-1 proviral integrations (1.92%) as MRC sites (0.61%) lying within a CpG island (Table 2, p = 0.007, ?-squared analysis). In addition, there was a statistically significant increased frequency of HTLV-1 integration compared to MRC sites near CpG islands at all distances investigated (1 kb to 25 kb from a CpG island) (Figure 1A). For instance, compared to the 3.0% of MRC sites, 11.5% of the HTLV-1 proviral integration sites from persistent infection lay ±2 kb of a CpG island (O/E = 3.83; p = 4.1×10^−14^, ?-squared). There was also a significant excess frequency of HTLV-1 proviral integration sites near gene transcriptional start sites: 5.4% of HTLV-1 sites lay within ±2 kb of a RefSeq gene transcriptional start site compared with 2.1% of MRCs; O/E  = 2.57; p = 0.0002, ?-squared (Figure 1A). The integration sites identified in persistent infection were also found in regions of higher gene density than MRC sites (Figure 1B). However, in contrast to the *in vitro* integration sites, the frequency of integration in transcription units *in vivo* was not significantly different from expectation (the expected value was calculated, as before, from the distribution of MRCs) (Table 2). Thus, although the integration sites isolated from persistent infection were shown to be associated with transcriptionally active genomic regions, this did not include an association with transcriptional units.

The methods used in this study and by Derse et al used different restriction enzymes (NlaIII and MseI respectively). The proportion of MRCs that lie within a given distance from a CpG island differed according to the restriction enzyme used: 3.0% of the MRC *in vivo* sites generated with NlaIII lay ±2 kb of a CpG island, compared with 2.0% of the MRC *in vitro* sites (of which 266 were generated with NlaIII and 527 generated with MseI). Also, 2.1% of NlaIII *in vivo* sites lay within ±2 kb of a transcriptional start site compared to 1.7% of the *in vitro* MseI/NlaIII sites. That is, the respective restriction enzyme sites are differently distributed in the genome with respect to the features associated with transcriptional activity, and it was therefore essential to use different MRC sets in each case. We therefore compared each dataset to its respective MRC set before comparing the experimental datasets to each other.

### *In vivo* selection shapes integration site distribution

**Figure 2:**
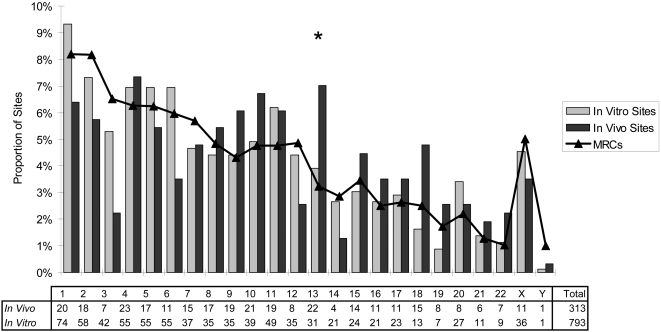
Chromosomal distribution of HTLV-1 integration sites *in vitro* and *in vivo.* The distribution of HTLV-1 integration sites within the genome *in vivo* during persistent infection was non-random across all chromosomes compared to matched random controls (logistic regression analysis, p = 0.00095). In particular, chromosome 13 had a significantly increased frequency of integration sites compared to MRC sites (?-squared test; p = 0.014 after correction for multiple comparisons). The vertical axis indicates the proportion of integration sites lying in each chromosome. Integration of HTLV-1 *in vitro* (combined data from the co-culture sites obtained in this report and sites generated in a previous report [22]) showed a weak overall chromosome bias (logistic regression analysis, p = 0.016) but no particular chromosome was favoured. Logistic regression analysis confirmed that the chromosomal distribution differed significantly *in vivo* compared to *in vitro.* * indicates a significant difference between HTLV-1 dataset and MRCs (logistic regression analysis, p = 0.014).

To test for evidence of *in vivo* selection on the distribution of integration sites, we compared the genomic distribution of 313 *in vivo* integration sites with the combined set of 793 *in vitro* integration sites using a logistic regression model. First, the logistic regression model was used to test for non-random frequency of integration across all chromosomes *in vitro* and *in vivo*. The chromosomal distribution of HTLV-1 integration sites *in vivo* differed significantly from the MRCs (Figure 2; p = 0.00095). Using ?-squared analysis and correcting for multiple comparisons, there was a significant excess frequency of integration in Chromosome 13 *in vivo* (p = 0.014). The logistic regression model also suggested non-random chromosome distribution of HTLV-1 integration *in vitro* (Figure 2, p = 0.016, ?-squared analysis) but this bias was not statistically significant after correcting for multiple comparisons (p>0.1). However, logistic regression confirmed a significant difference (p = 0.006) between the chromosomal distribution of sites *in vivo* and *in vitro.*

Compared with the *in vitro* integration sites, there were significantly more integrations *in vivo* into transcriptionally active genomic regions, i.e. near a CpG island or a transcriptional start site. The ratio of the numbers of observed HTLV-1 integration sites to expected (MRC) sites lying within a specified distance (ranging from 1 to 25 kb) from either a RefSeq gene transcriptional start site or a CpG island were compared *in vitro* and *in vivo*. Although both datasets showed a greater than expected frequency of proviral integration in transcriptionally active regions, there were also significantly more integrations near both CpG islands and transcriptional start sites *in vivo* than *in vitro* (Figure 1A; p<0.005, logistic regression analysis). There were also significantly more integrations lying within a CpG island (p = 9×10^−4^, logistic regression analysis) (Table 2) *in vivo* than *in vitro*. In addition, we analysed the gene density in a range of window sites (±25 kb to ±1 Mb) around each integration site. Logistic regression analysis showed significantly more integrations into regions of higher gene density *in vivo* than *in vitro* at all window sizes tested (p = 0.032 at ±25 kb; p = 2.5×10^−6^ at ±100 kb; Figure 1B). However, although the observed association with integration into transcriptionally active regions was stronger *in vivo* than *in vitro,* there were significantly fewer integrations into transcription units *in vivo* (Acembly, RefSeq and Ensemble gene definitions tested; p = 2.5×10^−4^, 0.044, 0.0065 respectively) (Table 2). Whereas HTLV-1 proviral integration frequency in transcription units exceeded expectation *in vitro, in vivo* there was no deviation from expected frequencies.

### The distribution of integration sites is associated with proviral expression and disease status

We wished to compare the distribution of integration sites between individuals with a high proviral load and those with a low load; between those with high levels of expression of the viral protein Tax and those with low levels; and between patients with HAM/TSP and ACs. In each comparison, the individuals in Table S1 were split into 2 equal groups based on the above parameters (i.e. above and below the median value of the respective parameters) and the integration sites from the two respective groups of subjects were compared using a logistic regression model as set out in Protocol S1. There were no significant differences in the genomic characteristics of the integration sites between individuals with a high proviral load and those with a low proviral load (data not shown). However, differences in the distribution of integration sites were apparent according to the level of expression of Tax and the disease status. The HAM/TSP group and high Tax group each showed a consistent tendency to a higher frequency of integrations both in the vicinity of genes and in regions of higher gene density. Of the integration sites isolated from high Tax-expressing individuals, 38% were located in RefSeq genes, compared with only 33% of sites from low Tax-expressing individuals. The high Tax-expressing group also had a significantly higher gene density in the region surrounding the integration site than the low Tax-expressing group (p =  0.034; logistic regression model using a 500 kb window around each integration site, data not shown).

**Figure 3:**
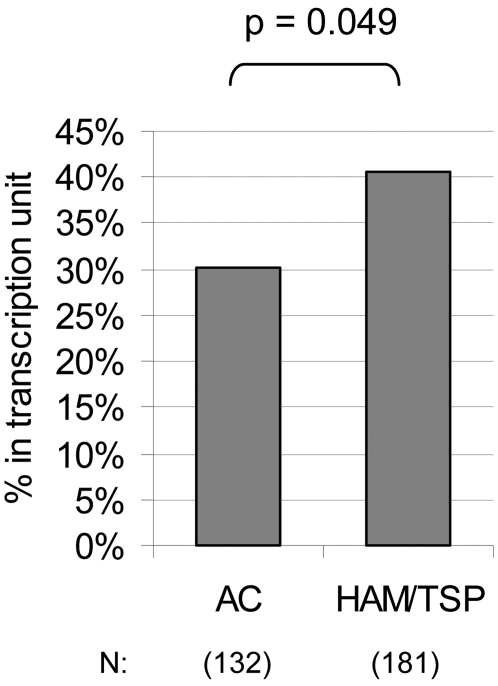
The genomic distribution of HTLV-1 integration sites was associated with disease status. Integration sites isolated from the PBMCs of individuals with HAM/TSP were compared to integration sites from ACs using logistic regression analysis. The integration sites from the HAM/TSP group were associated with RefSeq genes (logistic regression model, p = 0.049). The numbers of integration sites compared in each group are indicated.

There is evidence that the Tax protein is expressed at a significantly higher frequency in patients with HAM/TSP than in ACs with a similar proviral load [24]. Since the data presented above showed that the proviral integration site distribution was associated with the level of Tax protein expression, we wished to test the hypothesis that the integration site distribution differed between HAM/TSP patients and ACs. The results show that the HAM/TSP patients had significantly more integration sites located in RefSeq genes (41%) compared to the ACs (30%) (Figure 3, p = 0.049; logistic regression model).

**Figure 4:**
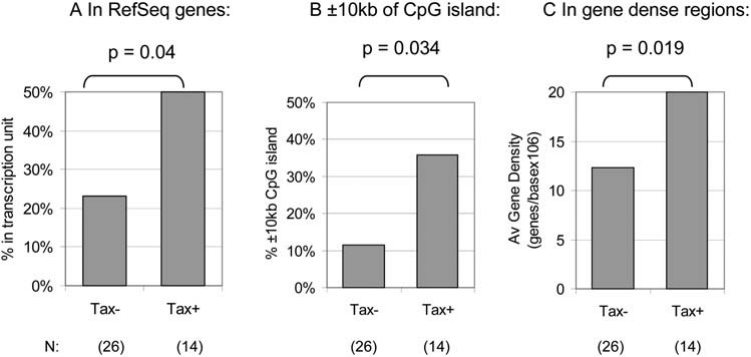
The genomic distribution of HTLV-1 integration sites within individuals was associated with provirus expression. CD8^+^ cell-depleted PBMCs from an HTLV-1-infected individual were separated into Tax^+^ and Tax^−^ fractions after 18 hrs incubation *in vitro*. Integration sites were cloned from each fraction. The integration sites from three independent experiments were combined and the distribution of sites between the Tax^+^ and Tax^−^ fractions compared using logistic regression analysis. The Tax^+^ fraction had significantly more integrations in RefSeq genes than did the Tax^−^ fraction (Panel A, p = 0.04). The Tax^+^ fraction also had a higher proportion of integrations lying within 10 kb of a CpG island (Panel B, p = 0.034) and a higher gene density around the integration site (Panel C, p = 0.019 using 1 Mb around each integration site). The numbers of integration sites compared in each group are indicated.

These observations suggested that the integration site is associated with the rate of expression of the provirus *in vivo*, i.e. that integration in gene-dense regions is concomitant with higher provirus expression and consequently with a higher risk of the disease HAM/TSP. We tested the validity of this conclusion in an independent series of experiments by comparing the genomic distribution of proviral integration sites between HTLV-1-infected T cells that expressed Tax spontaneously within 18 hrs and those from the same individual that did not express Tax. In independent experiments, CD8^+^ cell-depleted PBMCs from each of three patients with HAM/TSP were incubated for 18 hrs *in vitro* to allow spontaneous Tax expression. Each of the three individuals had approximately 5% Tax^+^CD4^+^ T-cells after culture. After intracellular staining with a fluorescent-labelled monoclonal antibody for the Tax protein, the Tax-expressing cells were separated from Tax non-expressing cells by flow cytometric sorting. The integration sites in each respective cell population were then cloned. The integration sites from the Tax-expressing (Tax^+^) fraction for the three individuals were pooled and the distribution compared to that of the Tax non-expressing fraction (Tax^−^). The results (Figure 4) corroborated the hypothesis that the distribution of integration sites *in vivo* is associated with the rate of proviral (Tax) expression. The Tax^+^ fraction had a significantly higher proportion of HTLV-1 integration sites in genes compared to the Tax^−^ fraction (50% and 23% in RefSeq genes respectively) (Figure 4A). This difference was significant at the one-tailed level (p = 0.04). The Tax^+ ^cell fraction also had a significantly higher proportion of HTLV-1 integration sites in the vicinity of a CpG island ( with 35.7% of integration sites lying within ±10 kb from a CpG island in Tax^+^ cells compared with 11.5% of integration sites in Tax^− ^cells, p = 0.034, ?-squared analysis, Figure 4B). Finally, the Tax^+^ fraction had a significantly higher gene density around each integration site than the Tax^−^ fraction (p = 0.019; logistic regression model using a 1 Mb window around each integration site, Figure 4C). In summary the results show that, both between and within patients, proviral integration into areas of transcriptional activity was associated with Tax expression.

## Discussion

The results presented here show that the integrated HTLV-1 provirus is associated with transcriptionally active regions of the human genome both *in vitro* in cell culture and *in vivo* in persistent infection. This was shown by an increased frequency of integration in gene-dense regions, near CpG islands and near transcriptional start sites compared to controls. However, we also found significant differences in the distribution of HTLV-1 integration sites when comparing the *in vitro* sites with those identified in patients' PBMCs, which had been subjected to years of immune and viral selection *in vivo*. We used 2 datasets of HTLV-1 sites generated *in vitro*: an existing set resulting from infection of HeLa cells with virus-like particles [22] and a novel set formed by the infection of Jurkat cells by short-term co-culture with MT2 cells. The presented results are in agreement with the analysis by Derse et al on the set of HeLa-derived HTLV-1 proviral integration sites [22]. In addition, the distribution of integration sites did not differ between these two *in vitro* datasets in any genomic parameter tested. This finding is consistent with previous evidence that the distribution of retroviral integration sites is largely independent of the cell type [1], [4], [6], [22], [36]–[39]. We then pooled these sites to compare the genomic distribution of 793 *in vitro* sites with 313 sites resulting from persistent infection *in vivo*.

Two major differences were found between the distribution of *in vivo* and *in vitro* HTLV-1 integration sites. First, there was a significantly stronger association with integration in transcriptionally active regions *in vivo* than *in vitro*. That is, there were significantly more integrations *in vivo* into regions of high gene density, near RefSeq gene transcriptional start sites, and near CpG islands. Second, there were significantly fewer integrations into transcription units *in vivo* than *in vitro*: whereas HTLV-1 showed a significant bias towards integration in transcription units *in vitro* both in HeLa and in Jurkat cells, there was no deviation from expected frequencies *in vivo*. These results are consistent with the existence of two selection forces that act *in vivo* to shape the initial distribution of integration sites: positive selection for cells containing an integration within a transcriptionally active regions of the genome, and negative selection against gene disruption resulting from proviral integration within a transcription unit.

Positive selection for proviruses integrated into transcriptionally active genomic regions is likely to result from the actions of the HTLV-1 Tax protein, which transactivates transcription of both viral and host genes and causes clonal T cell proliferation. This conclusion is consistent with two existing lines of evidence on the mechanism of HTLV-1 persistence *in vivo.* First, there is evidence that HTLV-1 persists *in vivo* during the chronic phase of infection chiefly by clonal proliferation of infected cells [29],[40]. Second, there is an abnormally fast turnover rate of HTLV-1-infected T cells *in vivo*, especially of Tax-expressing cells [41],[42], indicating that the selective proliferation of HTLV-1-infected T cell clones *in vivo* depends on persistent expression of the provirus. It is possible that proviral integration in transcriptionally active regions also affects the expression of host genes lying in the vicinity of the integration site, so conferring a growth advantage on the infected clone.

We observed a significantly lower frequency of HTLV-1 proviral integration in transcription units *in vivo* than *in vitro.* We postulate that the lower frequency *in vivo* results from the disruption of gene function. If correct, this conclusion implies the existence of haploinsufficiency in many human genes [43]. There are currently insufficient published data available to make possible an accurate estimate of the proportion of human genes that are subject to haploinsufficiency. However, there is an increasing number of reports of human diseases, including cancer and developmental disorders, that are associated with haploinsufficiency [44]–[46]. Haploinsufficiency has also shown to be a common phenomenon in yeast [47],[48]. Negative selection against integration into transcription units was not observed in HIV infection, in which integration was biased towards transcription units both *in vitro* and *in vivo* [36],[49]. Most cells productively infected by HIV have a short lifespan, perhaps too short to allow negative selection against integration in transcription units [50]. In contrast to HIV, HTLV-1 infection results in increased lymphocyte turnover via clonal proliferation [41]. We suggest that this T cell proliferation maintains the proviral load and the lymphocyte count in the face of negative selection against integrations in genes.

We analysed the distribution of *in vivo* proviral integration sites to test the hypothesis that the distribution of integration sites is associated with proviral load, provirus expression and disease status. There were no statistically significant differences in the distribution of integration sites between the high-load and low-load subjects. However, there were significant differences in the distribution of integration sites between patients with high expression of the viral protein Tax and those with low expression, and between patients with HAM/TSP and ACs. Both the high Tax and the HAM/TSP group showed a greater frequency of proviral integrations in genes compared to the low Tax and AC group respectively. We conclude that integration in regions of higher gene density was concomitant with higher levels of the viral protein Tax. Since the level of Tax expression correlates with disease risk [24], this integration distribution also manifests as a higher proportion of integrations in gene-dense regions in HAM/TSP patients compared to ACs. We propose that integration in transcriptionally active genomic regions favours HTLV-1 proviral expression and persistence, but that this is counter-selected by an efficient immune (particularly CTL) response. This conclusion is consistent with evidence that the efficiency of the CTL response correlates negatively with proviral load [26] and with recent evidence that HTLV-1 proviral expression contributes to the persistence of HTLV-1 *in vivo* [41].

As a more stringent test of the relationship between the integration site and the level of provirus expression, integration sites from Tax^+^ and Tax^−^ provirus-positive cells isolated from cultured PBMCs were amplified by LM-PCR. We analysed these sites to test the hypothesis that the Tax^+^ cell fraction had significantly more integrations in transcriptionally active regions. The results showed that the Tax^+ ^fraction indeed had significantly more integration sites lying in transcription units: 50% of the sites in the Tax^+^ fraction were located in RefSeq transcription units compared to only 23% in the Tax^−^ fraction. Similar experiments with other retroviruses have also shown an association between integration in gene-rich regions and viral transcription *in vitro* [12],[51]. The results of this within-patient experiment (Figure 4) corroborated the relationship between integration site distribution and Tax expression. The results also revealed a stronger association between integration in genes and Tax expression within individuals than that observed in the between-individual experiment. We suggest that this stronger association results from the greater difference in Tax expression between Tax^+^ and Tax^−^ cells (an all-or-nothing effect) than between the high-Tax and low-Tax patient groups, which represent two halves of a continuous distribution. In addition, other factors such as variation in the host immune response to HTLV-1 are likely to contribute to between-individual variation in the intensity of selection for proviral expression [26].

We propose that an individual's steady-state rate of proviral expression (measured as Tax protein), and the accompanying risk of inflammatory diseases such as HAM/TSP, are the result of an equilibrium between HTLV-1 replication and the immune response *in vivo* (Figure S2). Selection for cells with high levels of Tax expression leads to an increased frequency of proviruses integrated in gene-dense regions of the genome. Clonal proliferation of an infected cell leads to dominance of Tax-expressing clones. The increased proviral load resulting from this proliferation will increase the rate of infectious spread of the virus and thereby increase the frequency of integration in genes, owing to the intrinsic preference of the virus. Balancing these positive forces, the specific CTLs kill HTLV-1-expressing cells and therefore select against integration in transcriptionally active areas of the genome. In addition, integration into genes, which may disrupt their function, reduces the steady state frequency of proviruses in gene-dense regions. These parameters act in concert with host genetic factors [52],[53] to determine an individual's proviral load, level of Tax expression and disease status.

## Materials and Methods

## Patients; blood samples

Samples from 24 individuals infected with Human T-cell Leukaemia Virus 1 (HTLV-1) were analysed. These included 11 ACs and 13 patients with HAM/TSP. All patients attended the HTLV-1 clinic at St Marys Hospital, London and donated their blood having given written informed consent. HTLV-1 infection was confirmed by the presence of antibodies to HTLV-1 Gag and Env antigens in sera by Western blot (HTLV blot 2.4; Genelabs). Diagnosis of HAM/TSP was made following World Health Organisation criteria. PBMCs were isolated from whole blood by density gradient centrifugation using Histopaque-1077 (Sigma). Cells were washed twice in PBS and cryopreserved in fetal calf serum (FCS, Sigma) with 10% dimethyl sulphoxide (DMSO, Sigma) in liquid nitrogen until required. DNA was extracted as described in the manufacturer's protocol (Qiagen, DNeasy Tissue Kit).

## Co-culture Assay

The HTLV-1 producing cell line MT2 was labelled with CD4^+^ microbeads (Miltenyi), stained with CFSE and gamma-irradiated (^137^Cs, 40,000 rads). The cells were then co-cultured with Jurkat cells for 3 hrs at 37°C at a 1∶1 ratio. The MT2 cells were then depleted and an aliquot of the Jurkat cells analysed to verify Gag transmission from the MT2 cells and to quantify MT2 contamination. Contamination was less than 5%. By one week co-culture, all remaining MT2 cells had died. Sixteen days post-culture, DNA was extracted from the Jurkat cells and amplified for HTLV-1 integration sites. To verify integrations were novel and not contaminating MT2 sites, DNA was also extracted from MT2 cells and the resulting integrations were used to search the set of novel integrations for MT2 sites. No contaminating MT2 sites were found.

## Linker-Mediated PCR

In this paper, we refer to integration sites generated by infection in cell culture as *in vitro* sites and those derived from patients' PBMCs as *in vivo* sites. Four µg of extracted DNA was digested with 10 U Nla III (New England Biolabs) in a total volume of 50 µl for 3 hours at 37°C. After purification with the PCR clean-up kit (Qiagen) following manufacturer's instructions, DNA was eluted into 50 µl elution buffer. For the linear linker-mediated PCR for amplification of *in vivo* sites, 20 µl of this DNA solution was incubated for 30 minutes at room temperature with 2 µl Quick Ligase (New England Biolabs) and 40 pmoles of the primer Bio1 [29]. After a further purification, the ligated DNA was eluted into 55 µl, of which 10 µl was used in each of four replicate linear PCR reactions: 100 cycles, using 20 pmoles of the primer Bio2 [29] and the following conditions: 94°C for 10 min; 100 cycles of 95°C for 45 sec, 60°C for 45 sec, 72°C for 2 min; and a final elongation step of 72°C for 10 min. Ten µl of a five-fold dilution of the linear PCR product was used in the classical (bidirectional) PCR reaction. In the bidirectional PCR, DNA was amplified with 20 pmoles each of Bio3 and Bio4 [29] with the following conditions: 94 °C for 10 min; 100 cycles of 95°C for 45 sec, 60°C for 45 sec, 72°C for 2 min; and a final elongation step of 72°C for 10 min.

For amplification of the *in vitro* integration sites, the digested product was ligated to a longer double-stranded NlaIII linker overnight at 16°C. Bidirectional PCR was then carried out using primers in the viral LTR (Bio2) and the linker (AE2814), followed by nested PCR using the primers Bio3 and AE2815 [36].

## Cloning and Sequencing

Two µl of amplified integration site products were combined with 0.5 µl TOPO-TA cloning sequencing vector PCR-4® and used to transform MachT1 cells according to manufacturer's instructions (Invitrogen). Clones were picked and sequenced using the T3 primer (Invitrogen). To check that the amplification was HTLV-1 specific, plasmids were digested with EcoRI, run on a 2% agarose gel and transferred to nylon membrane (Roche) by southern blotting. They were then probed using the Bio5 primer found in the viral LTR [29] conjugated to a single digoxigenin-labelled dideoxyuridine-triphosphate (DIG-ddUTP) (Bio5-DIG) according to manufacturer's instructions (Roche Applied Science). Membranes were pre-hybridised for 30 min at 70°C using DIG EasyHyb (Roche Applied Science) before 4 hours of hybridisation with 10 pmol Bio5-DIG in 7 ml EasyHyb. Membranes were washed twice for 5 min in 2xSSC/ 0.1% SDS and twice for 15 min in 0.5xSSC/ 0.1% SDS at hybridisation temperature. Bound Bio5-DIG was detected using the Roche DIG nucleic acid detection kit following manufacturer's protocol. Results were documented by scanning the membrane once dry.

## Integration site determination

The genomic integration site was located within the cloned sequence by identifying the terminal 5′ sequence of the viral LTR and the junction between the genomic sequence and Bio1. The genomic sequence was mapped on to the hg17 assembly of the human genome using the BLAST-Like alignment tool (BLAT) [54] http://genome.ucsc.edu/cgi-bin/hgBlatcommandstart. A match was defined as a sequence having all 3 of the following:

1. 98% or more homology between the match and the obtained sequence.
2. Homology extending from three bases of the HTLV-1 terminus.
3. Yielded a unique best hit.

A total of 313 sequences from persistently infected individuals and 266 sequences from the *in vitro* co-culture could be mapped to the human genome obeying the above criteria. In addition, 527 sequences from a previously published *in vitro* study were mapped using the same protocol [22]. Integration sites were examined for the occurrence of various chromosomal features using tables available from the University of California, Santa Cruz database [55]. Proviral integration sites can be downloaded from http://bushmanlab.pbwiki.com/f/Meekings.fa.

A set of randomised controls was generated. Ten control sites per integration site were generated, each equidistant from a genomic NlaIII site, to account for any possible bias introduced by a non-random genomic distribution of the restriction enzyme. These sites were analysed in the same manner as the experimental sites.

The statistical method used to compare the experimental sites to MRC sites and the comparison between retroviruses is described in Protocol S1. Software tools and annotated data files are available upon request.

## Staining for CD4/CD8 and Tax

To stain for cell surface markers, treated cells were washed once, fixed with 2% paraformaldehyde (Sigma) for 20 mins at room temperature, washed again and resuspended in 100 µl PBS/ 10% FCS in a ‘V’ bottomed 96-well plate with appropriate antibodies (15 µg/ml phycoerythrin-cyanine 5(PC5)-conjugated anti-CD4 and energy coupled dye (ECD)-conjugated anti-CD8 antibodies (Immunotech)) for 20 mins at room temperature. Cells were permeabilised at room temperature for 10 mins using PBS/ 0.1% Triton X-100 (Sigma). The cells were then washed and resuspended in PBS/7% normal goat serum (NGS, Sigma) with 1∶200 dilution of Lt-4 anti-Tax antibody (gift from Y. Tanaka) for 25 mins at room temperature. Cells were resuspended in PBS ready for flow cytometric analysis. All flow cytometry was done on a Coulter Epics XL (Beckman Coulter) flow cytometer and data analysis done using Coulter Expo 32 software.

## Proviral load measurement

DNA was amplified for HTLV-1 DNA and for β-actin (as a measure of genomic DNA) using the Tax sequence-specific primers SK43 and SK44 [56] and β-actin primers specific for the 5′ and 3′ ends. Three dilutions of neat eluted DNA (1∶4, 1∶8, 1∶16) were amplified by real time quantitative PCR in a Roche light cycler using SYBR® Green 1 Dye incorporation (Roche) and 1 µM of each primer. Incorporation was detected at 85°C at the end of each of the 45 cycles. Standard curves were generated using the rat cell line TARL2 which contains 1 copy per cell of the HTLV-1 provirus [23]. The sample copy number was estimated by interpolation from the standard curve, calculated as an average of the 3 dilutions, and expressed as percentage of PBMCs infected, assuming 1 proviral copy per cell.

## Supporting Information

Figure S1 HTLV-1 integration in vivo and in vitro is identical at the nucleotide level. Integration of HTLV-1 *in vivo* is indistinguishable from that *in vitro* at the nucleotide level (*in vitro* data combined from the co-culture sites obtained in this report and sites reported by Derse et al. [22]). Integration generates a hexameric repeat (figure, box) at the point of integration (indicated by arrow). The HTLV-1 site, analogous to those previously described for other retroviruses, shows palindromic symmetry centred around the middle of the hexameric repeat (dashed line). Strand transfer positions are marked by black arrows. Blue and red boxes show changes ±30% respectively compared to matched random control sites (MRCs). The integration site can also be viewed as a LOGO image where the overall height of the stack represents the sequence conservation at that point and the height of each symbol in the stack represents the frequency of the respective nucleic acid at that point. The HTLV-1 integration site, as well as the integration sites of HIV, SIV and MLV, has a preference for T at position -2 and an A at position +2 after the nucleotide repeat.
(2.88 MB TIF)

Figure S2 The dynamic control of Tax expression *in vivo*. The present study shows that a high proportion of integrations in regions of transcriptional activity is associated with a high rate of proviral (Tax) expression, which in turn is associated with the inflammatory disease HAM/TSP. A low proportion of integrations in regions of transcriptional activity is associated with low Tax expression and asymptomatic infection (AC). The figure depicts the putative selection forces that act on the genomic distribution of integrated proviruses. Tax expression *in vivo* is decreased by the CTL response and by negative selection against gene disruption. Proliferation of the infected cell caused by expression of Tax leads to a positive feedback to increase proviral expression; the intrinsic preference of HTLV-1 to integrate in transcriptionally active regions also increases provirus expression. In this way, there is a dynamic balance *in vivo* acting to determine an individual's level of proviral expression and hence the risk of HTLV-1-associated inflammatory disease.
(1.33 MB TIF)

Protocol S1 Supplementary statistical analyses methods.
(0.69 MB PDF)

Table S1 Classification of individuals used in this study. Identification codes, Tax expression levels (% of CD4+ T cells expressing Tax), Proviral loads (number of provirus positive cells per 100 PBMCs), disease status and number of contributing sites are given.
(0.05 MB DOC)
