# Supplementary figures and images for: HTLV-1 Integration into Transcriptionally Active Genomic Regions Is Associated with Proviral Expression and with HAM/TSP
Source: PLoS Pathog. 2008 Mar 21;4(3):e1000027. doi: 10.1371/journal.ppat.1000027 (PMC2265437; doi:10.1371/journal.ppat.1000027)

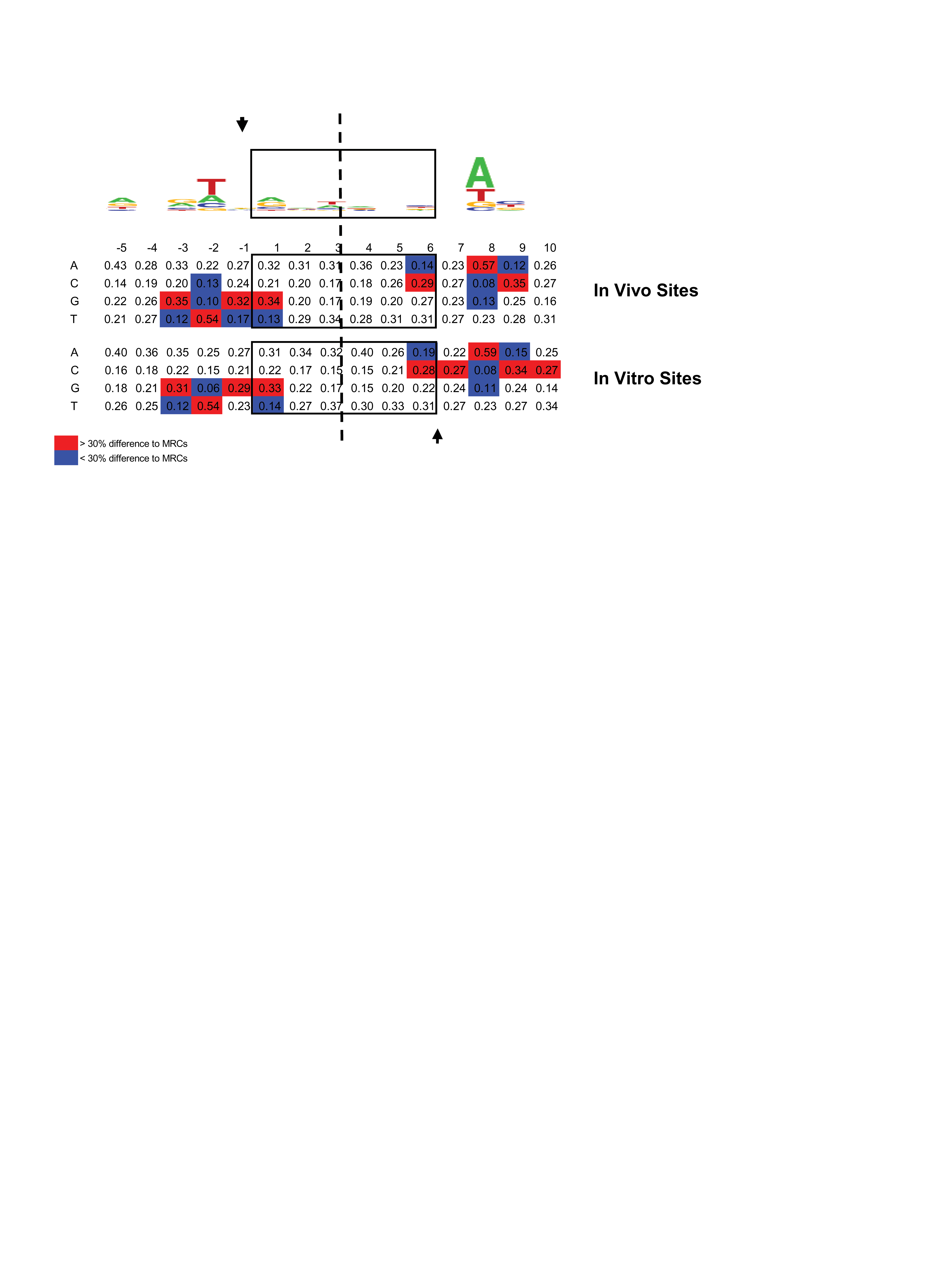

Supplement: Figure S1 — HTLV-1 integration in vivo and in vitro is identical at the nucleotide level. Integration of HTLV-1 in vivo is indistinguishable from that in vitro at the nucleotide level (in vitro data combined from the co-culture sites obtained in this report and sites reported by Derse et al. [22]). Integration generates a hexameric repeat (figure, box) at the point of integration (indicated by arrow). The HTLV-1 site, analogous to those previously described for other retroviruses, shows palindromic symmetry centred around the middle of the hexameric repeat (dashed line). Strand transfer positions are marked by black arrows. Blue and red boxes show changes ±30% respectively compared to matched random control sites (MRCs). The integration site can also be viewed as a LOGO image where the overall height of the stack represents the sequence conservation at that point and the height of each symbol in the stack represents the frequency of the respective nucleic acid at that point. The HTLV-1 integration site, as well as the integration sites of HIV, SIV and MLV, has a preference for T at position -2 and an A at position +2 after the nucleotide repeat. (2.88 MB TIF) [file ppat.1000027.s001.tif]

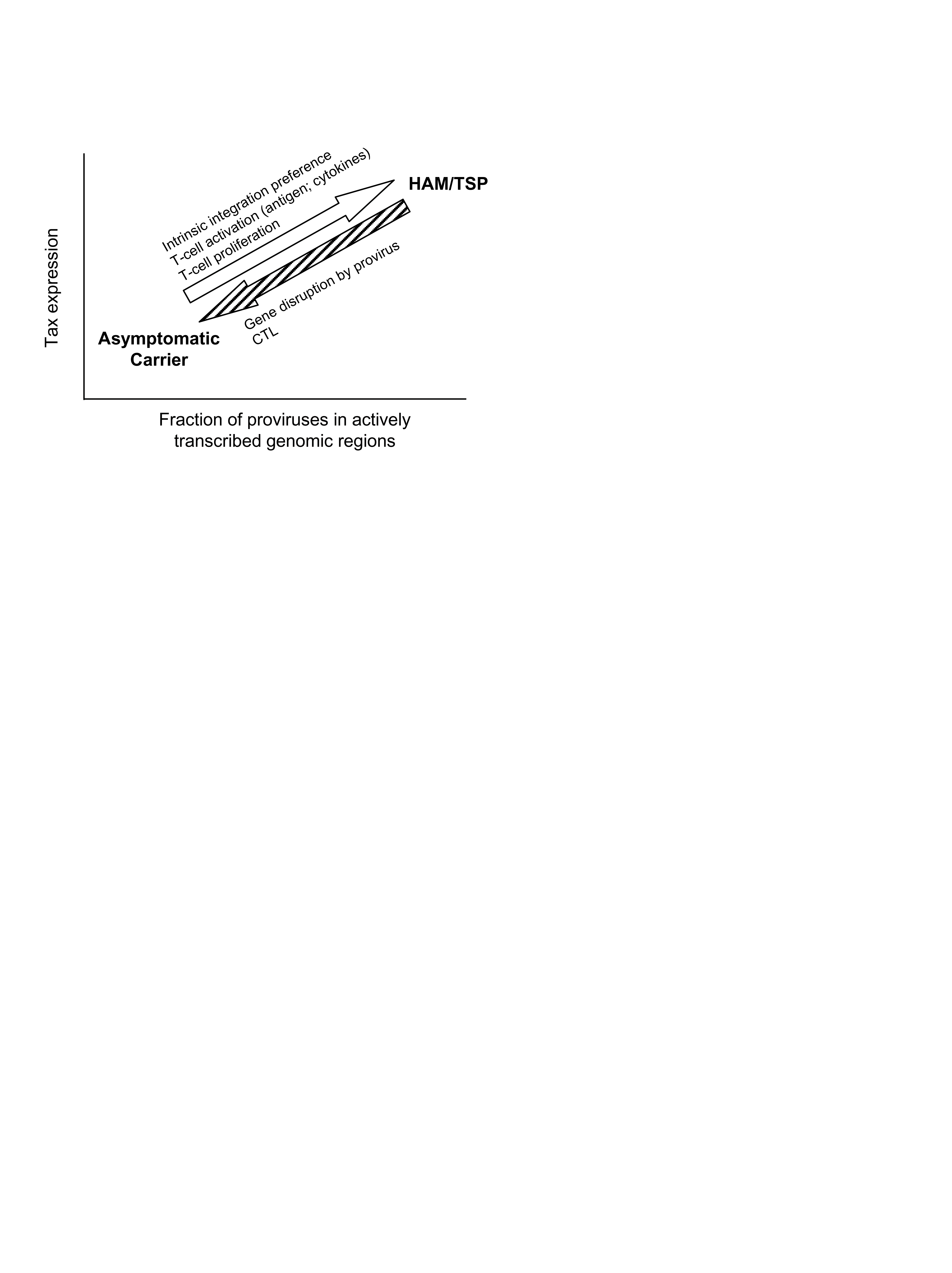

Supplement: Figure S2 — The dynamic control of Tax expression in vivo. The present study shows that a high proportion of integrations in regions of transcriptional activity is associated with a high rate of proviral (Tax) expression, which in turn is associated with the inflammatory disease HAM/TSP. A low proportion of integrations in regions of transcriptional activity is associated with low Tax expression and asymptomatic infection (AC). The figure depicts the putative selection forces that act on the genomic distribution of integrated proviruses. Tax expression in vivo is decreased by the CTL response and by negative selection against gene disruption. Proliferation of the infected cell caused by expression of Tax leads to a positive feedback to increase proviral expression; the intrinsic preference of HTLV-1 to integrate in transcriptionally active regions also increases provirus expression. In this way, there is a dynamic balance in vivo acting to determine an individual's level of proviral expression and hence the risk of HTLV-1-associated inflammatory disease. (1.33 MB TIF) [file ppat.1000027.s002.tif]
