# Supplementary material for: HTLV-1 Integration into Transcriptionally Active Genomic Regions Is Associated with Proviral Expression and with HAM/TSP
Source: PLoS Pathog. 2008 Mar 21;4(3):e1000027. doi: 10.1371/journal.ppat.1000027 (PMC2265437; doi:10.1371/journal.ppat.1000027)
Supplement: Protocol S1 — Supplementary statistical analyses methods. (0.69 MB PDF) [file ppat.1000027.s003.pdf]

Association of Genomic Features with Integration

June 22, 2007

Contents

1 Introduction **2**

2 Preference for Genes **3**
2.1 Acembly Genes 3
2.2 refGenes 5
2.3 ensGenes 7
2.4 genScan Genes 9
2.5 uniGenes 11
2.6 oncogenes 13

3 CpG Island Neighborhoods **14**
3.1 1 kilobase neighborhoods 14
3.2 5 kilobase neighborhoods 15
3.3 10 kilobase neighborhoods 16
3.4 25 kilobase neighborhoods 17
3.5 50 kilobase neighborhoods 18

4 Gene Density, Expression 'Density', and CpG Island Density **19**
4.1 25 kiloBase Window 20
4.2 50 kiloBase Window 26
4.3 100 kilobase Window 31
4.4 100 kiloBase Window 35
4.5 250 kiloBase Window 40
4.6 500 kilobase Window 45
4.7 500 kiloBase Window 49
4.8 1 megabase Window 54
4.9 1 megaBase Window 58
4.10 2 megabase Window 63
4.11 2 megaBase Window 67
4.12 4 megaBase Window 72
4.13 8 megaBase Window 77
4.14 16 megaBase Window 82
4.15 32 megaBase Window 87

5 Juxtaposition with Gene Start and End Positions **92**
5.1 Acembly Annotations 92
5.2 RefSeq Annotations 96
5.3 genScan Annotations 100
5.4 uniGene Annotations 104

6 GC content **108**

7 Cytobands **125**

1 Introduction

In this document, I examine the association of integration siting with various genomic features.

The numbers are shown below:

```
Origin.of.data.set
     MRC-inVivo Meekings-inVivo
           3110             313
```

The distribution of relative frequency of insertions across the chromosomes is given in this barplot:

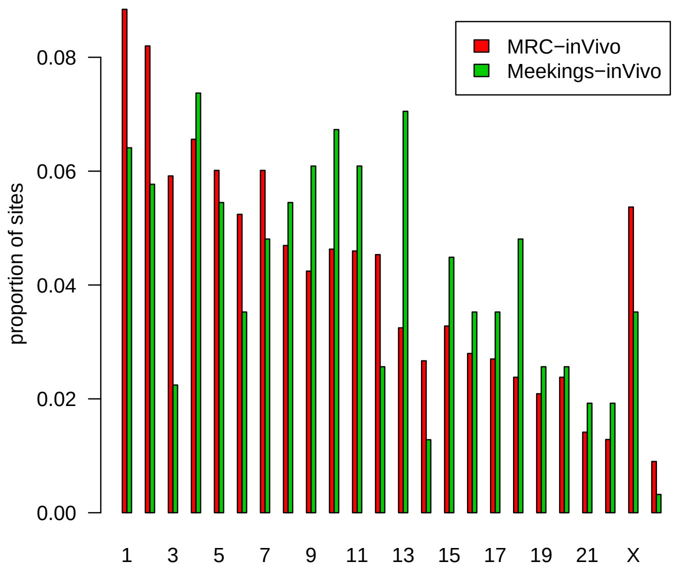

Are there chromosomes that are particularly favored for integration by one group over the other? This was tested for statistical significance. The test performed used the likelihood ratio statistic for the logistic regression model (reviewed in [1]) as implemented by the `glm` function of R using the `binomial` family. The null hypothesis tested is the ratio of true integration events in the two groups is constant across all chromosomes. This test attains a p-value of 0.0011709.

2 Preference for Genes

2.1 Acembly Genes

Here we examine the relative preference that integration events in the two groups have for genes. In the following plot we show the relative frequency of integrations in genes according to the 'Acembly' anotation. The bars grouped over the label "In Gene" give the relative frequency of integration events (compared to control sites) between bases located within Acembly gene annotations, while the label "Not in Gene" give the relative frequency of integration events (compared to control sites) between bases not located within Acembly gene annotations.

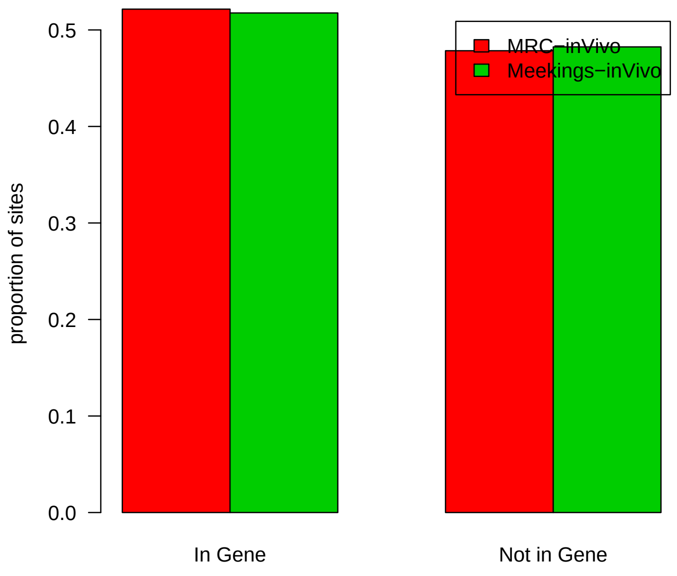

Is there is a difference in the tendency for insertions to occur in genes? A formal test of significance yields a p-value of 0.89336. In the following plot we show the relative frequency of insertions in exons according to the 'Acembly' anotation The bars grouped over the label "In Exon" give the relative frequency of integration events (compared to control sites) between bases located in exons according to the Acembly annotation, while the label "Not in Exon" give the relative frequency of integration events (compared to control sites) between bases not located in exons according to the Acembly gene annotation.

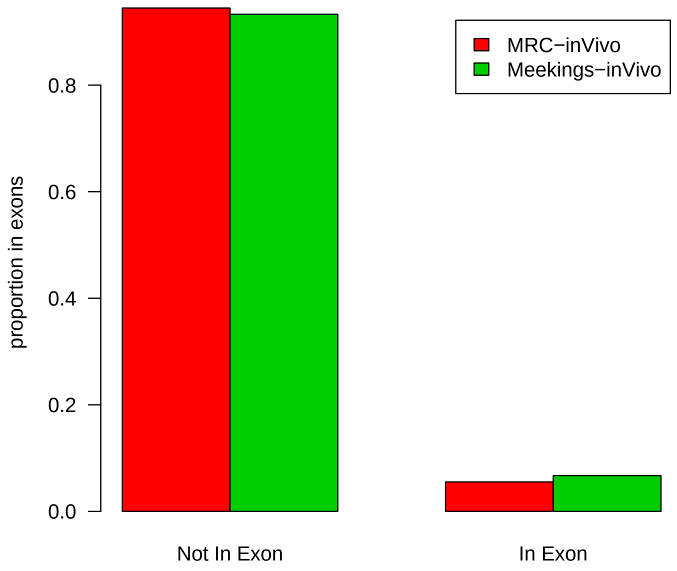

Here is the table of coefficients of the log ratio of intensities along with their standard errors, z statistics, and p-values:

	coef	se	z	p
(Intercept)	-2.2900	0.0854	-26.800	4.47e-158
in.gene	-0.0427	0.1230	-0.347	7.28e-01
in.exon	0.2280	0.2470	0.920	3.58e-01

The model on which these coefficients are based include terms for whether the site is in a gene or not. Thus, the effect shown as 'in.exon' is net of that due to being in a gene. Note that in the barplot above the 'Not in Exon' bars include the both introns and intergenic regions, so the impression given by the table may differ from that for the barplot.

2.2 refGenes

Here we examine the relative preference that insertions of the two types have for genes. In the following plot we show the relative frequency of insertions in genes according to the 'refGene' anotation.

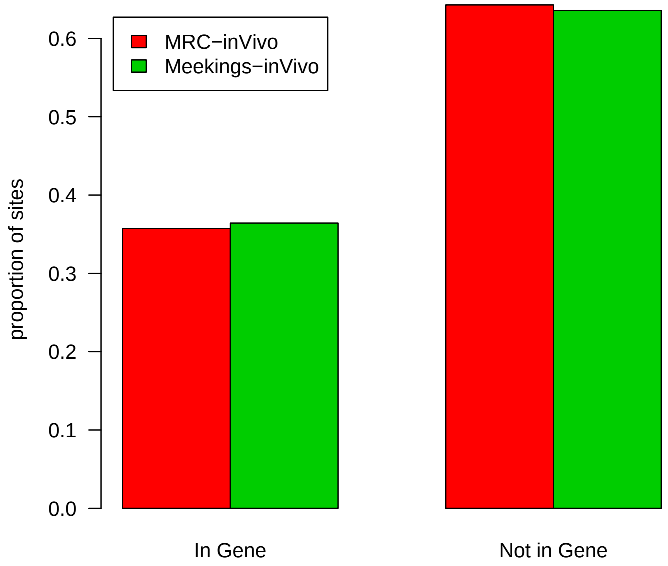

Is there is a tendency for insertions to occur in genes? A formal test of significance yields a p-value of 0.80619.

In the following plot we show the relative frequency of insertions in exons according to the 'refGene' anotation.

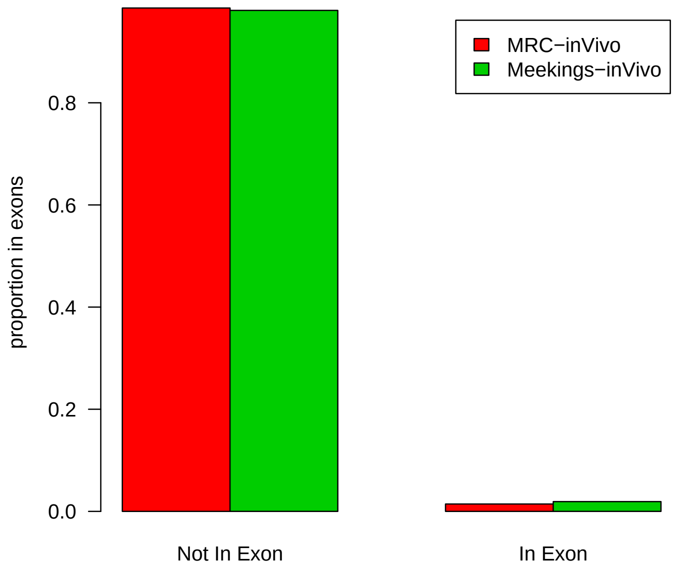

Here is the table of coefficients of the log ratio of intensities for along with their standard errors, z statistics, and p-values:

	coef	se	z	p
(Intercept)	-2.3100	0.0743	-31.000	1.69e-211
in.gene	0.0176	0.1250	0.140	8.89e-01
in.exon	0.2750	0.4460	0.616	5.38e-01

The model on which these coefficients are based include terms for whether the site is in a gene or not. Thus, the effect shown as 'in.exon' is net of that due to being in a gene. Note that in the barplot above the 'Not in Exon' bars include the both introns and intergenic regions, so the impression given by the table may differ from that for the barplot.

2.3 ensGenes

Here we examine the relative preference that insertions of the two types have for genes. In the following plot we show the relative frequency of insertions in genes according to the 'ensGene' anotation.

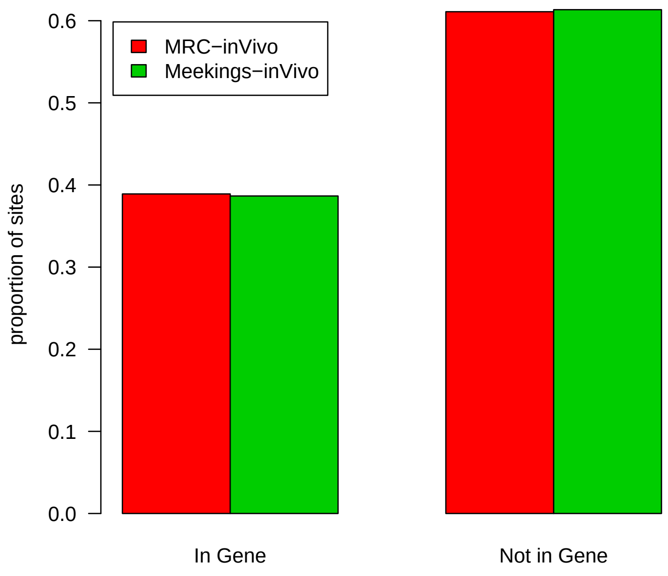

Is there is a tendency for insertions to occur in genes? A formal test of significance yields a p-value of 0.93144.

In the following plot we show the relative frequency of insertions in exons according to the 'ensGene' anotation.

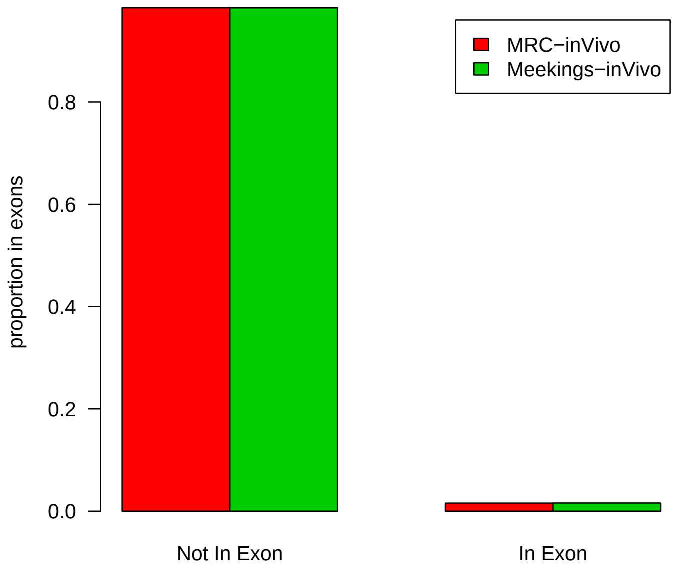

Here is the table of coefficients of the log ratio of intensities for along with their standard errors, z statistics, and p-values:

	coef	se	z	p
(Intercept)	-2.2900	0.0757	-30.3000	3.03e-201
in.gene	-0.0113	0.1230	-0.0919	9.27e-01
in.exon	0.0211	0.4790	0.0439	9.65e-01

The model on which these coefficients are based include terms for whether the site is in a gene or not. Thus, the effect shown as 'in.exon' is net of that due to being in a gene. Note that in the barplot above the 'Not in Exon' bars include the both introns and intergenic regions, so the impression given by the table may differ from that for the barplot.

2.4 genScan Genes

Here we examine the p erence that insertions have for genes. In the following plot we show the relative frequency of insertions in genes according to the 'genScan' anotation.

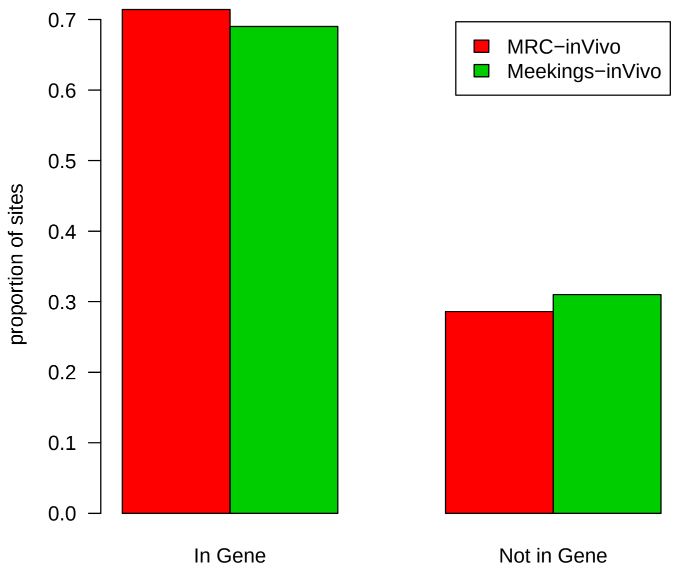

Is there is a tendency for insertions to occur in genes? A formal test of significance yields a p-value of 0.37354.

In the following plot we show the relative frequency of insertions in exons according to the 'genScan' anotation.

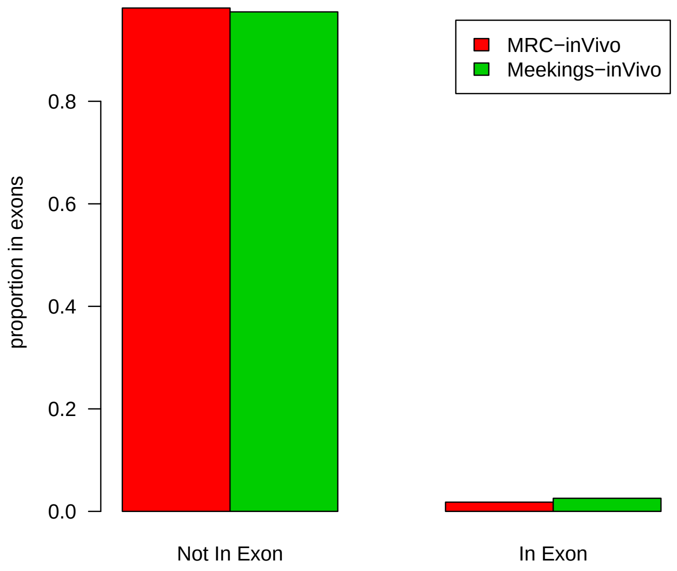

Here is the table of coefficients of the log ratio of intensities along with their standard errors, z statistics, and p-values:

	coef	se	z	p
(Intercept)	-2.220	0.107	-20.700	2.38e-95
in.gene	-0.127	0.129	-0.985	3.25e-01
in.exon	0.397	0.385	1.030	3.03e-01

The model on which these coefficients are based include terms for whether the site is in a gene or not. Thus, the effect shown as 'in.exon' is net of that due to being in a gene. Note that in the barplot above the 'Not in Exon' bars include the both introns and intergenic regions, so the impression given by the table may differ from that for the barplot.

2.5 uniGenes

Here we examine the preference that insertions have for genes. In the following plot we show the relative frequency of insertions in genes according to the 'uniGene' anotation.

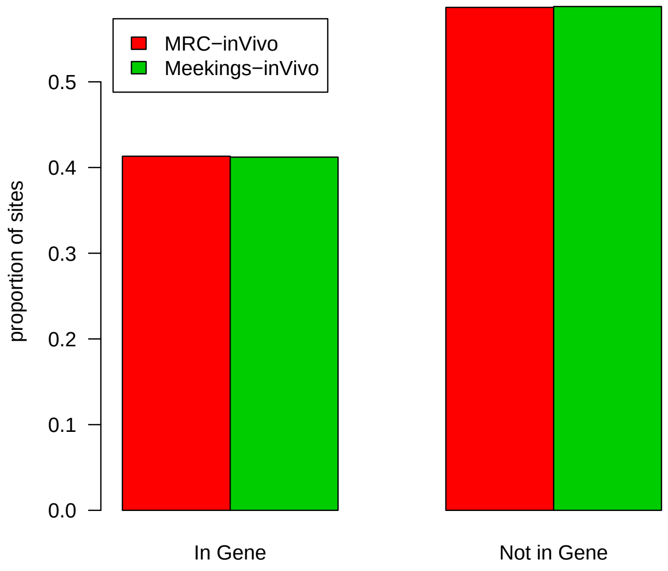

Is there is a tendency for insertions to occur in genes? A formal test of significance yields a p-value of 0.97151.

In the following plot we show the relative frequency of insertions in exons according to the 'uniGene' anotation.

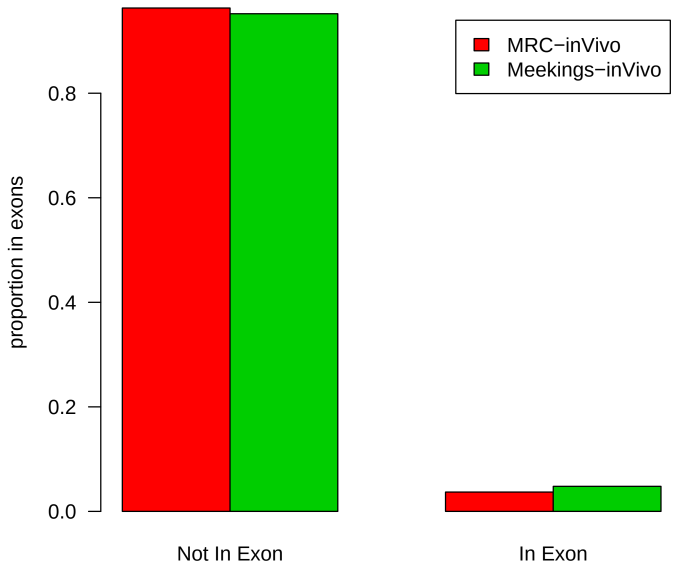

Here is the table of coefficients of the log ratio of intensities along with their standard errors, z statistics, and p-values:

	coef	se	z	p
(Intercept)	-2.2900	0.0773	-29.700	2.29e-193
in.gene	-0.0342	0.1250	-0.273	7.85e-01
in.exon	0.2920	0.2920	1.000	3.17e-01

The model on which these coefficients are based include terms for whether the site is in a gene or not. Thus, the effect shown as 'in.exon' is net of that due to being in a gene. Note that in the barplot above the 'Not in Exon' bars include the both introns and intergenic regions, so the impression given by the table may differ from that for the barplot.

2.6 oncogenes

Here we examine the preference that insertions have for oncogenes. In the following plot we show the relative frequency of insertions within 50kb of an oncogene 5' end.

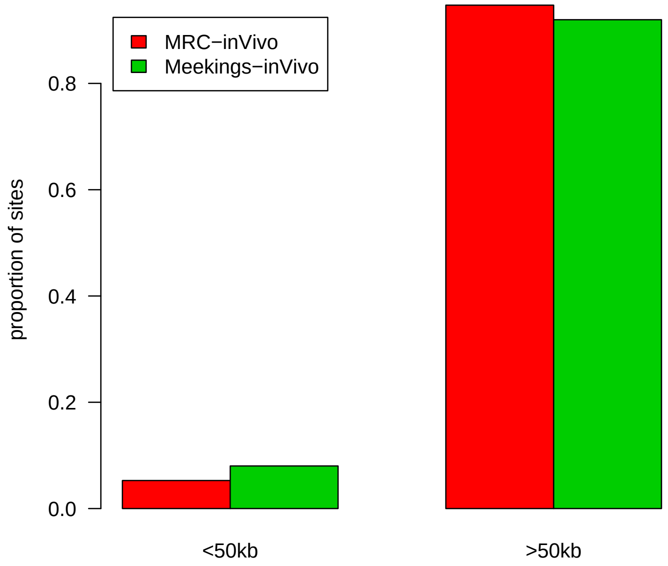

It seems evident that there is a strong tendency for insertions to occur near oncogenes. A formal test of significance bears this out with a p-value of 0.055964.

Here is the table of coefficients of the log ratio of intensities for true insertion sites versus control insertion sites along with their standard errors, z statistics, and p-values for each data set:

	coef	se	z	p
(Intercept)	-2.330	0.0618	-37.7	0.0000
eval(the.gene)TRUE	0.448	0.2230	2.0	0.0451

3 CpG Island Neighborhoods

Here we study the effect of being in the neighborhood of CpG Islands. Following Wu et al [2], who found that the neighborhoods within ±1kb of CpG islands are enriched for MLV insertions, we study such neighborhoods.

3.1 1 kilobase neighborhoods

The following plot shows the effect of being in or within ±1kb of a CpG island:

NULL

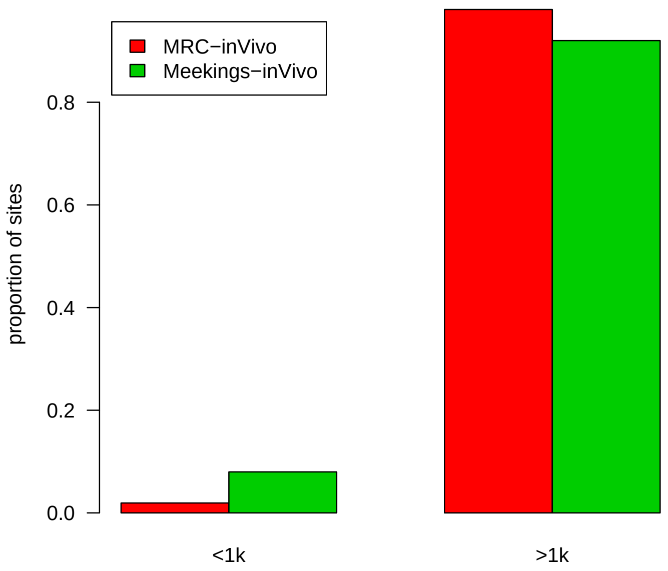

A formal test of significance comparing the difference attains a p-value of $6.4805e - 08$.

3.2 5 kilobase neighborhoods

The following plot shows the effect of being in or within $\pm$5kb of a CpG island:

NULL

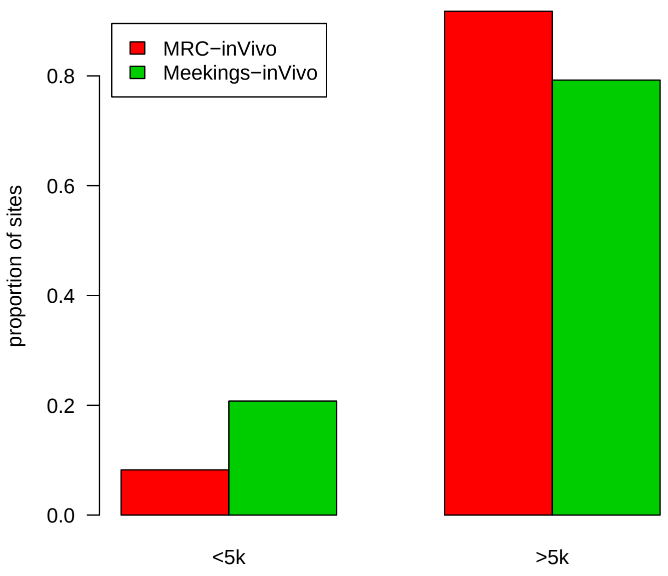

A formal test of significance comparing the difference attains a p-value of $1.0465e - 10$.

3.3 10 kilobase neighborhoods

The following plot shows the effect of being in or within ± 10kb of a CpG island:

```
NULL
```

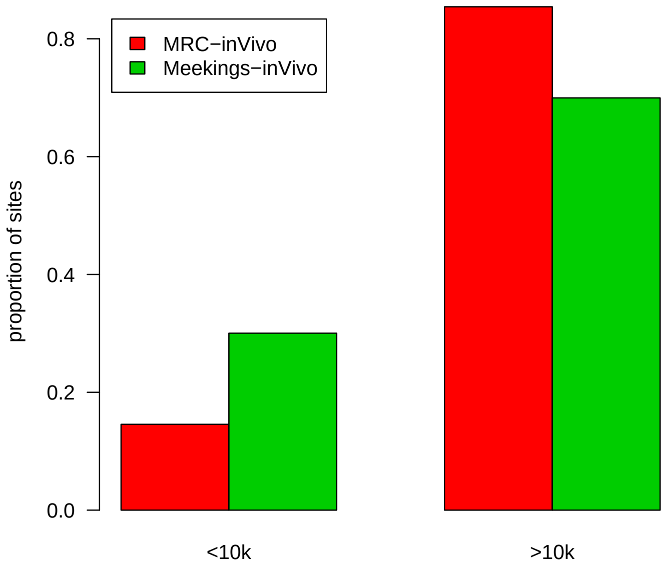

A formal test of significance comparing the difference attains a p-value of $4.9409e - 11$.

3.4 25 kilobase neighborhoods

The following plot shows the effect of being in or within ±25kb of a CpG island:

NULL

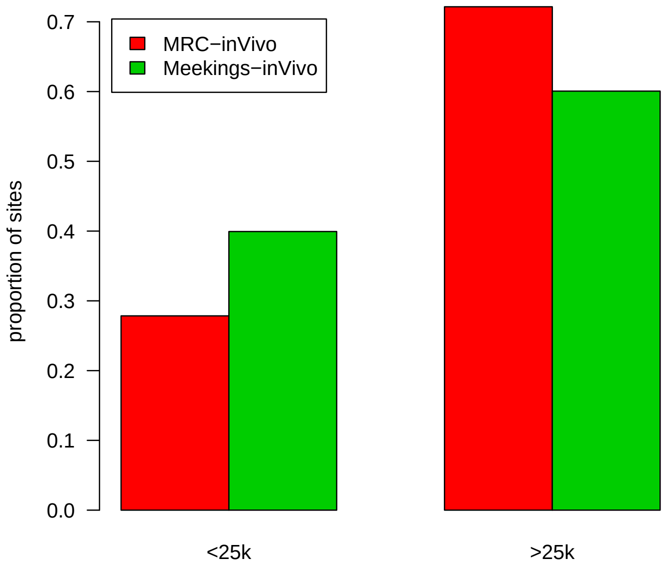

A formal test of significance comparing the difference attains a p-value of $1.2228e-05$.

3.5 50 kilobase neighborhoods

The following plot shows the effect of being in or within ±50kb of a CpG island:

```
NULL
```

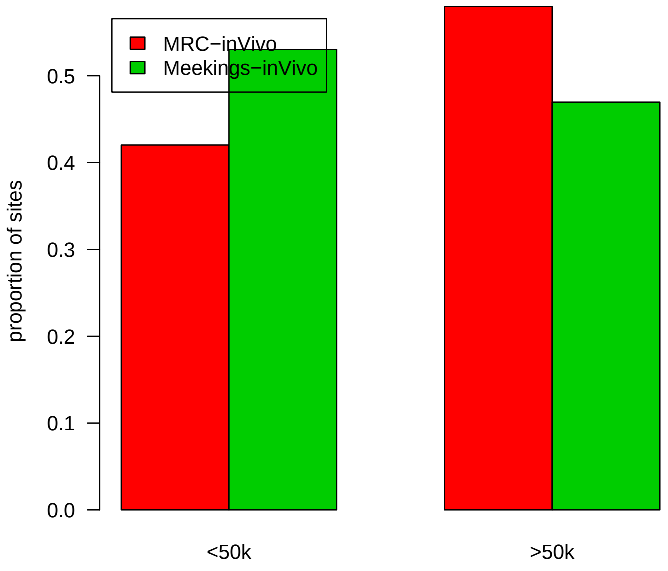

A formal test of significance comparing the difference attains a p-value of 0.00019175.

4 Gene Density, Expression 'Density', and CpG Island Density

In this section the association with gene density is examined. The 'genes' that are counted are the genes represented on the microarray. In addition, we the number of such genes expressed at various levels. The levels are

low.ex Count genes whose expression is in the upper half and divide by number of bases

med.ex Count genes whose expression is in the upper half $1/8^{th}$ and divide by number of bases

high.ex Count genes whose expression is in the upper half $1/16^{th}$ and divide by number of bases

The bolded terms are used as abbreviations in what follows. The abbreviation **dens** is used to indicate gene density as number of genes per base.

4.1 25 kiloBase Window

In the barplot that follows we examine the association of insertion sites with gene density in a 25 kilobase window surrounding each locus. More such plots will follow and the method of their construction is always to try to divide the data according to the deciles of density. However, it often happens that there is a very skewed distribution of density and often even the 90th percentile is zero. In that case, the barplots simply show the sites for which the density is zero and those for which it is non-zero. If there are fewer than ten groups of bars, then the groupings contain ten percent of the sites each except for the leftmost grouping which will contain all of the remaining sites.

Also note that the title of the plot contains clues as to its content; the prefix indicates the type of variable studied while the suffix indicates the window width in the number of bases. The p-value given is the result of fitting a cubic polynomial to the gene density values.

The following expression data and probe set were used for this report:

```
[1] "Jurkat-HU133Plus2"

[1] "HG-U133"

 Category limits

       lower category     upper
1 -0.2551140  group.1 0.6040327
2  0.6040327  group.2 0.7632963
3  0.7632963  group.3 0.9997078
```

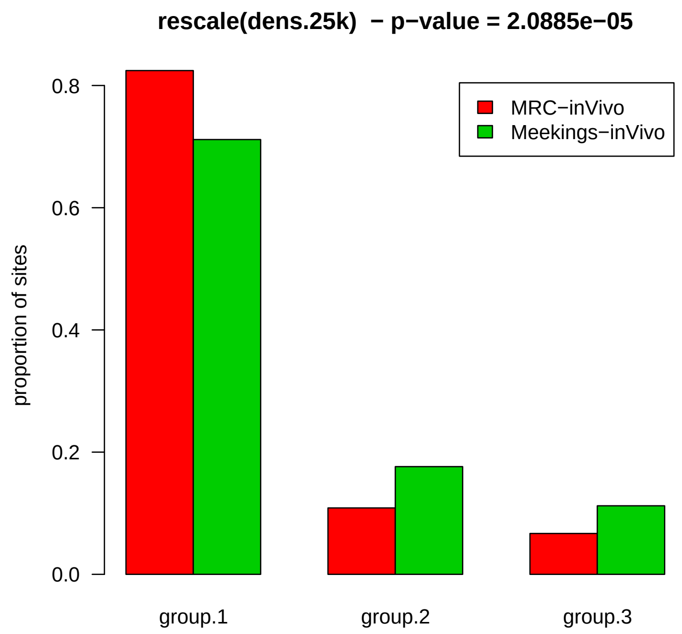
rescale(dens.25k) − p−value = 2.0885e−05
MRC−inVivo
Meekings−inVivo
proportion of sites
0.0
0.2
0.4
0.6
0.8
group.1
group.2
group.3

Here are the results for expression density. First, we count just genes that are in the upper half.

```
Category limits

       lower category     upper
1 -0.1417300  group.1 0.7925190
2  0.7925190  group.2 0.9994155
```

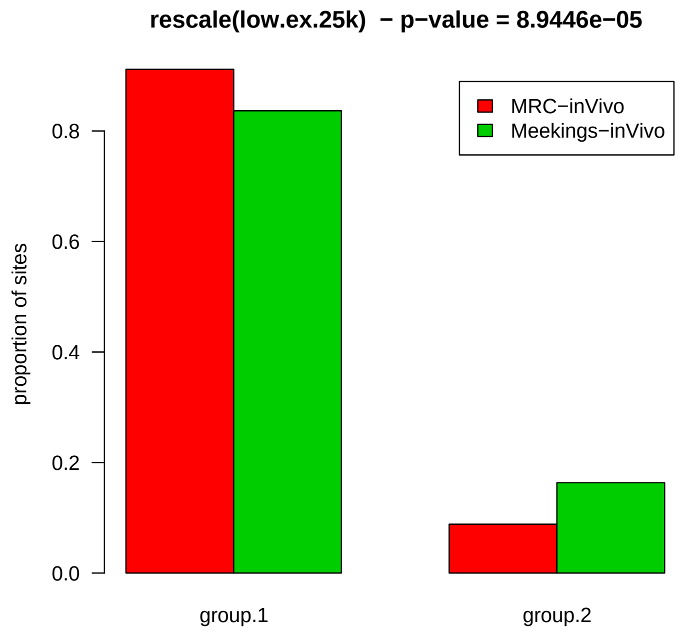

Now we count genes in the upper $1/8^{th}$:

```
Category limits

      lower category     upper
1 -0.078609  group.1 0.8930450
2  0.893045  group.2 0.9997078
```

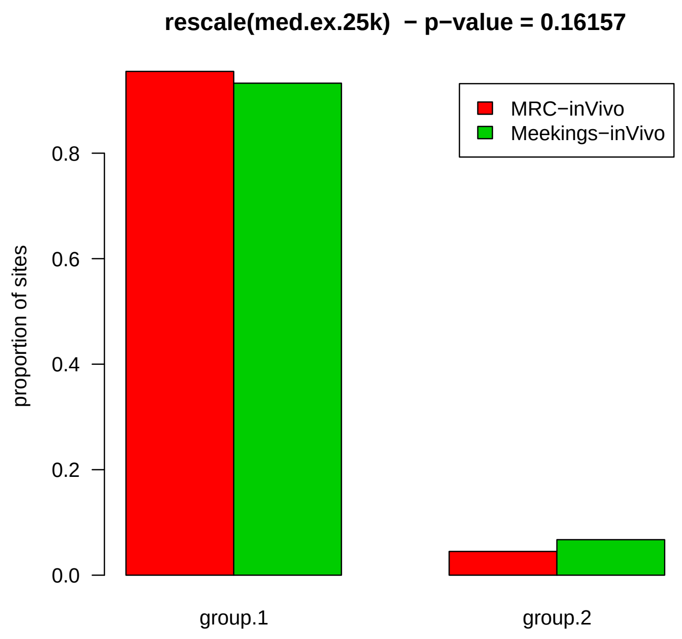

And here we count genes in the upper $1/16^{th}$:

```
Category limits

        lower category     upper
1 -0.04441847  group.1 0.9450614
2  0.94506137  group.2 0.9997078
```

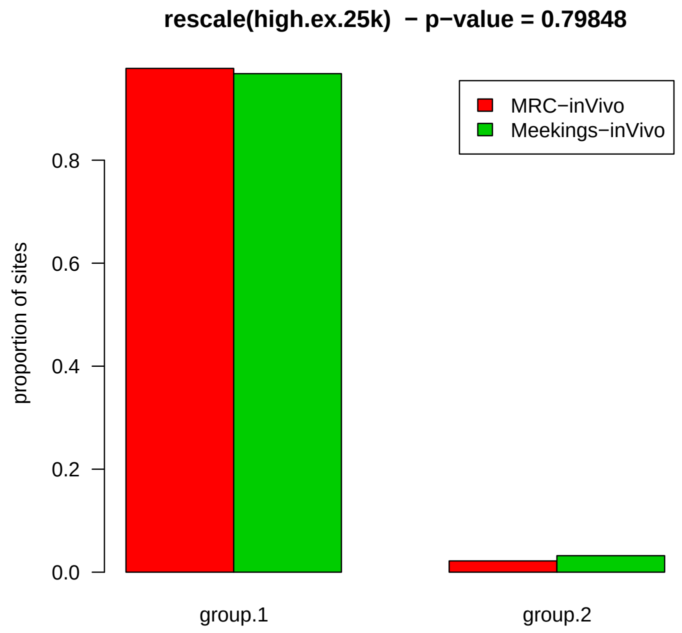

Here the effect of density of CpG islands is studied:

```
Category limits

       lower category     upper
1 -0.2857978  group.1 0.6019871
2  0.6019871  group.2 0.8369375
3  0.8369375  group.3 0.9997078
```

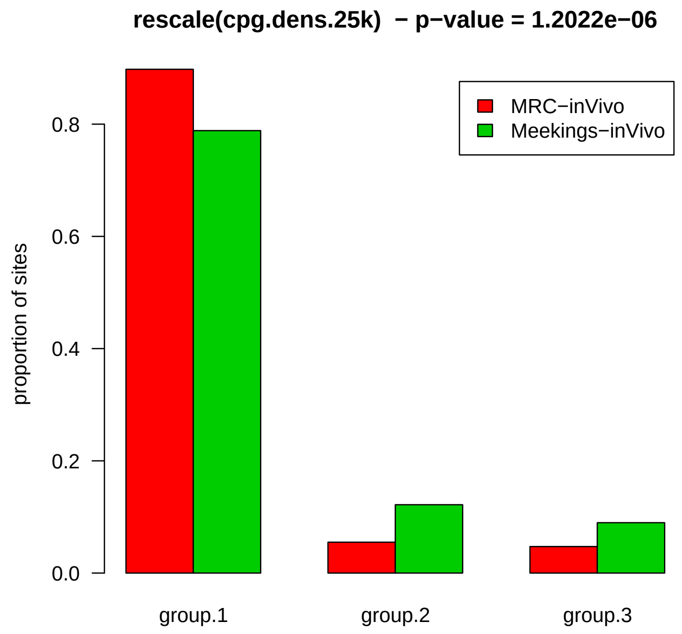

4.2 50 kiloBase Window

In the barplot that follows we examine the association of insertion sites with expression density in a 50 kilobase window surrounding each locus. First, we count just the number of genes on the represented on the chip.

```
 Category limits

       lower category     upper
1 -0.3924605  group.1 0.4021333
2  0.4021333  group.2 0.5558153
3  0.5558153  group.3 0.8158971
4  0.8158971  group.4 0.9997078
```

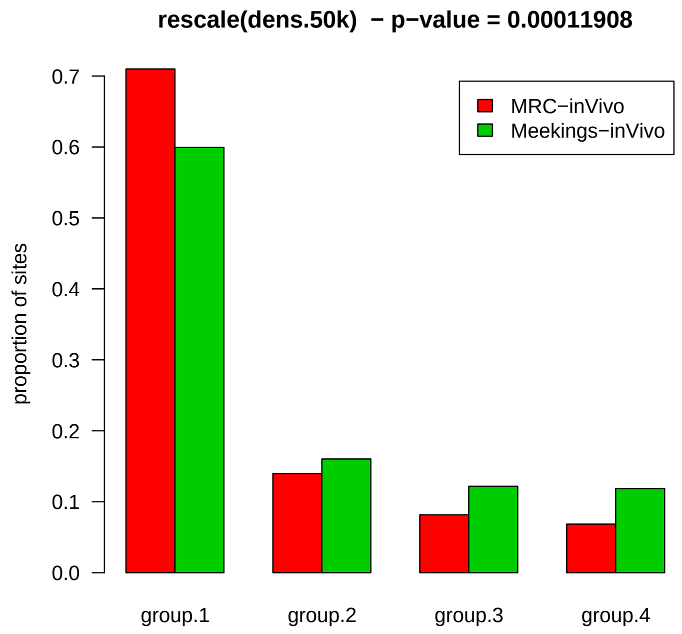

Here are the results for expression density. First, we count just genes that are in the upper half.

```
 Category limits

       lower category     upper
1 -0.2270602  group.1 0.5996493
2  0.5996493  group.2 0.7808299
3  0.7808299  group.3 0.9997078
```

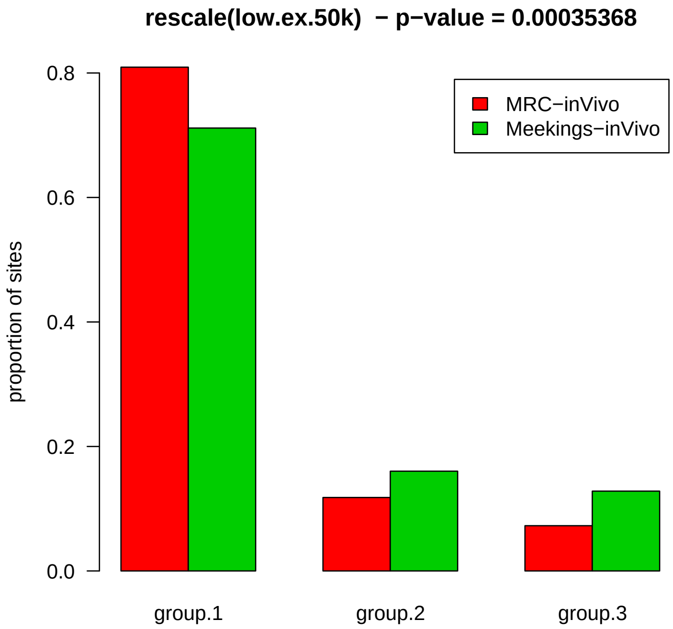

Now we count genes in the upper $1/8^{th}$:

```
 Category limits

       lower category     upper
1 -0.1449445  group.1 0.8027469
2  0.8027469  group.2 0.9997078
```

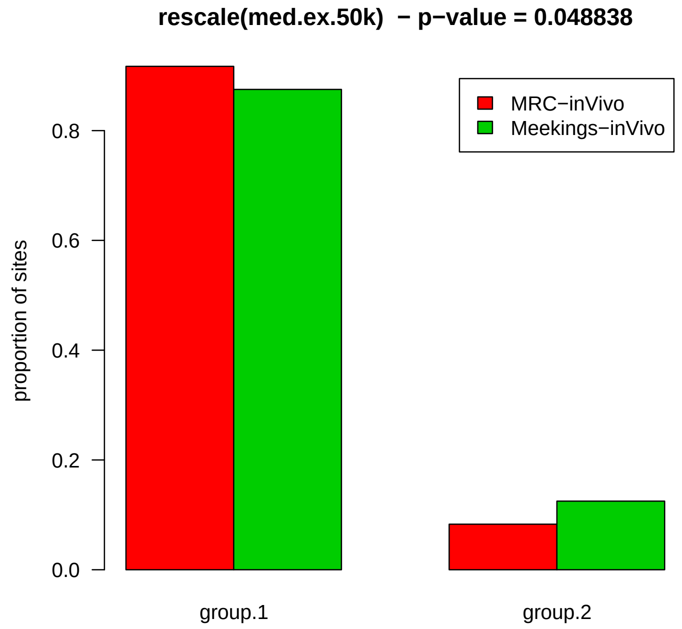

And here we count genes in the upper $1/16^{th}$:

```
 Category limits

        lower category     upper
1 -0.08503799  group.1 0.8971362
2  0.89713618  group.2 0.9997078
```

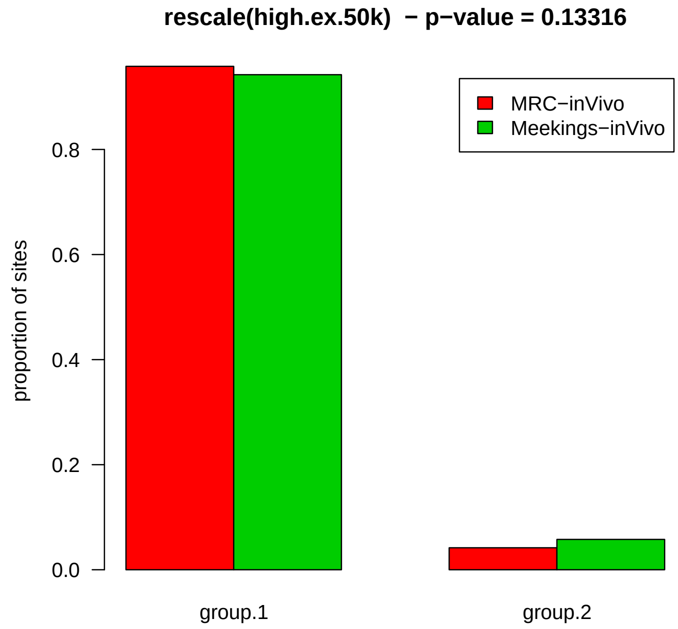

Here the effect of density of CpG islands is studied:

```
Category limits

      lower category     upper
1 -0.4272355  group.1 0.3547633
2  0.3547633  group.2 0.6636470
3  0.6636470  group.3 0.8080070
4  0.8080070  group.4 0.9997078
```

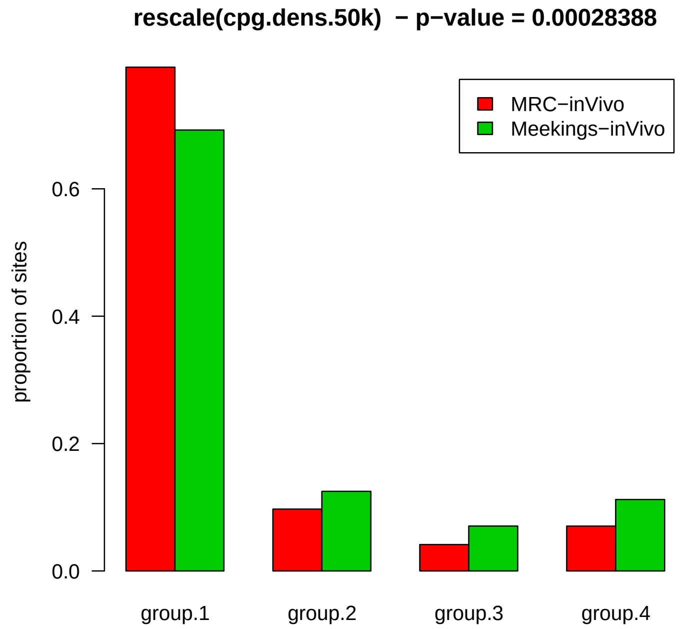

4.3 100 kilobase Window

Here are gene densities for the various gene calls

```
 Category limits

       lower category     upper
1 -0.4029807  group.1 0.4111631
2  0.4111631  group.2 0.8334307
3  0.8334307  group.3 0.9997078
```

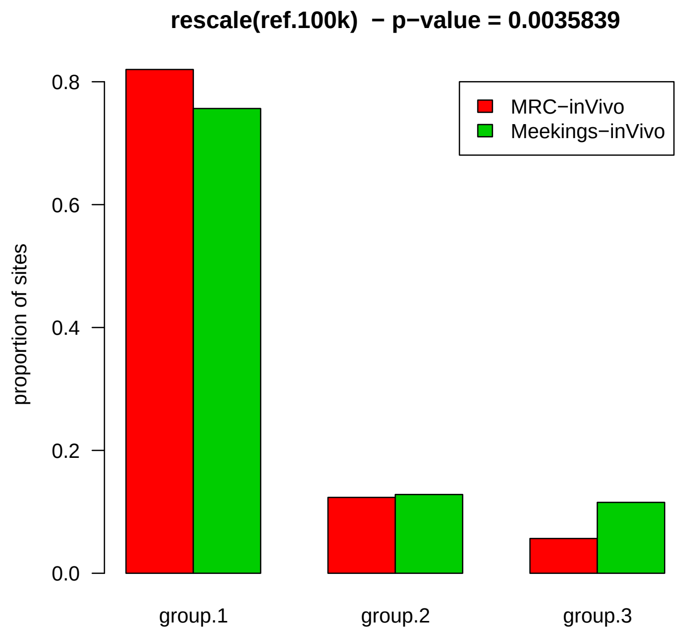

```
Category limits

       lower category     upper
1 -0.4812975  group.1 0.2337814
2  0.2337814  group.2 0.5464641
3  0.5464641  group.3 0.8290473
4  0.8290473  group.4 0.9997078
```

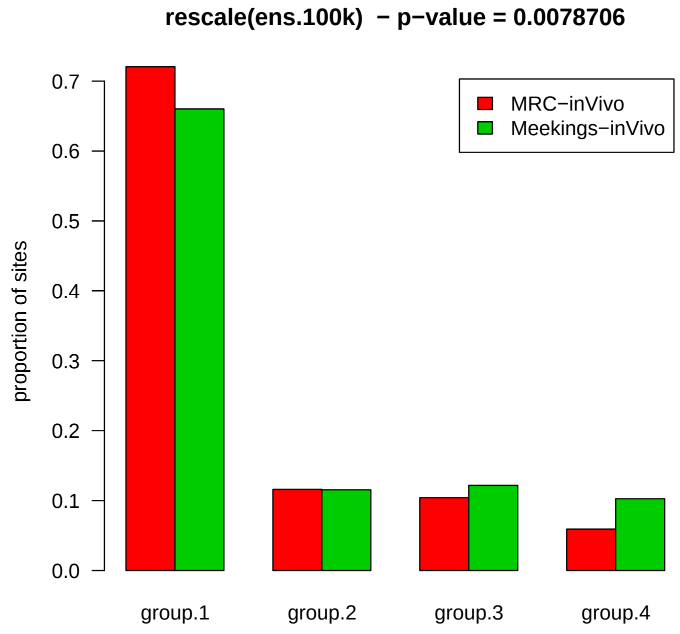

Category limits

	lower	category	upper
1	-0.75336061	group.1	-0.75336061
2	-0.75336061	group.2	-0.34687317
3	-0.34687317	group.3	-0.07188778
4	-0.07188778	group.4	0.12887200
5	0.12887200	group.5	0.41729982
6	0.41729982	group.6	0.62887200
7	0.62887200	group.7	0.78112215
8	0.78112215	group.8	0.99970777

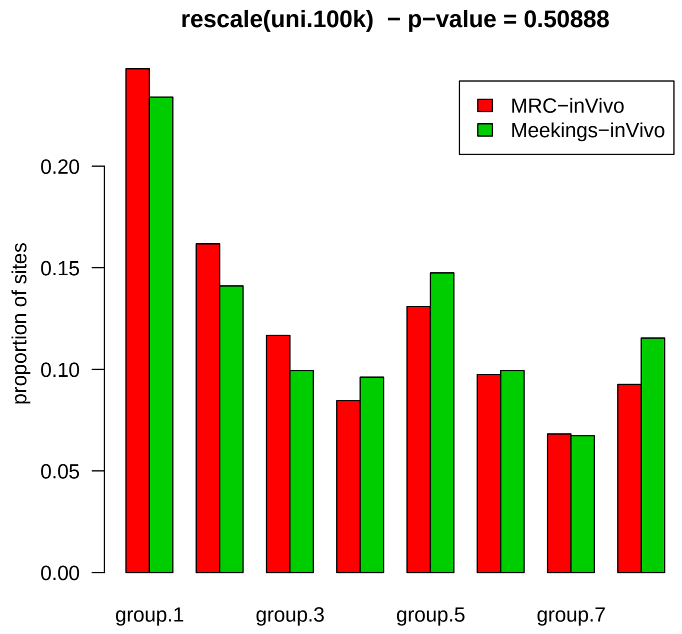

Category limits

	lower	category	upper
1	-0.8232028	group.1	-0.2308591
2	-0.2308591	group.2	0.4164231
3	0.4164231	group.3	0.7495617
4	0.7495617	group.4	0.9997078

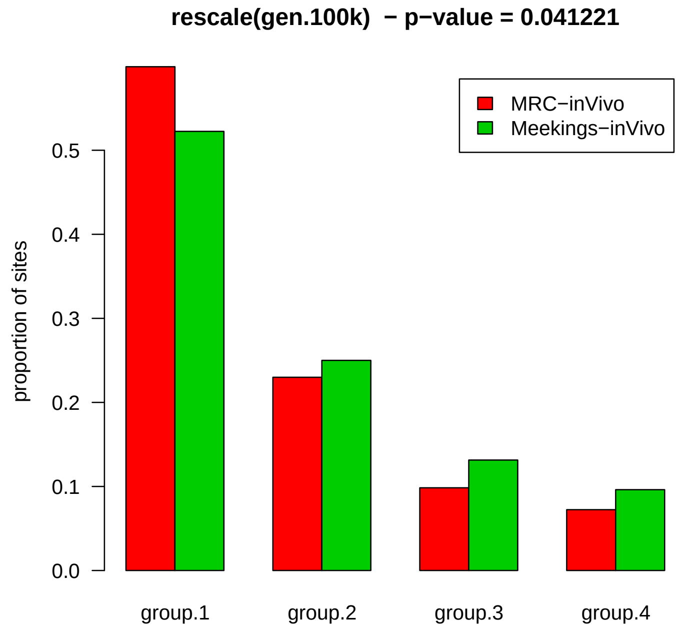

4.4 100 kiloBase Window

In the barplot that follows we examine the association of insertion sites with expression density in a 100 kilobase window surrounding each locus. First, we count just the number of genes on the represented on the chip.

```
 Category limits

       lower category     upper
1 -0.5505552  group.1 0.0000000
2  0.0000000  group.2 0.2469316
3  0.2469316  group.3 0.4000877
4  0.4000877  group.4 0.5850380
5  0.5850380  group.5 0.7998247
6  0.7998247  group.6 0.9997078
```

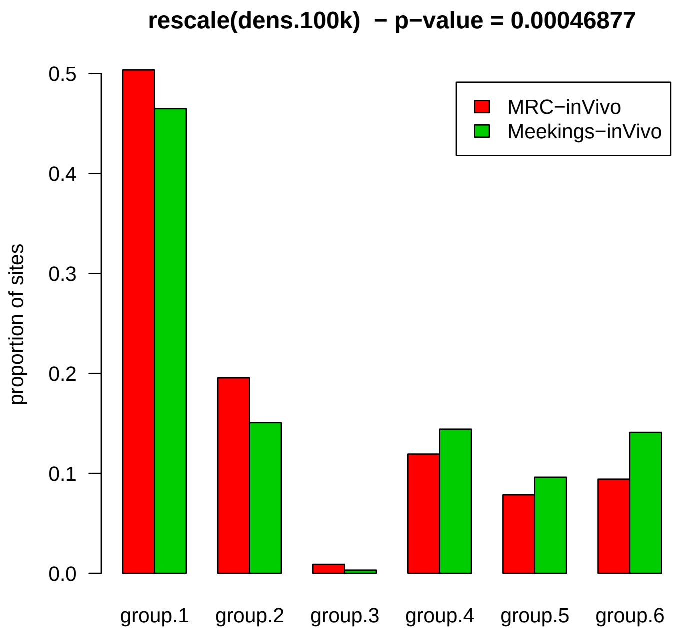

Here are the results for expression density. First, we count just genes that are in the upper half.

```
 Category limits

       lower  category     upper
1 -0.3451198  group.1 0.3998247
2  0.3998247  group.2 0.6092928
3  0.6092928  group.3 0.8275862
4  0.8275862  group.4 0.9997078
```

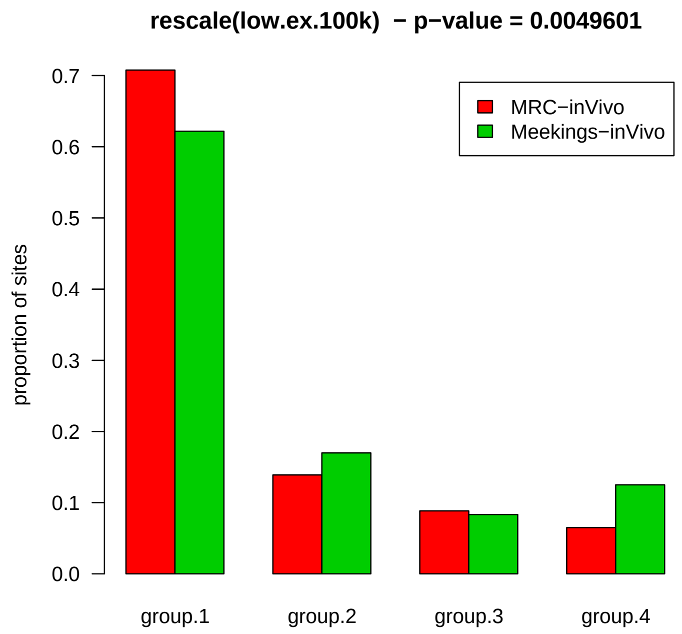

Now we count genes in the upper $1/8^{th}$:

```
Category limits

       lower category     upper
1 -0.2375804  group.1 0.5990649
2  0.5990649  group.2 0.7983635
3  0.7983635  group.3 0.9997078
```

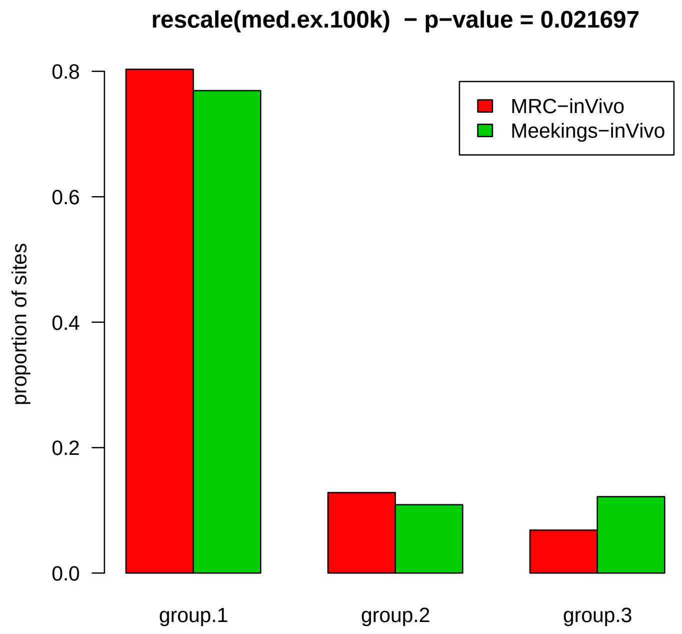

And here we count genes in the upper $1/16^{th}$:

```
Category limits

      lower category     upper
1 -0.150789  group.1 0.8053770
2  0.805377  group.2 0.9997078
```

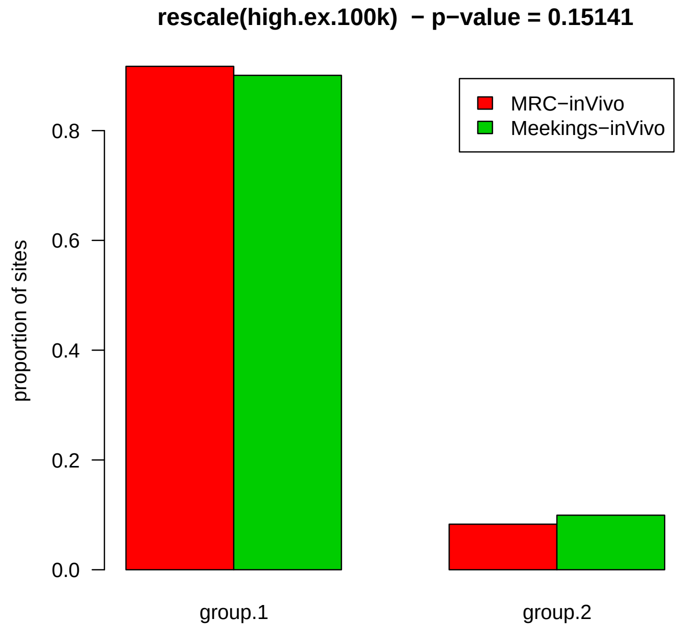

Here the effect of density of CpG islands is studied:

```
Category limits

       lower category     upper
1 -0.5829924  group.1 0.0371128
2  0.0371128  group.2 0.3696669
3  0.3696669  group.3 0.5824079
4  0.5824079  group.4 0.7857978
5  0.7857978  group.5 0.9997078
```

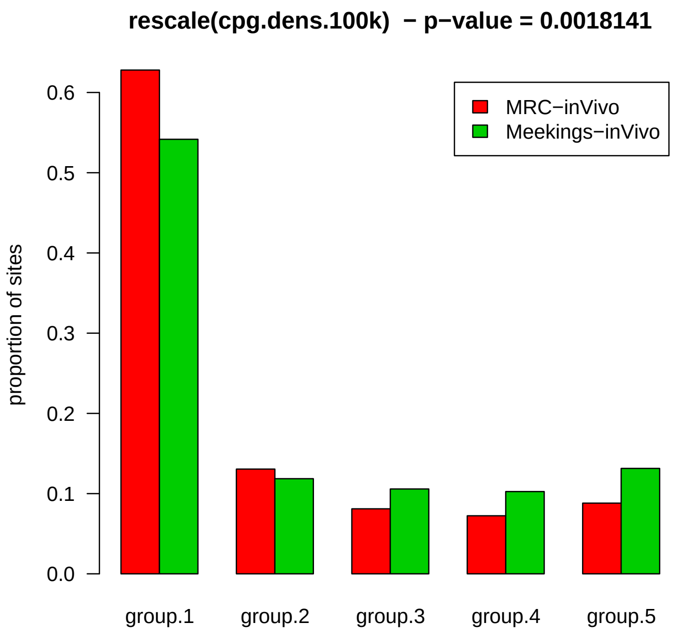

4.5 250 kiloBase Window

In the barplot that follows we examine the association of insertion sites with expression density in a 250 kilobase window surrounding each locus. First, we count just the number of genes on the represented on the chip.

```
 Category limits

        lower category       upper
1 -0.75862069  group.1 -0.39970777
2 -0.39970777  group.2 -0.26154296
3 -0.26154296  group.3  0.06896552
4  0.06896552  group.4  0.19783752
5  0.19783752  group.5  0.40385739
6  0.40385739  group.6  0.59964933
7  0.59964933  group.7  0.79544126
8  0.79544126  group.8  0.99970777
```

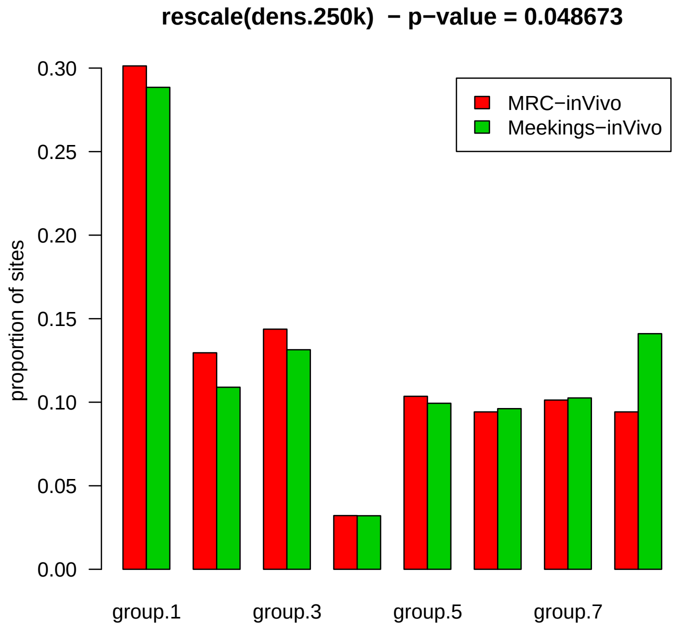

Here are the results for expression density. First, we count just genes that are in the upper half.

```
 Category limits

          lower category        upper
1 -0.5219170076  group.1 0.0005844535
2  0.0005844535  group.2 0.2650496786
3  0.2650496786  group.3 0.3991817650
4  0.3991817650  group.4 0.6005260082
5  0.6005260082  group.5 0.8009935710
6  0.8009935710  group.6 0.9997077732
```

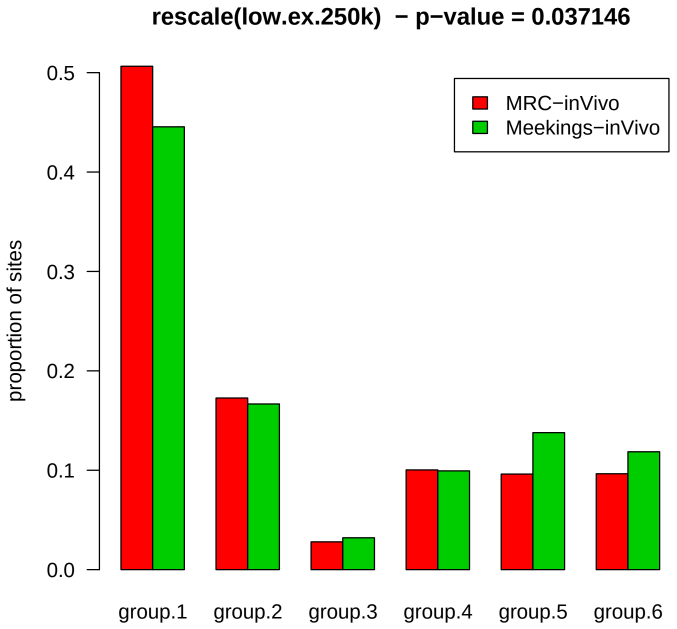

Now we count genes in the upper $1/8^{th}$:

```
Category limits

       lower category     upper
1 -0.3977206  group.1 0.3661601
2  0.3661601  group.2 0.5622443
3  0.5622443  group.3 0.7995324
4  0.7995324  group.4 0.9997078
```

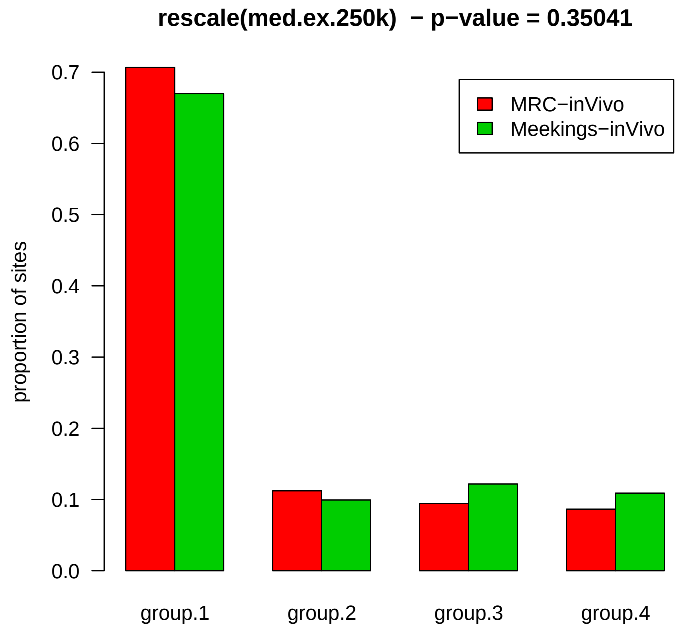

And here we count genes in the upper $1/16^{th}$:

```
Category limits

       lower  category     upper
1 -0.2849211  group.1 0.6072472
2  0.6072472  group.2 0.7665108
3  0.7665108  group.3 0.9997078
```

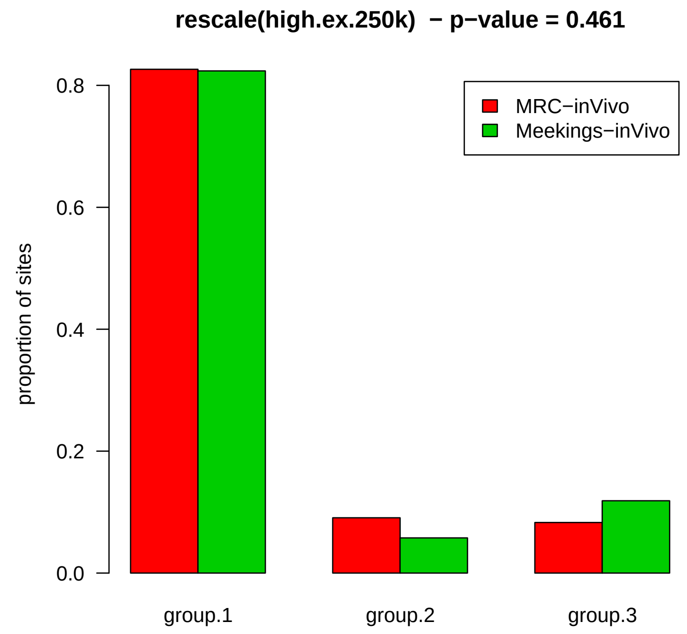

Here the effect of density of CpG islands is studied:

```
Category limits

        lower category       upper
1 -0.76914085  group.1 -0.39392168
2 -0.39392168  group.2 -0.13471654
3 -0.13471654  group.3  0.06779661
4  0.06779661  group.4  0.23436587
5  0.23436587  group.5  0.38427820
6  0.38427820  group.6  0.57510228
7  0.57510228  group.7  0.79602572
8  0.79602572  group.8  0.99970777
```

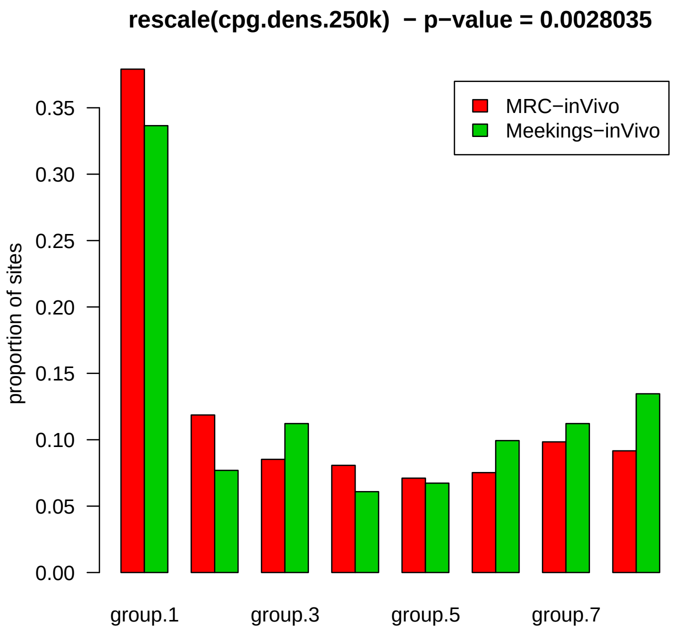

4.6 500 kilobase Window

Here are gene densities for the various gene calls

```
 Category limits

        lower category       upper
1 -0.75891292  group.1 -0.35563998
2 -0.35563998  group.2 -0.07568673
3 -0.07568673  group.3  0.12507306
4  0.12507306  group.4  0.41642314
5  0.41642314  group.5  0.62068966
6  0.62068966  group.6  0.81180596
7  0.81180596  group.7  0.99970777
```

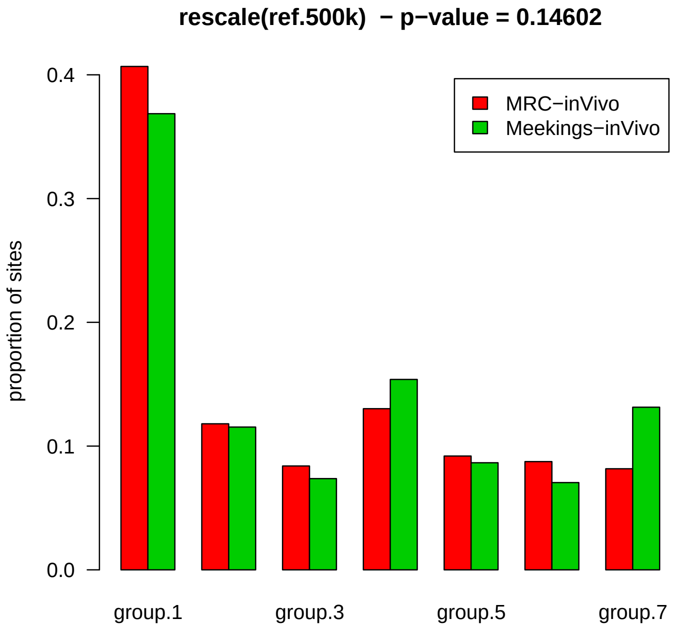

```
 Category limits

        lower category       upper
1 -0.81531268  group.1 -0.51315020
2 -0.51315020  group.2 -0.13179427
3 -0.13179427  group.3  0.01899474
4  0.01899474  group.4  0.15926359
5  0.15926359  group.5  0.37463472
6  0.37463472  group.6  0.59877265
7  0.59877265  group.7  0.80099357
8  0.80099357  group.8  0.99970777
```

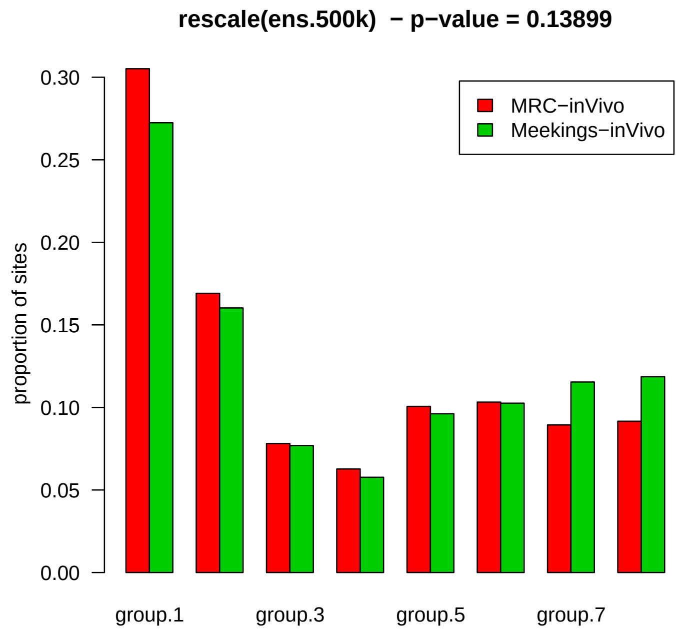

Category limits

	lower	category	upper
1	-0.96610169	group.1	-0.80187025
2	-0.80187025	group.2	-0.57334892
3	-0.57334892	group.3	-0.38924605
4	-0.38924605	group.4	-0.22618352
5	-0.22618352	group.5	0.02016365
6	0.02016365	group.6	0.21420222
7	0.21420222	group.7	0.38018703
8	0.38018703	group.8	0.59292811
9	0.59292811	group.9	0.79193454
10	0.79193454	group.10	0.99970777

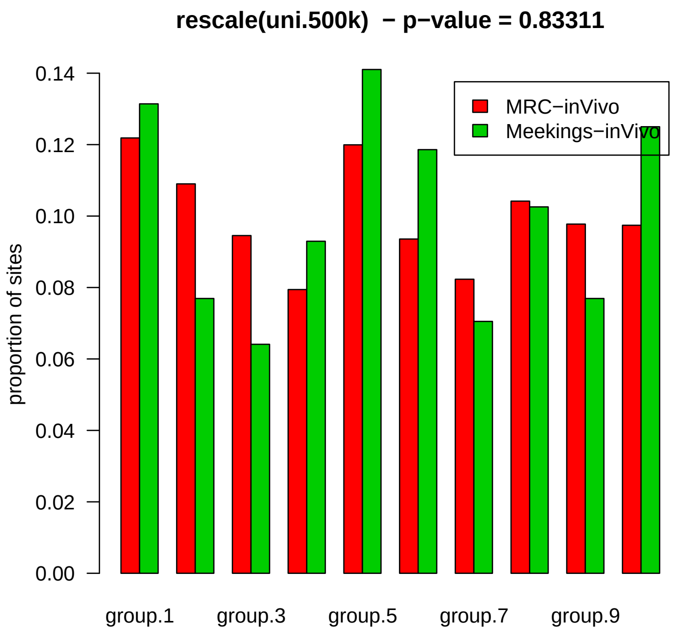

Category limits

	lower	category	upper
1	-0.99853887	group.1	-0.74663939
2	-0.74663939	group.2	-0.57860900
3	-0.57860900	group.3	-0.39596727
4	-0.39596727	group.4	-0.18994740
5	-0.18994740	group.5	0.02571596
6	0.02571596	group.6	0.22998247
7	0.22998247	group.7	0.39947399
8	0.39947399	group.8	0.63471654
9	0.63471654	group.9	0.78784337
10	0.78784337	group.10	0.99970777

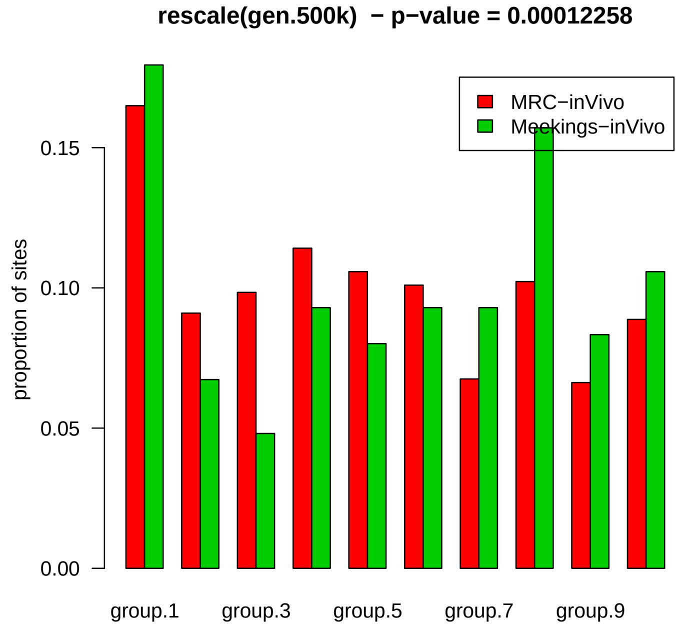

4.7 500 kiloBase Window

In the barplot that follows we examine the association of insertion sites with expression density in a 500 kilobase window surrounding each locus. First, we count just the number of genes on the represented on the chip.

```
 Category limits

         lower category        upper
1 -0.877849211  group.1 -0.612507306
2 -0.612507306  group.2 -0.391291642
3 -0.391291642  group.3 -0.186440678
4 -0.186440678  group.4  0.004675628
5  0.004675628  group.5  0.199941555
6  0.199941555  group.6  0.401811806
7  0.401811806  group.7  0.597019287
8  0.597019287  group.8  0.799532437
9  0.799532437  group.9  0.999707773
```

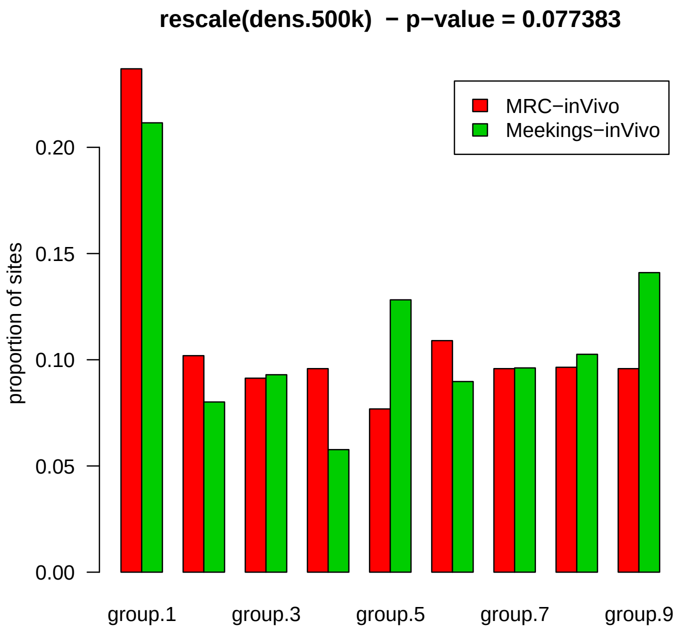

Here are the results for expression density. First, we count just genes that are in the upper half.

```
 Category limits

        lower category       upper
1 -0.66861485  group.1 -0.66861485
2 -0.66861485  group.2 -0.20192870
3 -0.20192870  group.3 -0.07685564
4 -0.07685564  group.4  0.20572764
5  0.20572764  group.5  0.40911748
6  0.40911748  group.6  0.60005845
7  0.60005845  group.7  0.79976622
8  0.79976622  group.8  0.99970777
```

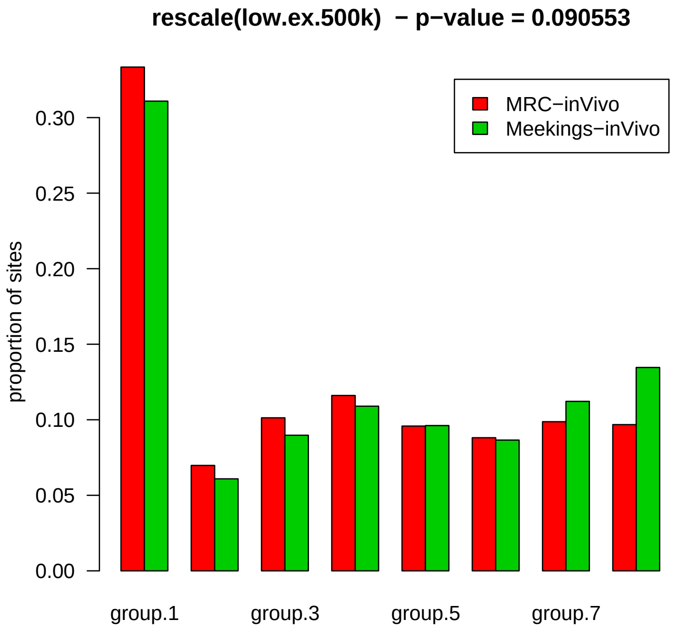

Now we count genes in the upper $1/8^{th}$:

```
Category limits

          lower category         upper
1 -0.5520163647  group.1 -0.0005844535
2 -0.0005844535  group.2  0.2530683811
3  0.2530683811  group.3  0.3998831093
4  0.3998831093  group.4  0.6002337814
5  0.6002337814  group.5  0.7995324372
6  0.7995324372  group.6  0.9997077732
```

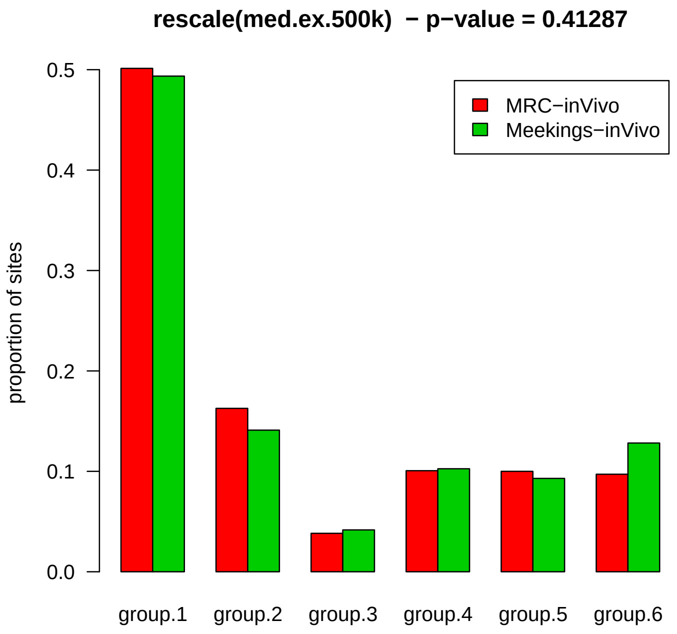

And here we count genes in the upper $1/16^{th}$:

```
Category limits

       lower category     upper
1 -0.4327878  group.1 0.1999416
2  0.1999416  group.2 0.4004968
3  0.4004968  group.3 0.5368206
4  0.5368206  group.4 0.8005552
5  0.8005552  group.5 0.9997078
```

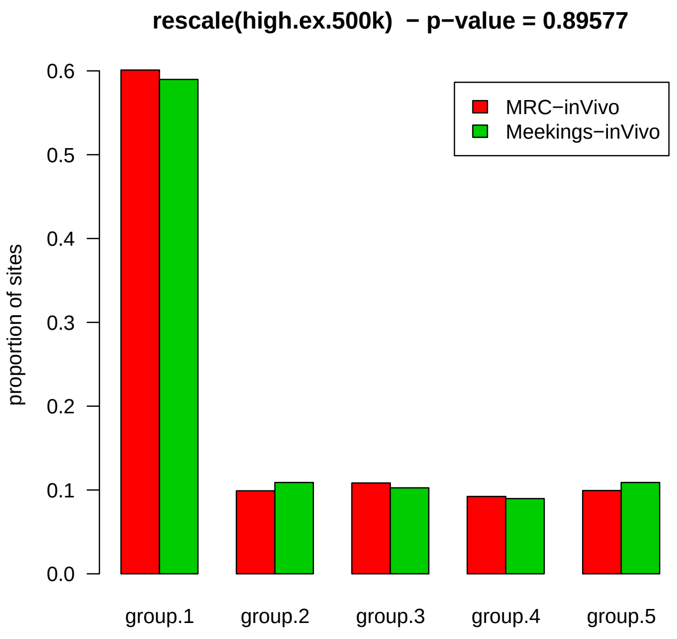

Here the effect of density of CpG islands is studied:

```
Category limits

        lower category       upper
1 -0.88749269  group.1 -0.68527177
2 -0.68527177  group.2 -0.33781414
3 -0.33781414  group.3 -0.19462303
4 -0.19462303  group.4  0.03857393
5  0.03857393  group.5  0.23202805
6  0.23202805  group.6  0.39479836
7  0.39479836  group.7  0.59672706
8  0.59672706  group.8  0.80508475
9  0.80508475  group.9  0.99970777
```

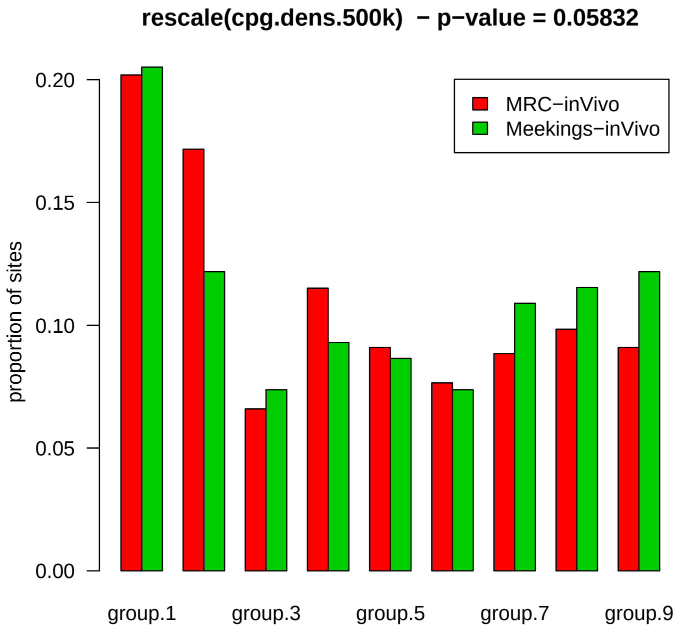

4.8 1 megabase Window

Here are gene densities for the various gene calls

```
 Category limits

        lower category       upper
1 -0.88544711  group.1 -0.66072472
2 -0.66072472  group.2 -0.46113384
3 -0.46113384  group.3 -0.17825833
4 -0.17825833  group.4 -0.04412624
5 -0.04412624  group.5  0.19316189
6  0.19316189  group.6  0.36645237
7  0.36645237  group.7  0.61542957
8  0.61542957  group.8  0.80420807
9  0.80420807  group.9  0.99970777
```

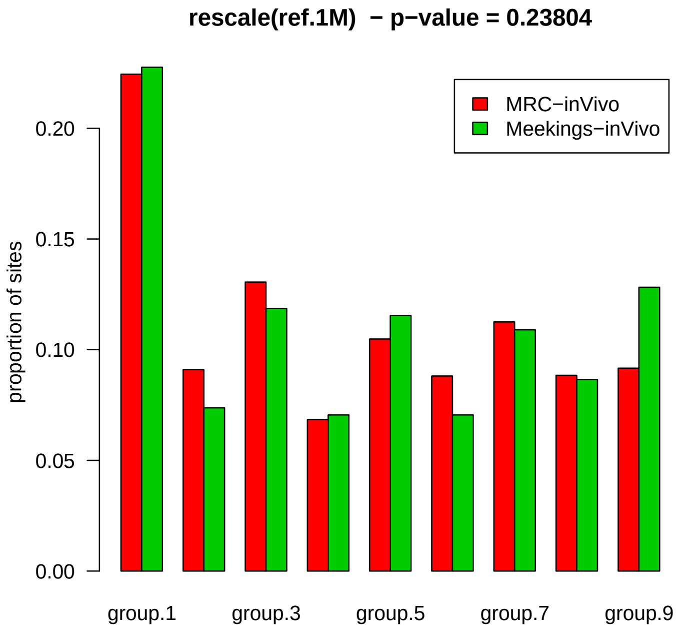

Category limits

	lower	category	upper
1	-0.92255991	group.1	-0.77586207
2	-0.77586207	group.2	-0.63354763
3	-0.63354763	group.3	-0.38924605
4	-0.38924605	group.4	-0.19082408
5	-0.19082408	group.5	-0.01578025
6	-0.01578025	group.6	0.20601987
7	0.20601987	group.7	0.40064290
8	0.40064290	group.8	0.60198714
9	0.60198714	group.9	0.79777908
10	0.79777908	group.10	0.99970777

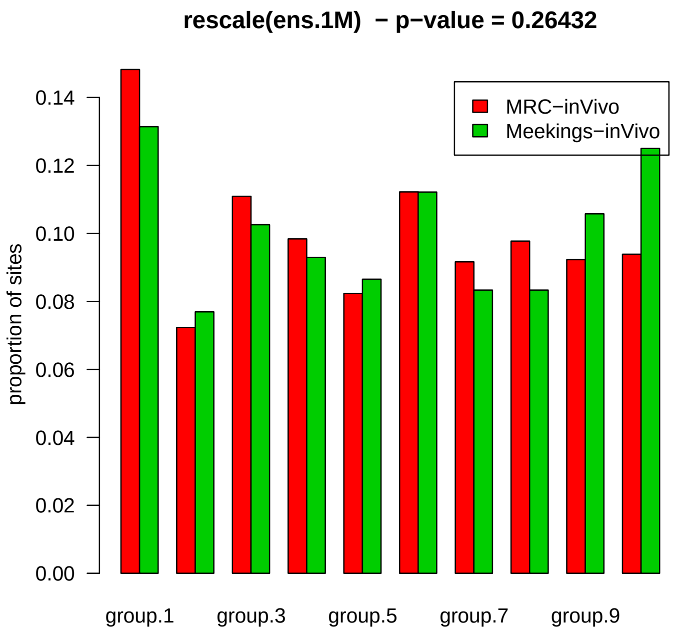

Category limits

	lower	category	upper
1	-0.9929866	group.1	-0.7986558
2	-0.7986558	group.2	-0.5999416
3	-0.5999416	group.3	-0.4038574
4	-0.4038574	group.4	-0.2098188
5	-0.2098188	group.5	0.0000000
6	0.0000000	group.6	0.2025132
7	0.2025132	group.7	0.3994740
8	0.3994740	group.8	0.6043250
9	0.6043250	group.9	0.8015780
10	0.8015780	group.10	0.9997078

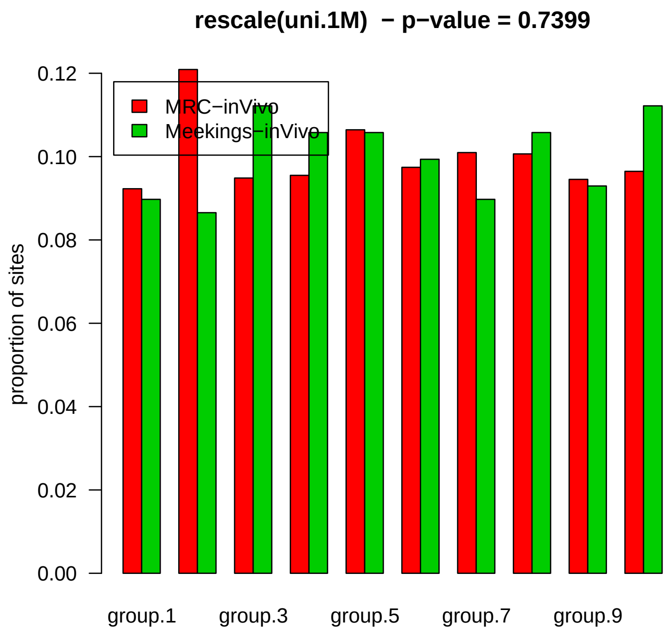

Category limits

	lower	category	upper
1	-0.99970777	group.1	-0.82437171
2	-0.82437171	group.2	-0.56428989
3	-0.56428989	group.3	-0.45616598
4	-0.45616598	group.4	-0.23056692
5	-0.23056692	group.5	0.01490357
6	0.01490357	group.6	0.23962595
7	0.23962595	group.7	0.43366452
8	0.43366452	group.8	0.58620690
9	0.58620690	group.9	0.79748685
10	0.79748685	group.10	0.99970777

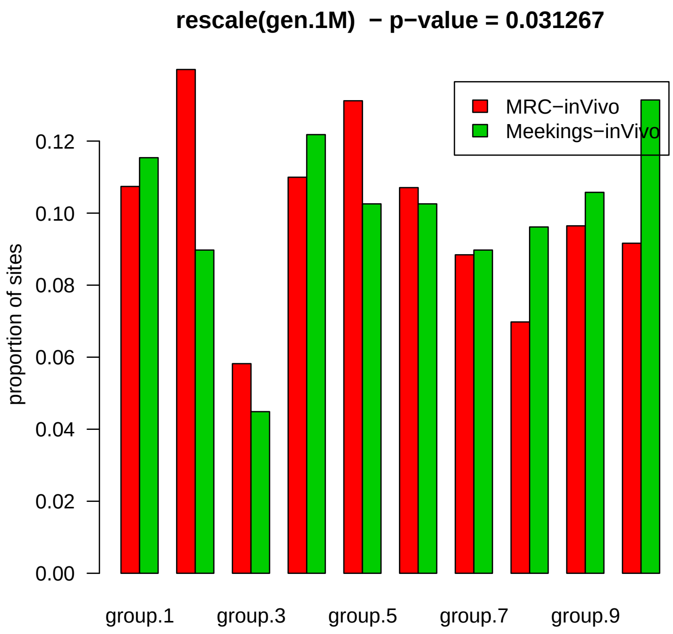

4.9 1 megaBase Window

In the barplot that follows we examine the association of insertion sites with expression density in a 1 megabase window surrounding each locus. First, we count just the number of genes on the represented on the chip.

```
 Category limits

        lower category      upper
1  -0.9590883  group.1 -0.8002922
2  -0.8002922  group.2 -0.5990649
3  -0.5990649  group.3 -0.3997662
4  -0.3997662  group.4 -0.2013442
5  -0.2013442  group.5  0.0000000
6   0.0000000  group.6  0.2019287
7   0.2019287  group.7  0.3997662
8   0.3997662  group.8  0.5987726
9   0.5987726  group.9  0.7997662
10  0.7997662 group.10  0.9997078
```

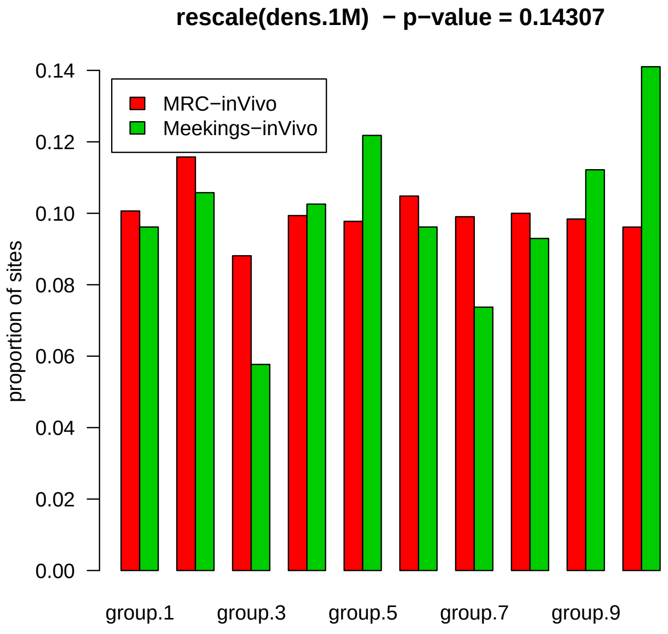

Here are the results for expression density. First, we count just genes that are in the upper half.

```
 Category limits

       lower category      upper
1 -0.8097604  group.1 -0.6002922
2 -0.6002922  group.2 -0.4140853
3 -0.4140853  group.3 -0.1914085
4 -0.1914085  group.4  0.0000000
5  0.0000000  group.5  0.1999416
6  0.1999416  group.6  0.3994740
7  0.3994740  group.7  0.5998247
8  0.5998247  group.8  0.7997370
9  0.7997370  group.9  0.9997078
```

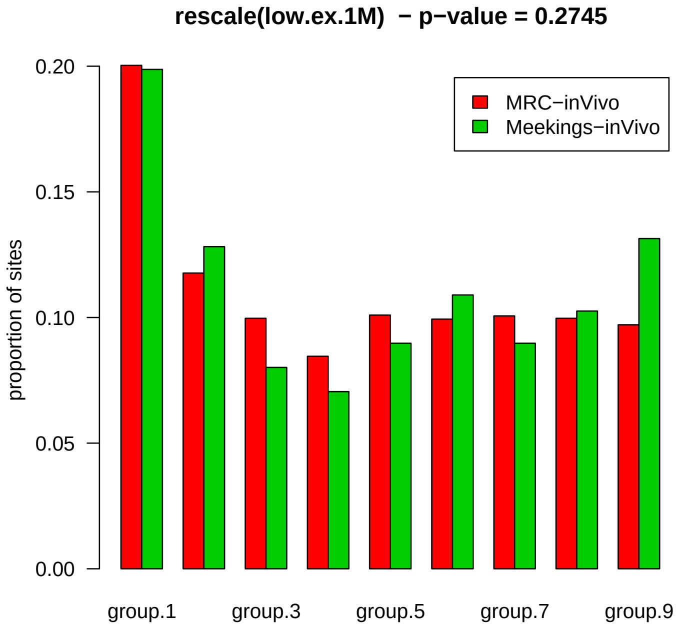

Now we count genes in the upper $1/8^{th}$:

```
Category limits

         lower category        upper
1 -0.716540035  group.1 -0.391291642
2 -0.391291642  group.2 -0.199941555
3 -0.199941555  group.3 -0.001461134
4 -0.001461134  group.4  0.194038574
5  0.194038574  group.5  0.401022794
6  0.401022794  group.6  0.600526008
7  0.600526008  group.7  0.800292227
8  0.800292227  group.8  0.999707773
```

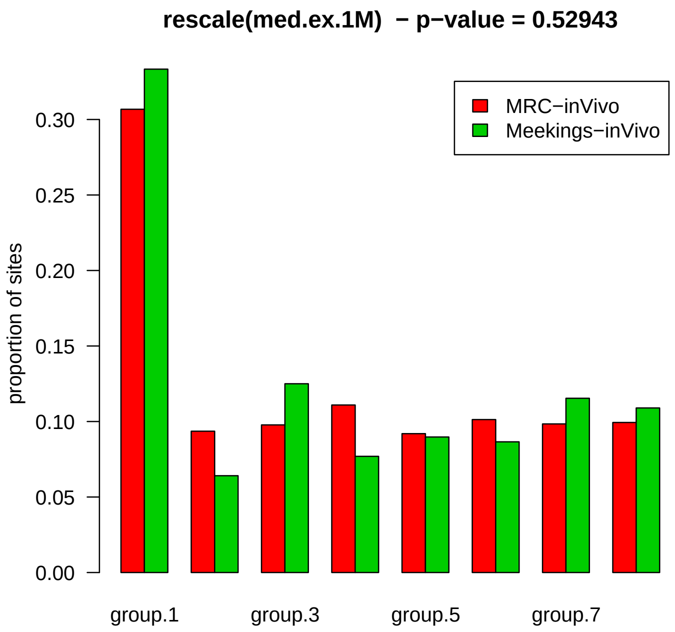

And here we count genes in the upper $1/16^{th}$:

```
Category limits

        lower category       upper
1 -0.61250731  group.1 -0.19935710
2 -0.19935710  group.2 -0.02220923
3 -0.02220923  group.3  0.21595558
4  0.21595558  group.4  0.39988311
5  0.39988311  group.5  0.59292811
6  0.59292811  group.6  0.79018118
7  0.79018118  group.7  0.99970777
```

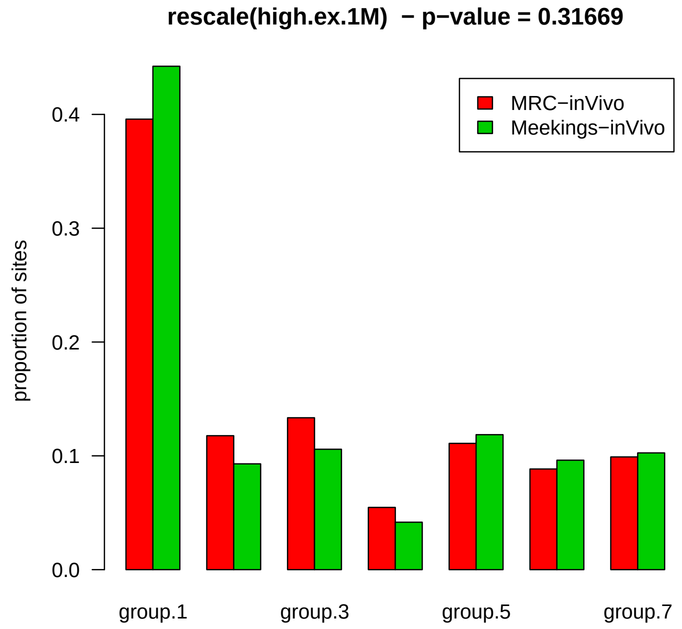

Here the effect of density of CpG islands is studied:

```
Category limits

        lower category       upper
1  -0.96668615  group.1 -0.79485681
2  -0.79485681  group.2 -0.62507306
3  -0.62507306  group.3 -0.37288136
4  -0.37288136  group.4 -0.19783752
5  -0.19783752  group.5  0.03419053
6   0.03419053  group.6  0.20017534
7   0.20017534  group.7  0.38398597
8   0.38398597  group.8  0.60695500
9   0.60695500  group.9  0.79953244
10  0.79953244 group.10  0.99970777
```

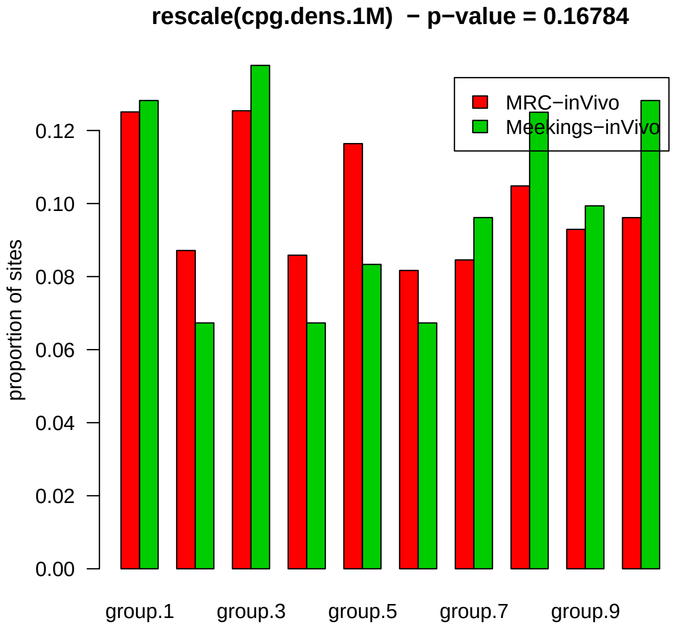

4.10 2 megabase Window

Here are gene densities for the various gene calls

```
 Category limits

          lower category       upper
1  -0.96580947  group.1 -0.77966102
2  -0.77966102  group.2 -0.60753945
3  -0.60753945  group.3 -0.43220339
4  -0.43220339  group.4 -0.16715371
5  -0.16715371  group.5 -0.01344243
6  -0.01344243  group.6  0.18965517
7   0.18965517  group.7  0.39713618
8   0.39713618  group.8  0.59438925
9   0.59438925  group.9  0.80157802
10  0.80157802 group.10  0.99970777
```

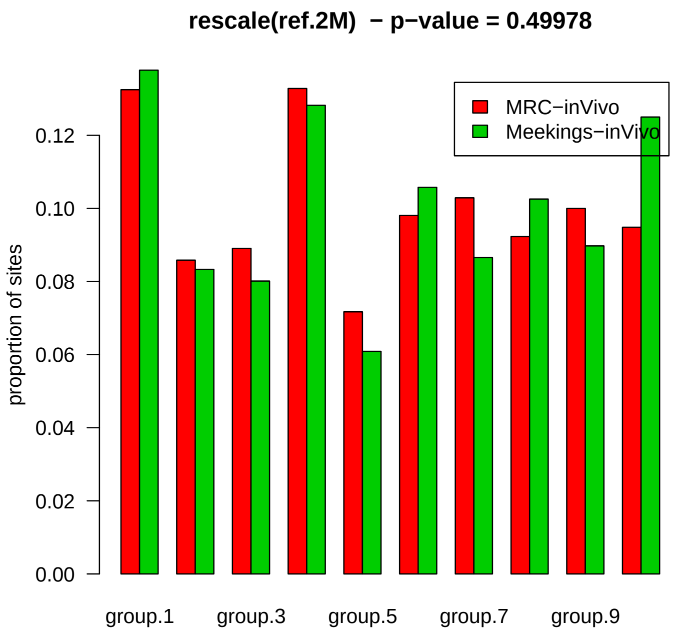

Category limits

	lower	category	upper
1	-0.9774985	group.1	-0.8202805
2	-0.8202805	group.2	-0.5768556
3	-0.5768556	group.3	-0.3994740
4	-0.3994740	group.4	-0.2124489
5	-0.2124489	group.5	0.0000000
6	0.0000000	group.6	0.1861485
7	0.1861485	group.7	0.4070719
8	0.4070719	group.8	0.5885447
9	0.5885447	group.9	0.7986558
10	0.7986558	group.10	0.9997078

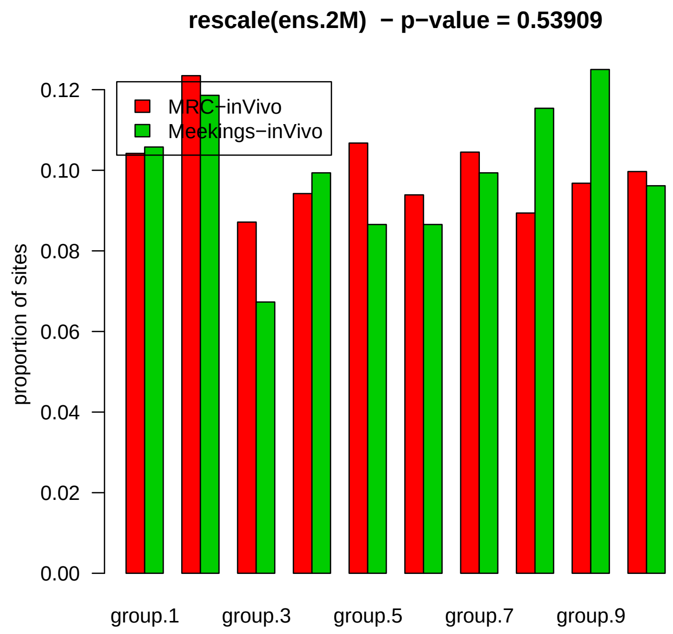

Category limits

	lower	category	upper
1	-0.9994155465	group.1	-0.7983635301
2	-0.7983635301	group.2	-0.5990648743
3	-0.5990648743	group.3	-0.4035651666
4	-0.4035651666	group.4	-0.2051431911
5	-0.2051431911	group.5	-0.0002922268
6	-0.0002922268	group.6	0.2036820573
7	0.2036820573	group.7	0.4035651666
8	0.4035651666	group.8	0.6014026885
9	0.6014026885	group.9	0.8012857978
10	0.8012857978	group.10	0.9997077732

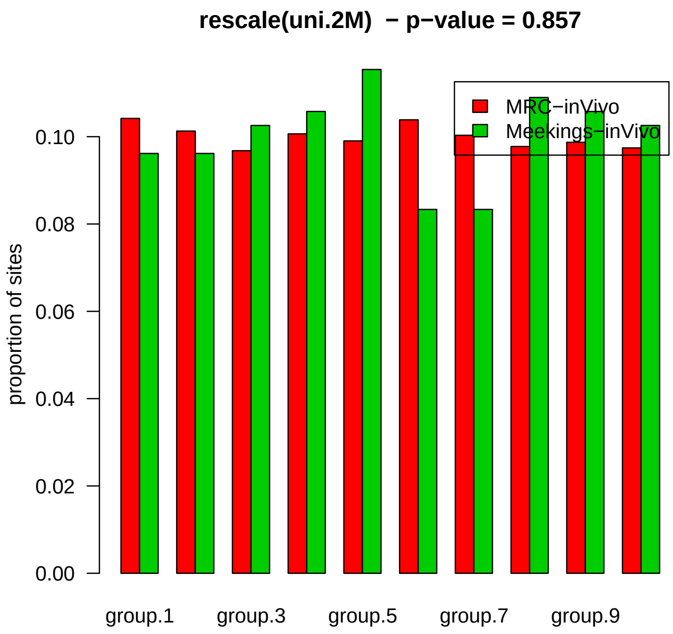

Category limits

	lower	category	upper
1	-0.99970777	group.1	-0.80683811
2	-0.80683811	group.2	-0.58591467
3	-0.58591467	group.3	-0.41057861
4	-0.41057861	group.4	-0.21887785
5	-0.21887785	group.5	-0.02425482
6	-0.02425482	group.6	0.18819404
7	0.18819404	group.7	0.42022209
8	0.42022209	group.8	0.60783168
9	0.60783168	group.9	0.80546464
10	0.80546464	group.10	0.99970777

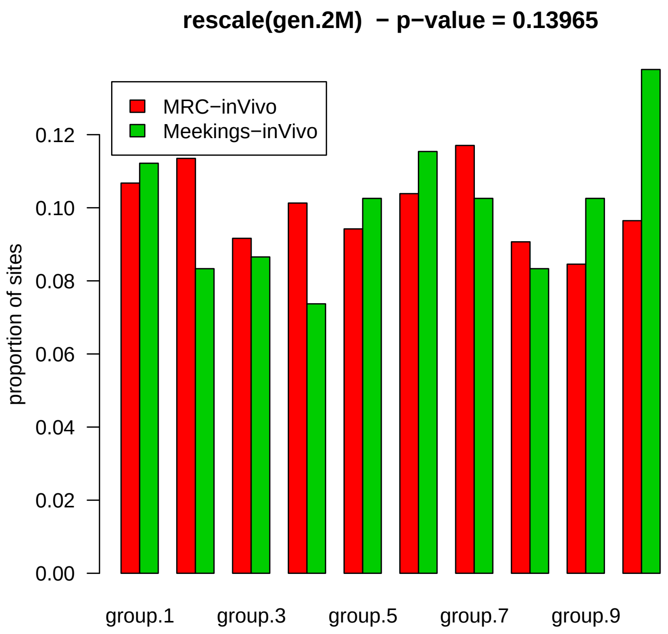

4.11 2 megaBase Window

In the barplot that follows we examine the association of insertion sites with expression density in a 2 megabase window surrounding each locus. First, we count just the number of genes on the represented on the chip.

```
 Category limits

           lower category        upper
1  -0.9929865576  group.1 -0.7995324372
2  -0.7995324372  group.2 -0.5998246639
3  -0.5998246639  group.3 -0.4000876680
4  -0.4000876680  group.4 -0.1999415546
5  -0.1999415546  group.5  0.0005844535
6   0.0005844535  group.6  0.2002922268
7   0.2002922268  group.7  0.3997077732
8   0.3997077732  group.8  0.5998246639
9   0.5998246639  group.9  0.7997662186
10  0.7997662186 group.10  0.9997077732
```

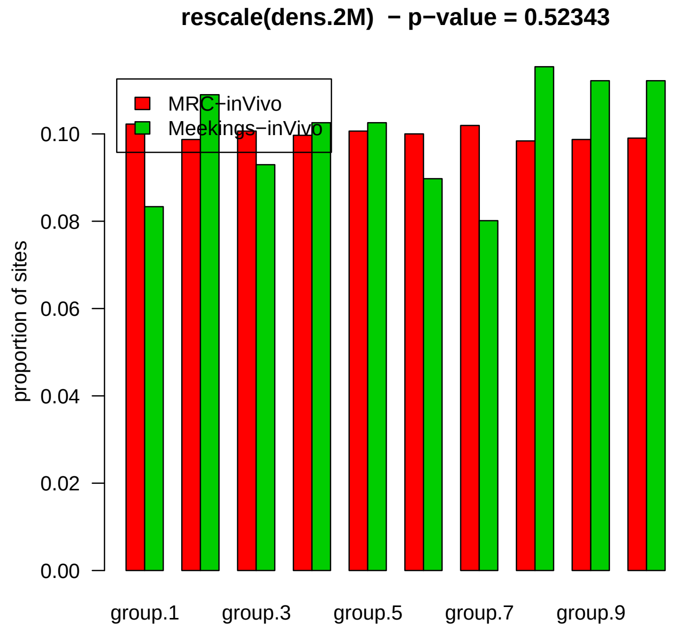

Here are the results for expression density. First, we count just genes that are in the upper half.

```
 Category limits

           lower category         upper
1  -0.9193454120  group.1 -0.8012857978
2  -0.8012857978  group.2 -0.6005260082
3  -0.6005260082  group.3 -0.4021040327
4  -0.4021040327  group.4 -0.2001753361
5  -0.2001753361  group.5 -0.0002922268
6  -0.0002922268  group.6  0.2001168907
7   0.2001168907  group.7  0.3998831093
8   0.3998831093  group.8  0.5998246639
9   0.5998246639  group.9  0.7997662186
10  0.7997662186 group.10  0.9997077732
```

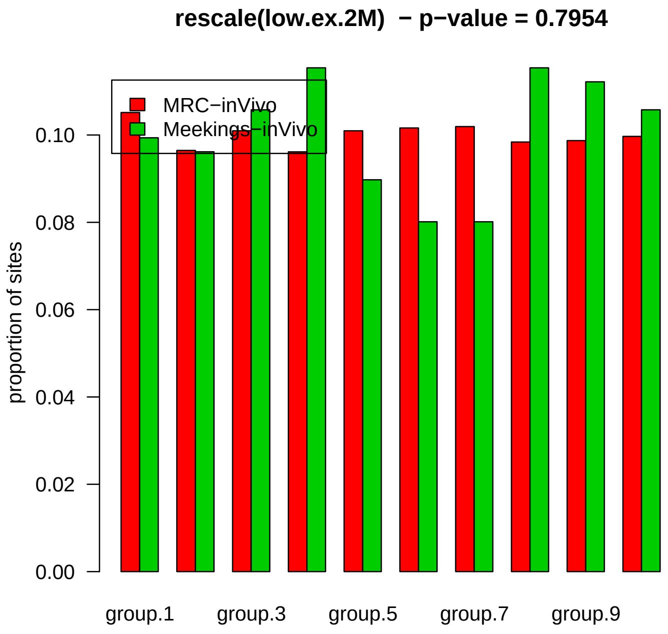

Now we count genes in the upper $1/8^{th}$:

```
Category limits

       lower category      upper
1 -0.8664524  group.1 -0.5990649
2 -0.5990649  group.2 -0.3994740
3 -0.3994740  group.3 -0.1998831
4 -0.1998831  group.4  0.0000000
5  0.0000000  group.5  0.1999416
6  0.1999416  group.6  0.3997662
7  0.3997662  group.7  0.6000584
8  0.6000584  group.8  0.7997370
9  0.7997370  group.9  0.9997078
```

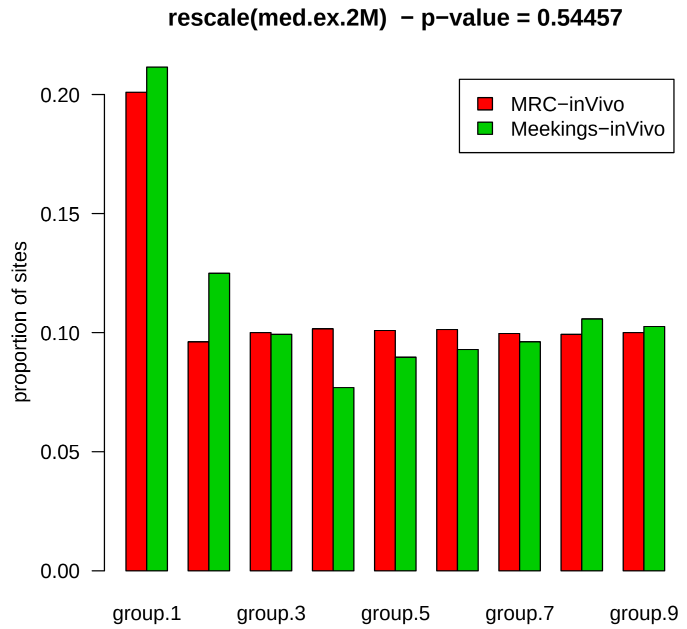

And here we count genes in the upper $1/16^{th}$:

```
Category limits

         lower  category        upper
1 -0.793395675  group.1 -0.433956750
2 -0.433956750  group.2 -0.228521333
3 -0.228521333  group.3  0.002630041
4  0.002630041  group.4  0.200116891
5  0.200116891  group.5  0.400642899
6  0.400642899  group.6  0.599941555
7  0.599941555  group.7  0.798071303
8  0.798071303  group.8  0.999707773
```

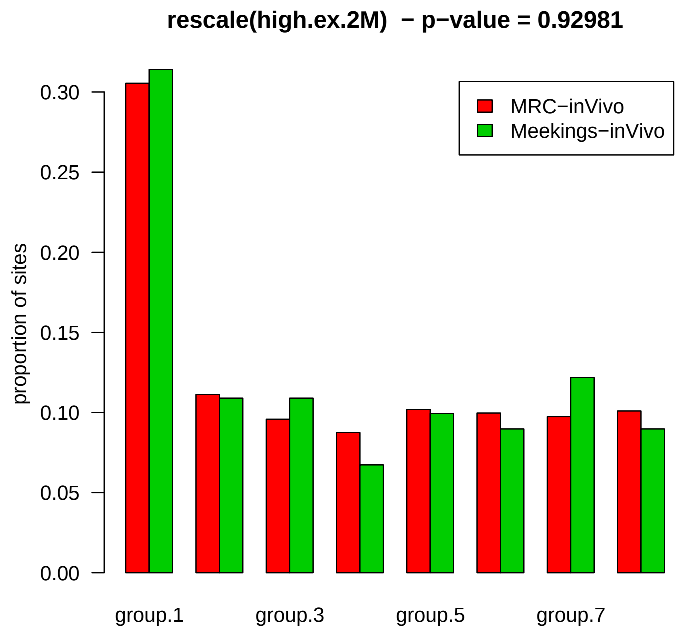

Here the effect of density of CpG islands is studied:

```
Category limits

          lower category       upper
1   -0.99649328  group.1 -0.79514904
2   -0.79514904  group.2 -0.61572180
3   -0.61572180  group.3 -0.40882525
4   -0.40882525  group.4 -0.21741672
5   -0.21741672  group.5 -0.01431911
6   -0.01431911  group.6  0.20017534
7    0.20017534  group.7  0.39392168
8    0.39392168  group.8  0.60081823
9    0.60081823  group.9  0.80040912
10   0.80040912 group.10  0.99970777
```

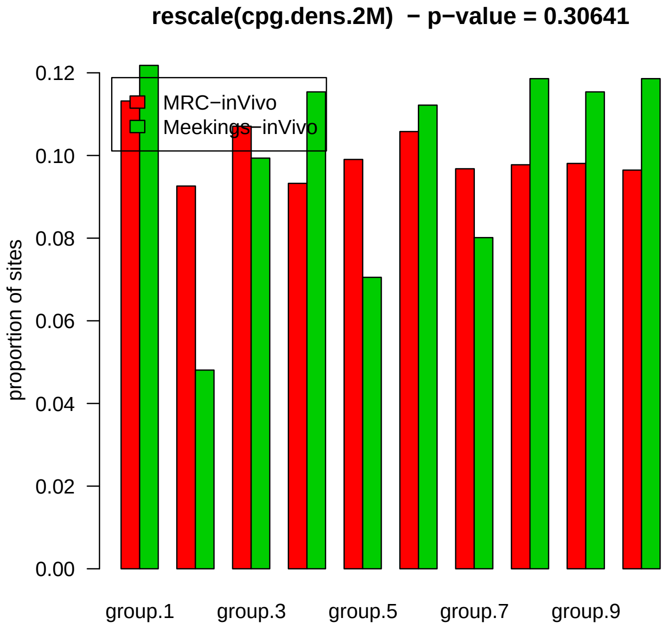

4.12 4 megaBase Window

In the barplot that follows we examine the association of insertion sites with expression density in a 4 megabase window surrounding each locus. First, we count just the number of genes on the represented on the chip.

```
 Category limits

        lower category      upper
1  -0.9994155  group.1 -0.7995324
2  -0.7995324  group.2 -0.5996493
3  -0.5996493  group.3 -0.3998831
4  -0.3998831  group.4 -0.1992987
5  -0.1992987  group.5  0.0000000
6   0.0000000  group.6  0.1999416
7   0.1999416  group.7  0.3998831
8   0.3998831  group.8  0.5998247
9   0.5998247  group.9  0.7997662
10  0.7997662 group.10  0.9997078
```

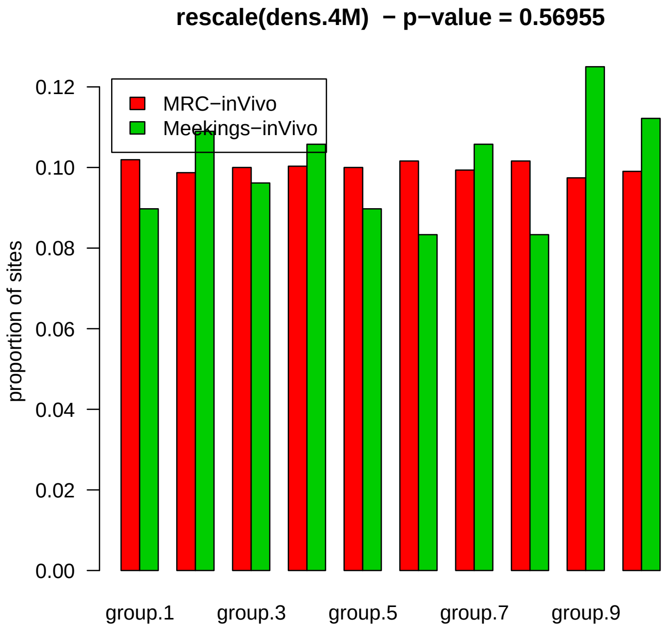

Here are the results for expression density. First, we count just genes that are in the upper half.

```
 Category limits

           lower category         upper
1  -0.9795441262  group.1 -0.8000292227
2  -0.8000292227  group.2 -0.5996493279
3  -0.5996493279  group.3 -0.3997954413
4  -0.3997954413  group.4 -0.1998246639
5  -0.1998246639  group.5 -0.0002922268
6  -0.0002922268  group.6  0.1999415546
7   0.1999415546  group.7  0.3998831093
8   0.3998831093  group.8  0.5998246639
9   0.5998246639  group.9  0.7997662186
10  0.7997662186 group.10  0.9997077732
```

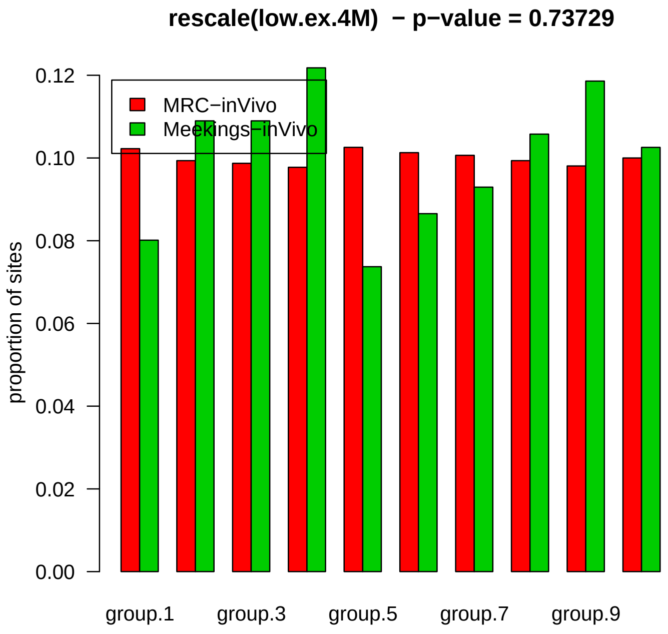

Now we count genes in the upper $1/8^{th}$:

```
Category limits

        lower  category      upper
1  -0.9605494   group.1 -0.7995324
2  -0.7995324   group.2 -0.5997078
3  -0.5997078   group.3 -0.3994740
4  -0.3994740   group.4 -0.2001753
5  -0.2001753   group.5  0.0000000
6   0.0000000   group.6  0.1995909
7   0.1995909   group.7  0.3997662
8   0.3997662   group.8  0.5999416
9   0.5999416   group.9  0.8000292
10  0.8000292  group.10  0.9997078
```

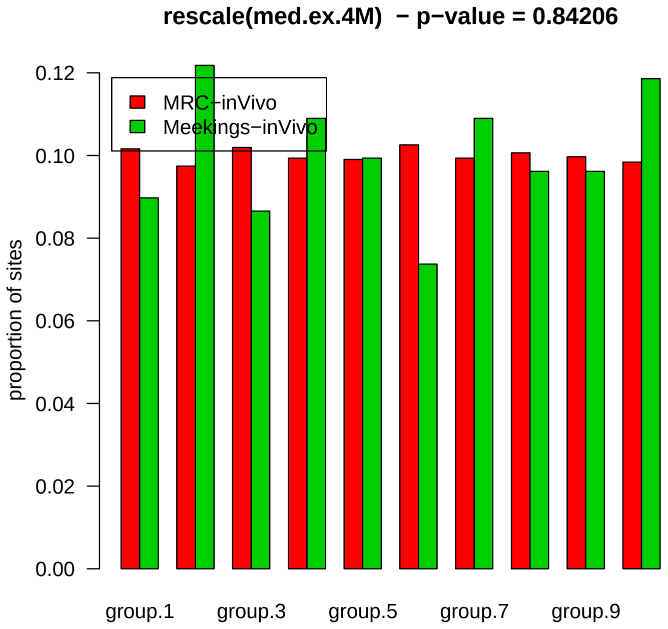

And here we count genes in the upper $1/16^{th}$:

```
Category limits

        lower category      upper
1  -0.9292811  group.1 -0.7863822
2  -0.7863822  group.2 -0.6022794
3  -0.6022794  group.3 -0.3804793
4  -0.3804793  group.4 -0.1999416
5  -0.1999416  group.5  0.0000000
6   0.0000000  group.6  0.2004676
7   0.2004676  group.7  0.3998831
8   0.3998831  group.8  0.5998247
9   0.5998247  group.9  0.7997662
10  0.7997662 group.10  0.9997078
```

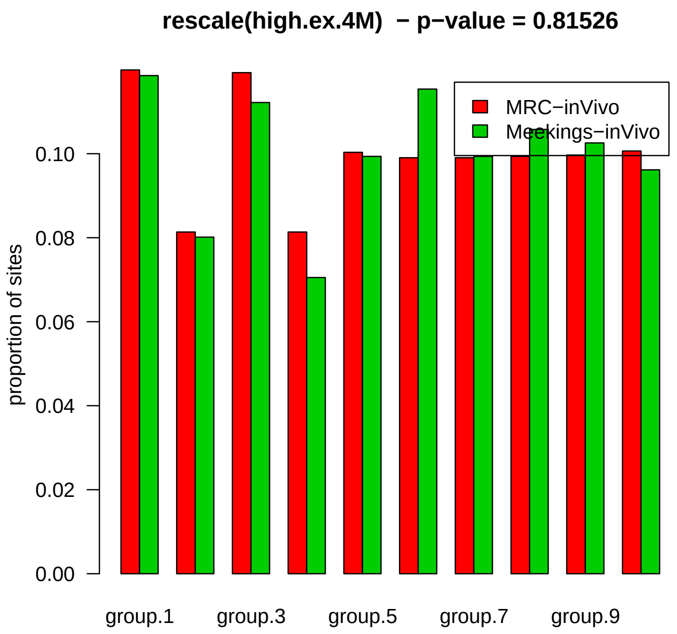

Here the effect of density of CpG islands is studied:

```
Category limits

           lower  category         upper
1   -0.9997077732  group.1 -0.8067212157
2   -0.8067212157  group.2 -0.6028638223
3   -0.6028638223  group.3 -0.3886616014
4   -0.3886616014  group.4 -0.1946230275
5   -0.1946230275  group.5  0.0002922268
6    0.0002922268  group.6  0.2019286967
7    0.2019286967  group.7  0.3974284044
8    0.3974284044  group.8  0.5967270602
9    0.5967270602  group.9  0.8009935710
10   0.8009935710 group.10  0.9997077732
```

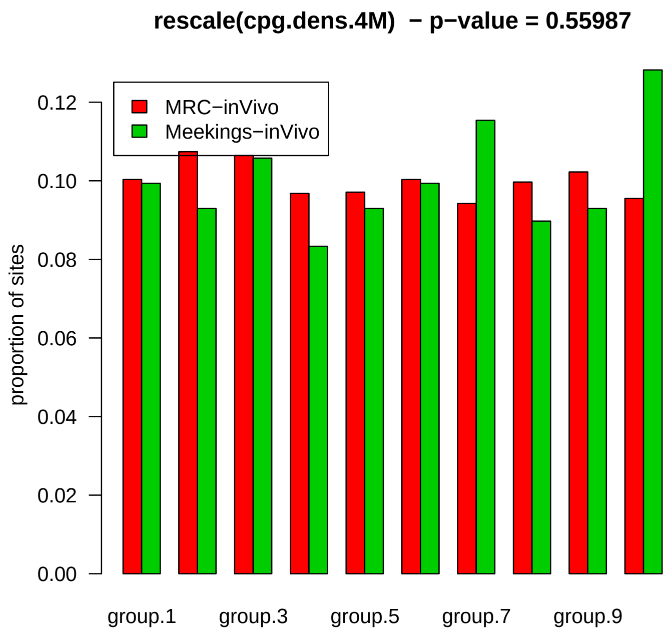

4.13 8 megaBase Window

In the barplot that follows we examine the association of insertion sites with expression density in a 8 megabase window surrounding each locus. First, we count just the number of genes on the represented on the chip.

```
 Category limits

        lower category      upper
1  -0.9997078  group.1 -0.7995324
2  -0.7995324  group.2 -0.5996493
3  -0.5996493  group.3 -0.3998831
4  -0.3998831  group.4 -0.1999416
5  -0.1999416  group.5  0.0000000
6   0.0000000  group.6  0.1999416
7   0.1999416  group.7  0.4000877
8   0.4000877  group.8  0.5998247
9   0.5998247  group.9  0.7997662
10  0.7997662 group.10  0.9997078
```

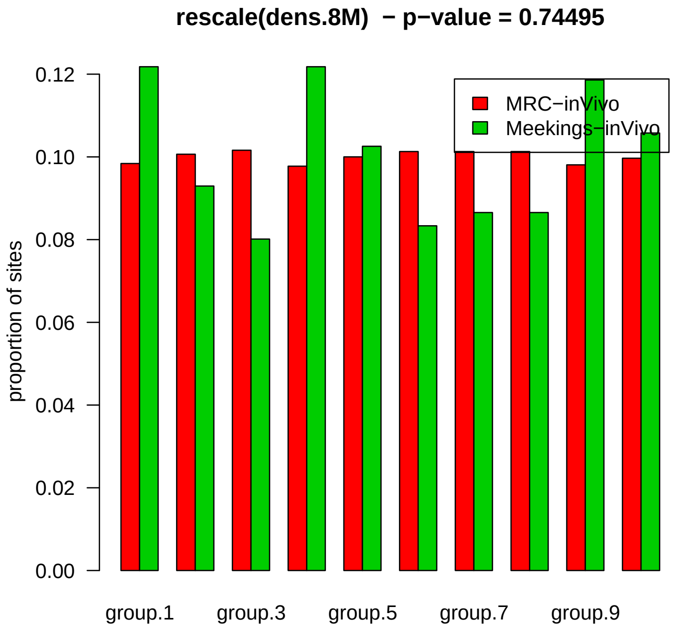

Here are the results for expression density. First, we count just genes that are in the upper half.

```
 Category limits

        lower category      upper
1  -0.9985389  group.1 -0.7995324
2  -0.7995324  group.2 -0.6000000
3  -0.6000000  group.3 -0.3998831
4  -0.3998831  group.4 -0.1999416
5  -0.1999416  group.5  0.0000000
6   0.0000000  group.6  0.2002922
7   0.2002922  group.7  0.3998831
8   0.3998831  group.8  0.5998247
9   0.5998247  group.9  0.7997370
10  0.7997370 group.10  0.9997078
```

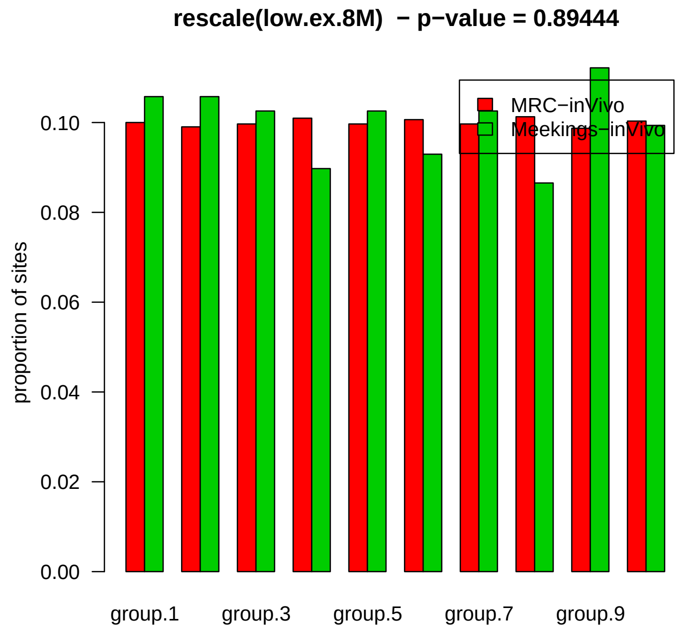

Now we count genes in the upper $1/8^{th}$:

```
Category limits

        lower  category      upper
1  -0.9950321   group.1 -0.8002922
2  -0.8002922   group.2 -0.5998247
3  -0.5998247   group.3 -0.3998831
4  -0.3998831   group.4 -0.1998831
5  -0.1998831   group.5  0.0000000
6   0.0000000   group.6  0.1999416
7   0.1999416   group.7  0.4000877
8   0.4000877   group.8  0.5998247
9   0.5998247   group.9  0.7997662
10  0.7997662  group.10  0.9997078
```

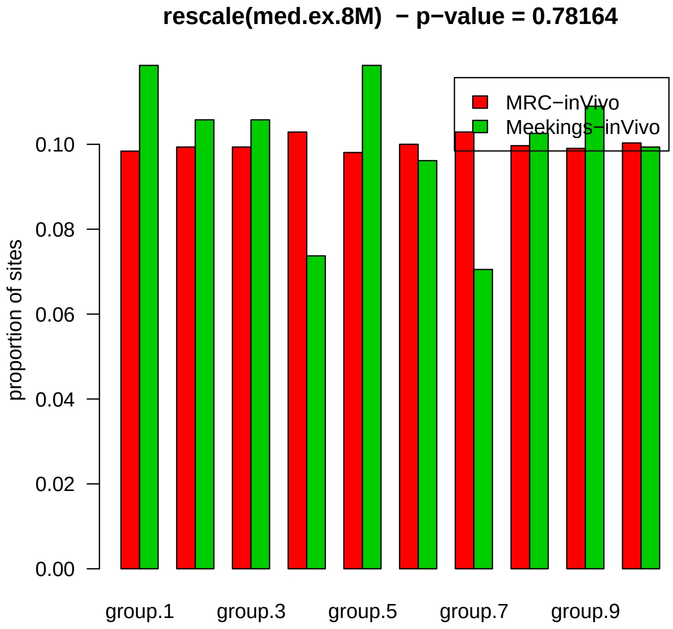

And here we count genes in the upper $1/16^{th}$:

```
Category limits

         lower category      upper
1  -0.9845120  group.1 -0.8013442
2  -0.8013442  group.2 -0.5998247
3  -0.5998247  group.3 -0.3998831
4  -0.3998831  group.4 -0.2001753
5  -0.2001753  group.5  0.0000000
6   0.0000000  group.6  0.1999416
7   0.1999416  group.7  0.4003507
8   0.4003507  group.8  0.5998247
9   0.5998247  group.9  0.8000292
10  0.8000292 group.10  0.9988311
```

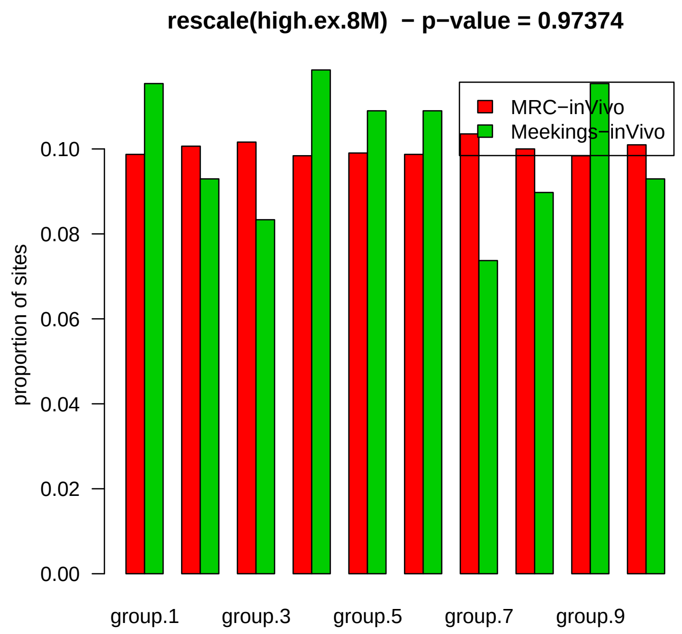

Here the effect of density of CpG islands is studied:

```
Category limits

          lower category        upper
1  -0.999707773  group.1 -0.794564582
2  -0.794564582  group.2 -0.596434833
3  -0.596434833  group.3 -0.395382817
4  -0.395382817  group.4 -0.203682057
5  -0.203682057  group.5  0.001168907
6   0.001168907  group.6  0.196084161
7   0.196084161  group.7  0.399766219
8   0.399766219  group.8  0.599357101
9   0.599357101  group.9  0.799824664
10  0.799824664 group.10  0.999707773
```

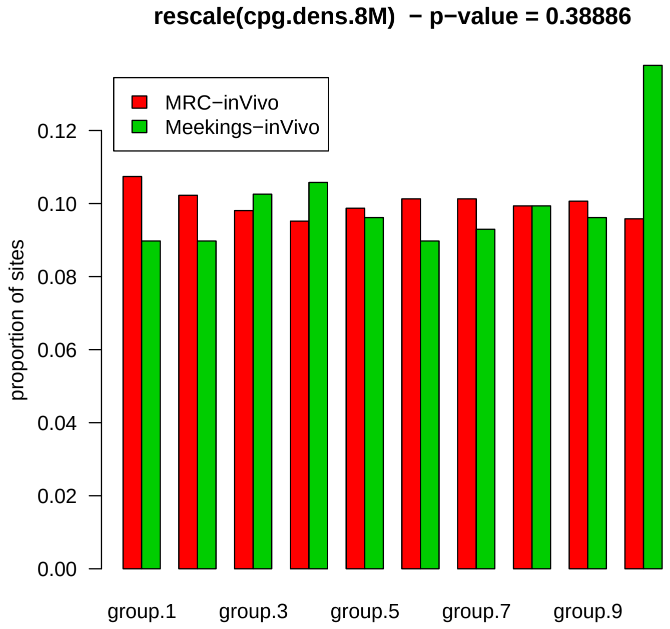

4.14 16 megaBase Window

In the barplot that follows we examine the association of insertion sites with expression density in a 16 megabase window surrounding each locus. First, we count just the number of genes on the represented on the chip.

```
 Category limits

        lower category      upper
1  -0.9997078  group.1 -0.7997370
2  -0.7997370  group.2 -0.5998247
3  -0.5998247  group.3 -0.3994740
4  -0.3994740  group.4 -0.1998247
5  -0.1998247  group.5  0.0000000
6   0.0000000  group.6  0.1999416
7   0.1999416  group.7  0.3998831
8   0.3998831  group.8  0.5998247
9   0.5998247  group.9  0.7997662
10  0.7997662 group.10  0.9997078
```

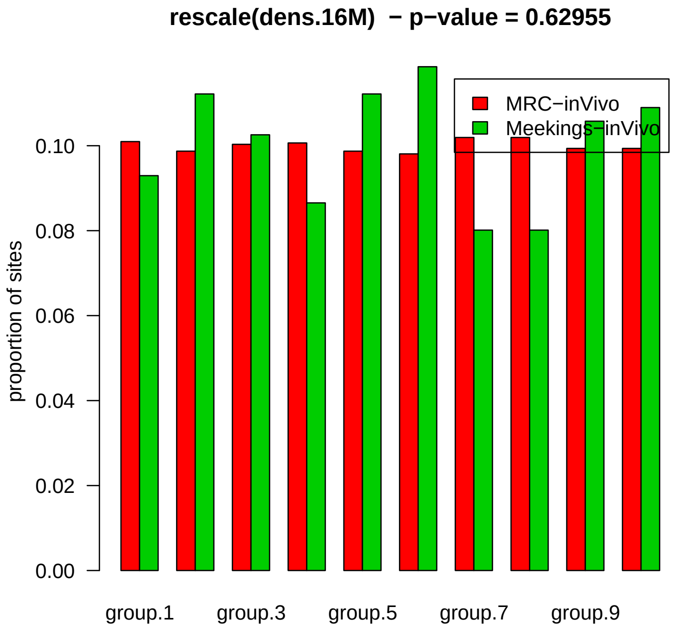

Here are the results for expression density. First, we count just genes that are in the upper half.

```
 Category limits

           lower category         upper
1  -0.9994155465  group.1 -0.7997662186
2  -0.7997662186  group.2 -0.5993571011
3  -0.5993571011  group.3 -0.4007013442
4  -0.4007013442  group.4 -0.1998831093
5  -0.1998831093  group.5 -0.0001461134
6  -0.0001461134  group.6  0.1998246639
7   0.1998246639  group.7  0.3997662186
8   0.3997662186  group.8  0.6000584454
9   0.6000584454  group.9  0.8000292227
10  0.8000292227 group.10  0.9997077732
```

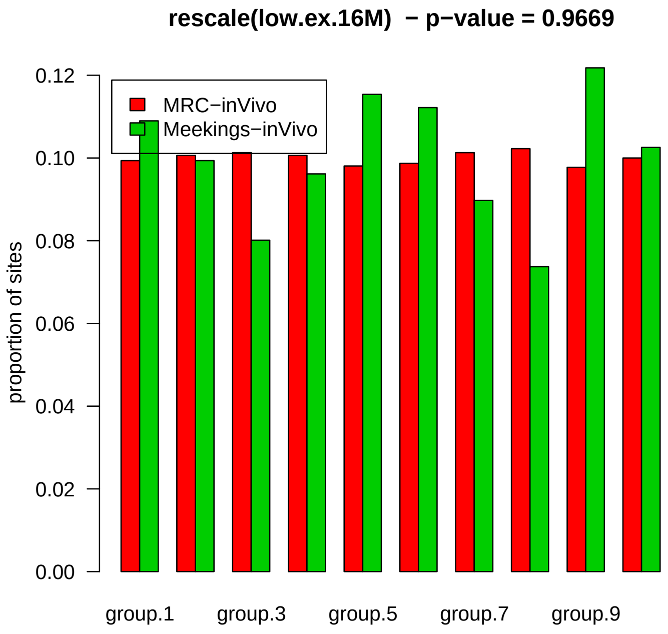

Now we count genes in the upper $1/8^{th}$:

```
Category limits

         lower category      upper
1   -0.9997078  group.1 -0.8005552
2   -0.8005552  group.2 -0.5996493
3   -0.5996493  group.3 -0.3998831
4   -0.3998831  group.4 -0.2001753
5   -0.2001753  group.5  0.0000000
6    0.0000000  group.6  0.1999416
7    0.1999416  group.7  0.3997954
8    0.3997954  group.8  0.6000584
9    0.6000584  group.9  0.7997662
10   0.7997662 group.10  0.9997078
```

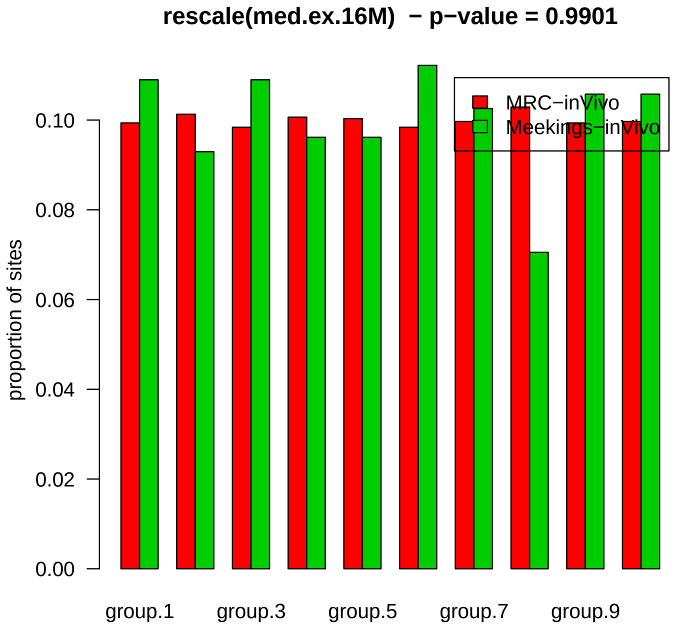

And here we count genes in the upper $1/16^{th}$:

```
Category limits

           lower category         upper
1  -0.9991233197  group.1 -0.7986557569
2  -0.7986557569  group.2 -0.5997077732
3  -0.5997077732  group.3 -0.3997662186
4  -0.3997662186  group.4 -0.1998831093
5  -0.1998831093  group.5 -0.0001461134
6  -0.0001461134  group.6  0.1997077732
7   0.1997077732  group.7  0.3994739918
8   0.3994739918  group.8  0.6000000000
9   0.6000000000  group.9  0.7997077732
10  0.7997077732 group.10  0.9997077732
```

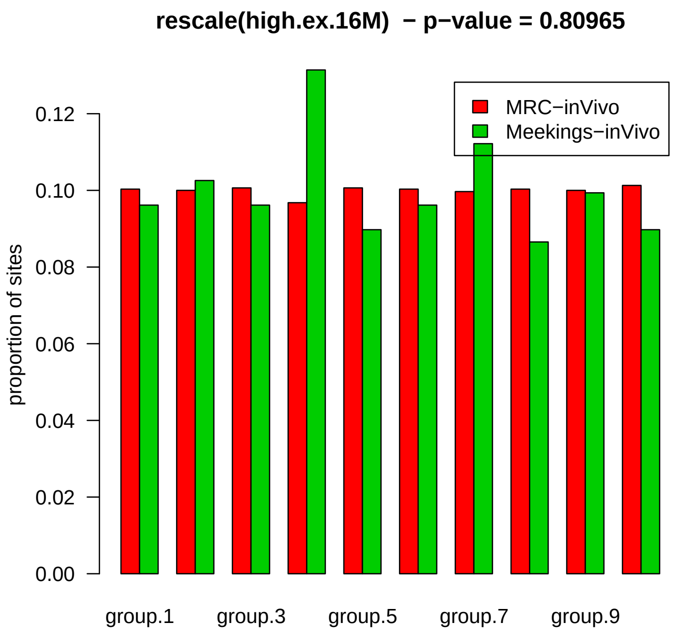

Here the effect of density of CpG islands is studied:

```
Category limits

          lower category        upper
1  -0.999123320  group.1 -0.796317943
2  -0.796317943  group.2 -0.597311514
3  -0.597311514  group.3 -0.395967271
4  -0.395967271  group.4 -0.201052016
5  -0.201052016  group.5 -0.003214494
6  -0.003214494  group.6  0.199006429
7   0.199006429  group.7  0.399883109
8   0.399883109  group.8  0.600058445
9   0.600058445  group.9  0.799736996
10  0.799736996 group.10  0.999707773
```

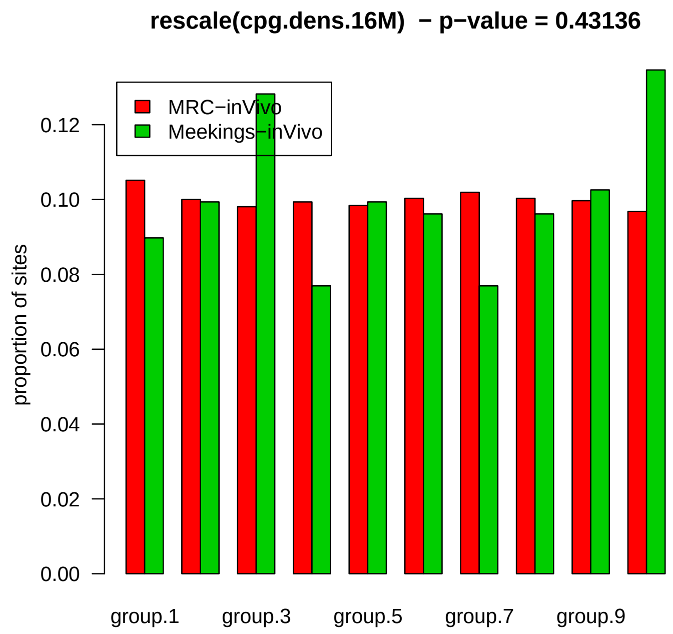

4.15 32 megaBase Window

In the barplot that follows we examine the association of insertion sites with expression density in a 32 megabase window surrounding each locus. First, we count just the number of genes on the represented on the chip.

```
 Category limits

        lower category      upper
1  -0.9994155  group.1 -0.7995324
2  -0.7995324  group.2 -0.5996493
3  -0.5996493  group.3 -0.3998831
4  -0.3998831  group.4 -0.1999416
5  -0.1999416  group.5  0.0000000
6   0.0000000  group.6  0.1999416
7   0.1999416  group.7  0.3998831
8   0.3998831  group.8  0.5998247
9   0.5998247  group.9  0.7997662
10  0.7997662 group.10  0.9997078
```

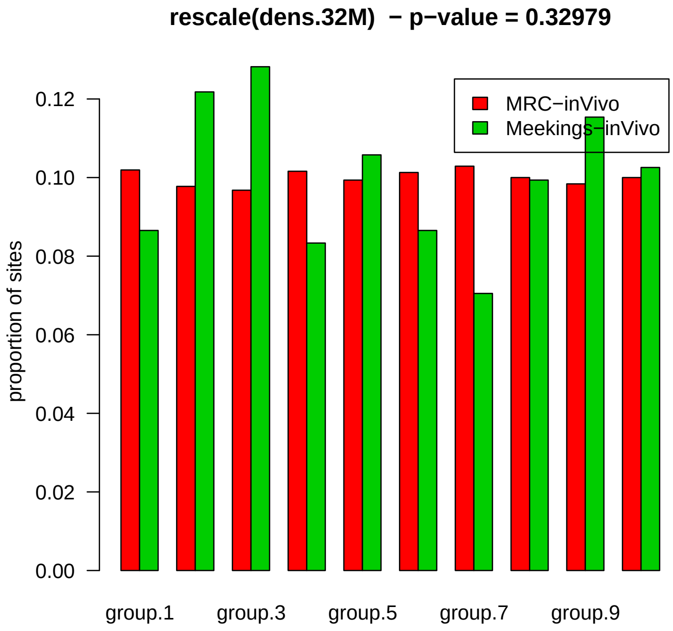

Here are the results for expression density. First, we count just genes that are in the upper half.

```
 Category limits

         lower category      upper
1  -0.9994155  group.1 -0.7995324
2  -0.7995324  group.2 -0.5998247
3  -0.5998247  group.3 -0.3998831
4  -0.3998831  group.4 -0.1998831
5  -0.1998831  group.5  0.0000000
6   0.0000000  group.6  0.1998831
7   0.1998831  group.7  0.3998831
8   0.3998831  group.8  0.5998247
9   0.5998247  group.9  0.7997662
10  0.7997662 group.10  0.9997078
```

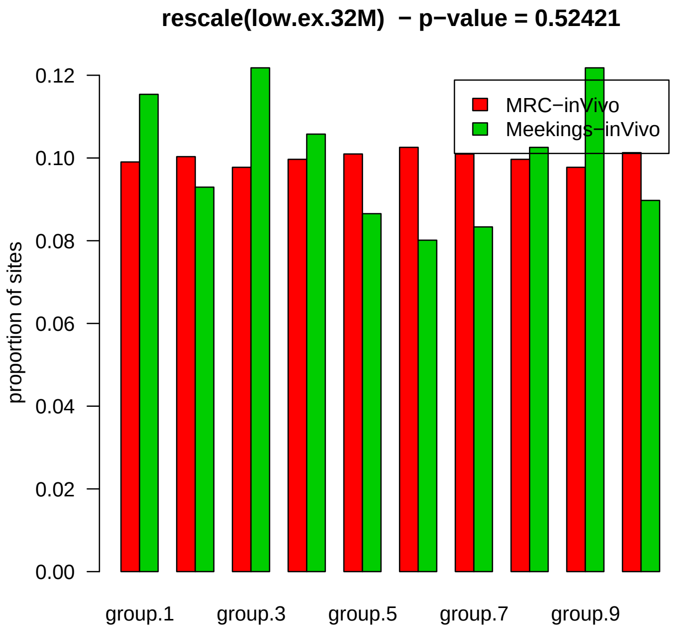

Now we count genes in the upper $1/8^{th}$:

```
Category limits

        lower category      upper
1  -0.9997078  group.1 -0.8000292
2  -0.8000292  group.2 -0.6000584
3  -0.6000584  group.3 -0.3998831
4  -0.3998831  group.4 -0.1998831
5  -0.1998831  group.5  0.0000000
6   0.0000000  group.6  0.1999416
7   0.1999416  group.7  0.4011105
8   0.4011105  group.8  0.5998247
9   0.5998247  group.9  0.7997662
10  0.7997662 group.10  0.9997078
```

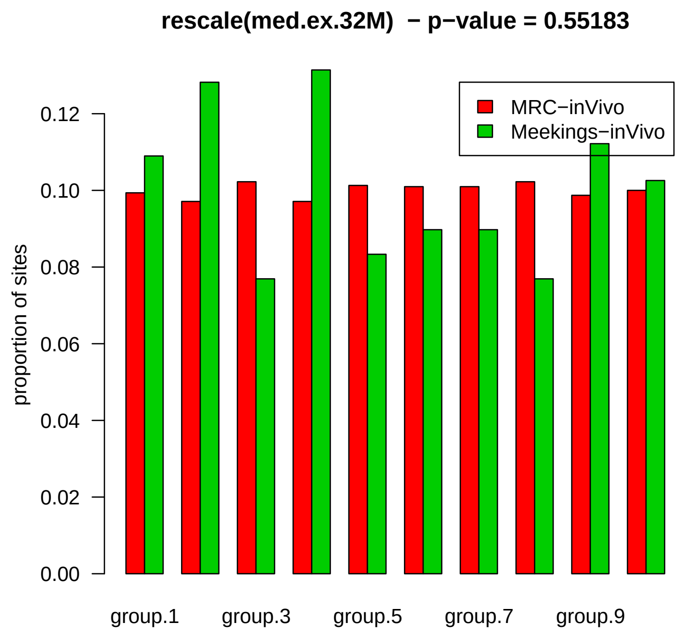

And here we count genes in the upper $1/16^{th}$:

```
Category limits

          lower  category         upper
1  -0.9997077732  group.1 -0.8012857978
2  -0.8012857978  group.2 -0.6002922268
3  -0.6002922268  group.3 -0.3997954413
4  -0.3997954413  group.4 -0.1996493279
5  -0.1996493279  group.5  0.0002922268
6   0.0002922268  group.6  0.2004675628
7   0.2004675628  group.7  0.3998831093
8   0.3998831093  group.8  0.5998246639
9   0.5998246639  group.9  0.7997662186
10  0.7997662186 group.10  0.9997077732
```

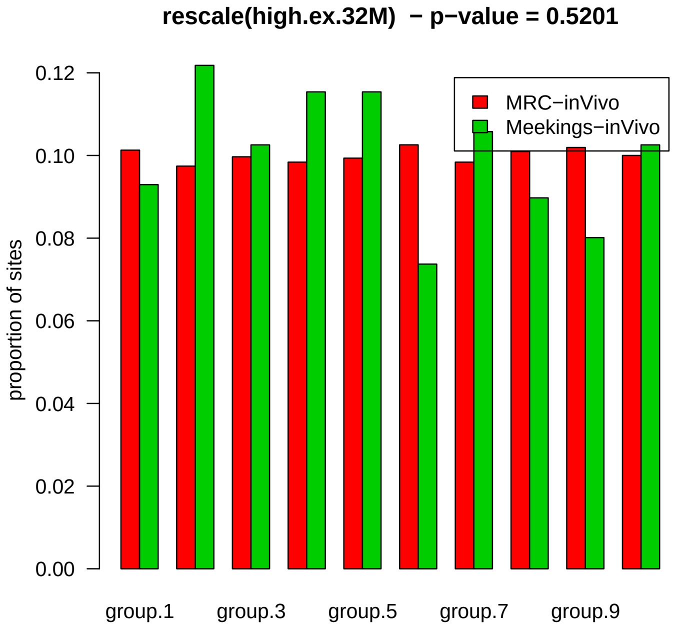

Here the effect of density of CpG islands is studied:

```
Category limits

        lower category      upper
1  -0.9997078  group.1 -0.8013442
2  -0.8013442  group.2 -0.5987726
3  -0.5987726  group.3 -0.3998831
4  -0.3998831  group.4 -0.1999416
5  -0.1999416  group.5  0.0000000
6   0.0000000  group.6  0.1999416
7   0.1999416  group.7  0.3998831
8   0.3998831  group.8  0.5998247
9   0.5998247  group.9  0.7997662
10  0.7997662 group.10  0.9997078
```

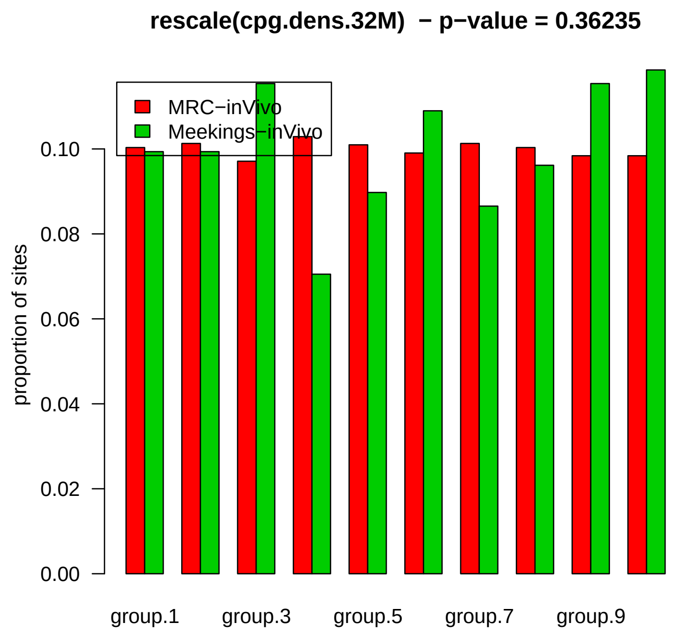

5 Juxtaposition with Gene Start and End Positions

5.1 Acembly Annotations

In this section we study the effect of juxtaposition in terms of gene start and end positions. The first barplot shows the effect of gene width for those insertions that are located within an Acembly gene.

```
 Category limits

      lower category     upper
1     362.0  group.1   14914.4
2   14914.4  group.2   29293.0
3   29293.0  group.3   45850.6
4   45850.6  group.4   67381.6
5   67381.6  group.5   96615.0
6   96615.0  group.6  135059.4
7  135059.4  group.7  192917.6
8  192917.6  group.8  276257.4
9  276257.4  group.9  437224.0
10 437224.0 group.10 1773723.0
```

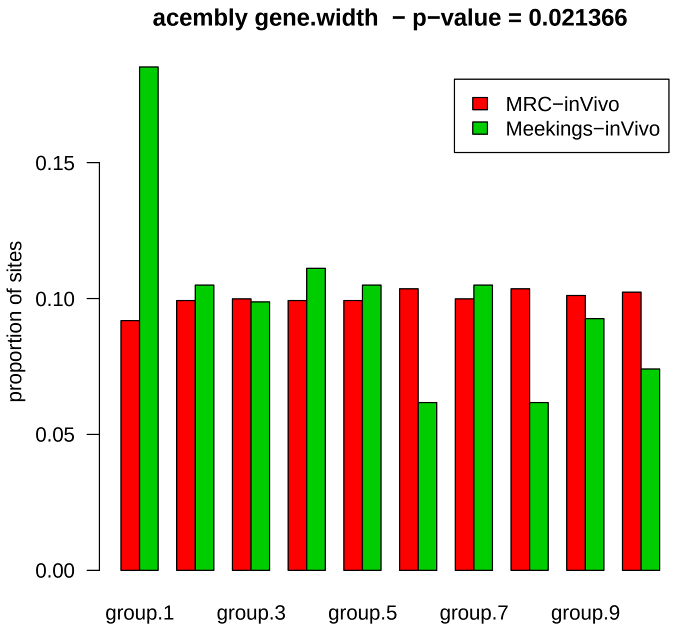

The next plot uses the width of a non-gene region for insertions that fall into such regions.

```
 Category limits

      lower category     upper
1     120.0  group.1   18615.4
2   18615.4  group.2   34325.6
3   34325.6  group.3   54458.8
4   54458.8  group.4   80337.2
5   80337.2  group.5  111311.0
6  111311.0  group.6  150976.0
7  150976.0  group.7  207108.6
8  207108.6  group.8  269094.0
9  269094.0  group.9  400207.2
10 400207.2 group.10 4780755.0
```

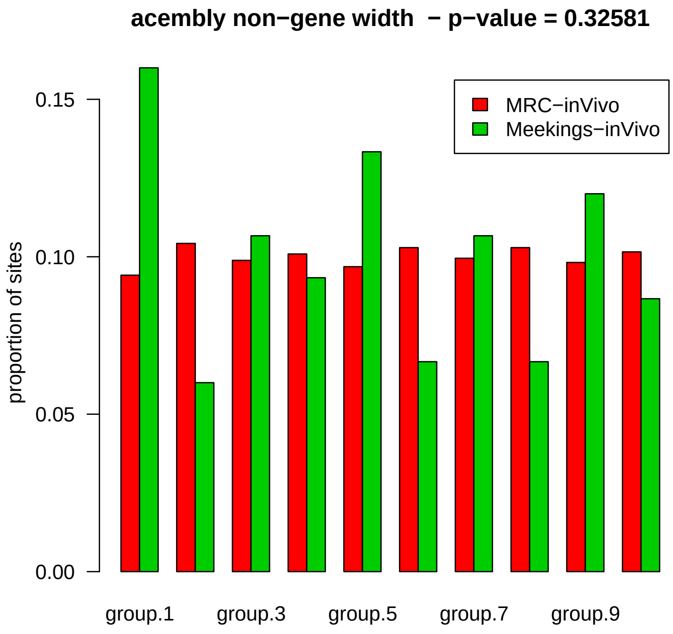

The next plot studies the distance to the nearest boundary between a gene and a non-gene region. The distance is expressed as a fraction of the length of the region. Thus, '0.25' refers to one quarter of the distance from the site to nearest boundary divided by the total width of the region.

```
 Category limits

          lower category      upper
1  0.0001599620  group.1 0.05065485
2  0.0506548472  group.2 0.09966634
3  0.0996663360  group.3 0.15153387
4  0.1515338748  group.4 0.20717075
5  0.2071707490  group.5 0.25591354
6  0.2559135379  group.6 0.30305987
7  0.3030598670  group.7 0.35197197
8  0.3519719675  group.8 0.40495750
9  0.4049575012  group.9 0.44935206
10 0.4493520561 group.10 0.49995258
```

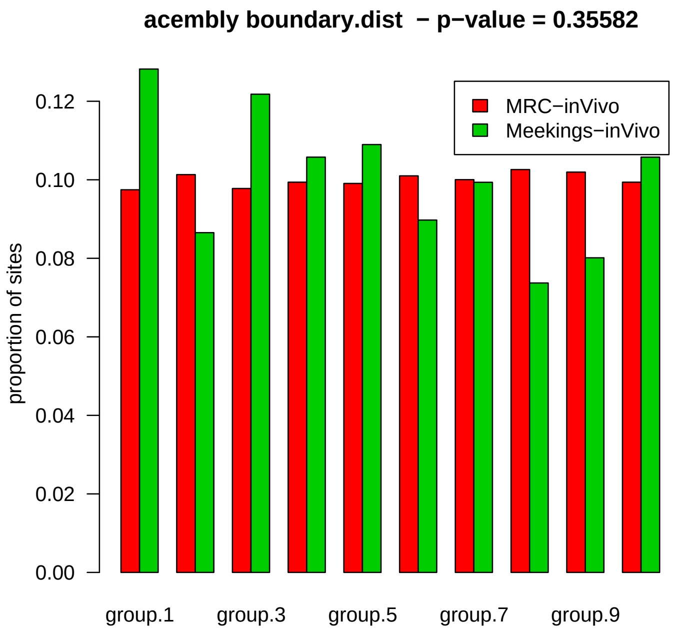

This plot studies the effect of nearness to the beginning of a transcript. For sites in genes, it is the distance to the start of the gene divided by the width of the gene. For other sites it is the distance from the site to the nearer gene if that gene boundary is also a transcription starting point. Locations near '0' are relatively near the beginning of transcription, while those near '1' are near the termination of the transcript.

```
 Category limits

          lower category      upper
1  0.0001599620  group.1 0.08509283
2  0.0850928327  group.2 0.17859766
3  0.1785976559  group.3 0.26783265
4  0.2678326511  group.4 0.35353832
5  0.3535383217  group.5 0.44178535
6  0.4417853523  group.6 0.53280485
7  0.5328048532  group.7 0.65234180
8  0.6523418049  group.8 0.76360265
9  0.7636026508  group.9 0.88528320
10 0.8852831985 group.10 0.99982891
```

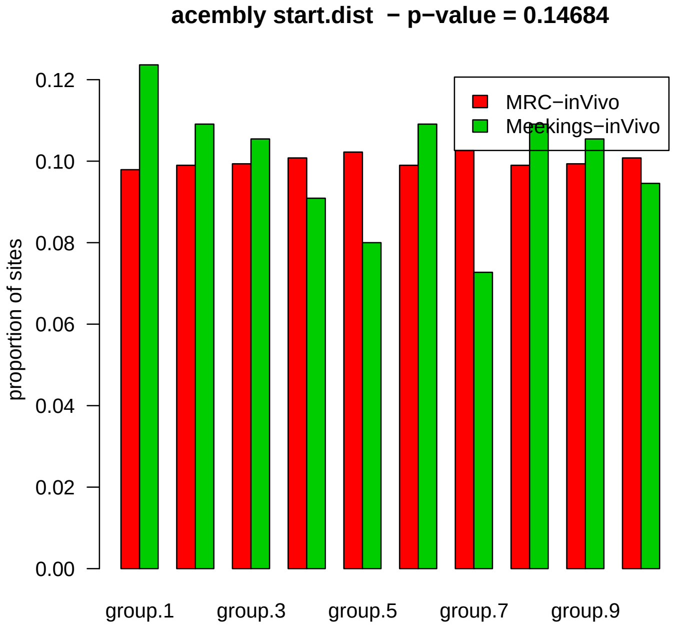

5.2 RefSeq Annotations

Category limits

	lower	category	upper
1	534.0	group.1	24949.6
2	24949.6	group.2	47982.4
3	47982.4	group.3	71734.4
4	71734.4	group.4	101679.6
5	101679.6	group.5	138009.0
6	138009.0	group.6	193518.6
7	193518.6	group.7	285423.8
8	285423.8	group.8	409350.4
9	409350.4	group.9	666493.0
10	666493.0	group.10	2304258.0

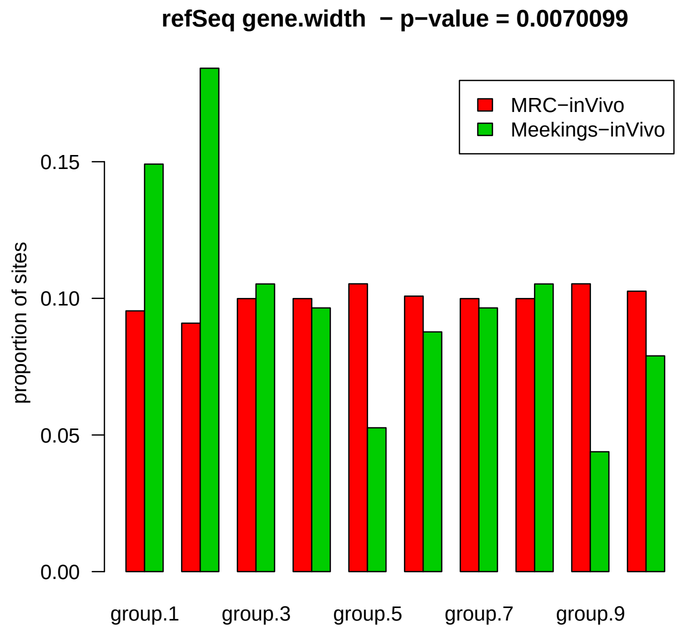

Category limits

	lower	category	upper
1	342.0	group.1	46756.7
2	46756.7	group.2	109550.0
3	109550.0	group.3	202096.0
4	202096.0	group.4	337935.0

5	337935.0	group.5	495886.0
6	495886.0	group.6	761648.0
7	761648.0	group.7	1142264.7
8	1142264.7	group.8	1660984.0
9	1660984.0	group.9	2630125.0
10	2630125.0	group.10	21293005.0

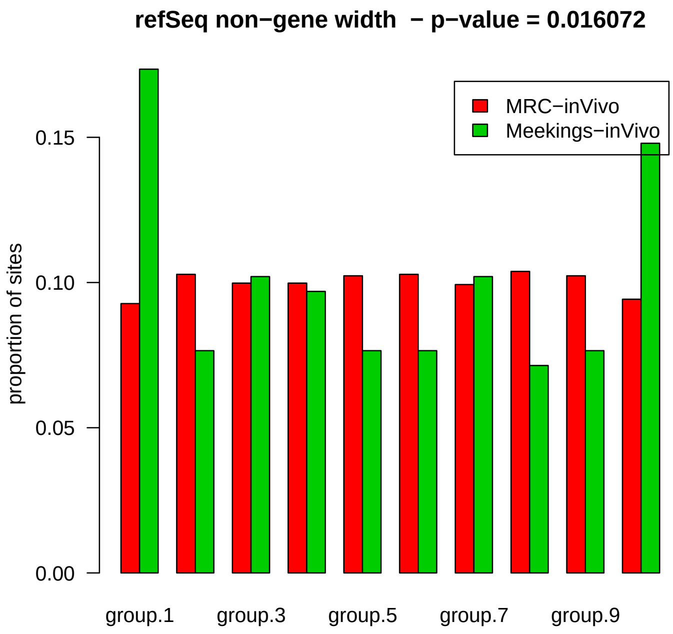

Category limits

	lower	category	upper
1	3.758743e-05	group.1	0.04931092
2	4.931092e-02	group.2	0.10066226
3	1.006623e-01	group.3	0.14852379
4	1.485238e-01	group.4	0.20063881
5	2.006388e-01	group.5	0.24959863
6	2.495986e-01	group.6	0.29747416
7	2.974742e-01	group.7	0.34532485
8	3.453248e-01	group.8	0.39764028
9	3.976403e-01	group.9	0.44942186
10	4.494219e-01	group.10	0.49978476

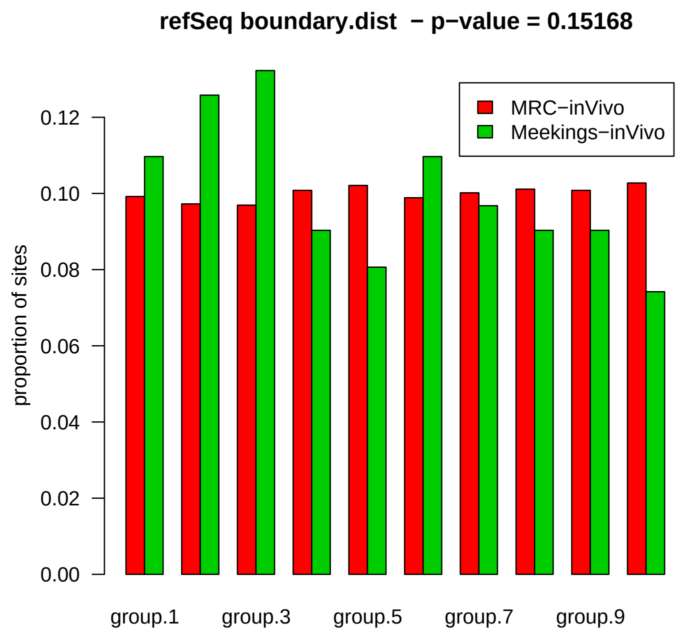

Category limits

	lower	category	upper
1	7.536931e-05	group.1	0.09052848
2	9.052848e-02	group.2	0.17487493
3	1.748749e-01	group.3	0.27143775
4	2.714377e-01	group.4	0.35091628
5	3.509163e-01	group.5	0.44012697
6	4.401270e-01	group.6	0.53356580
7	5.335658e-01	group.7	0.65260764
8	6.526076e-01	group.8	0.76085987
9	7.608599e-01	group.9	0.88028788
10	8.802879e-01	group.10	0.99967333

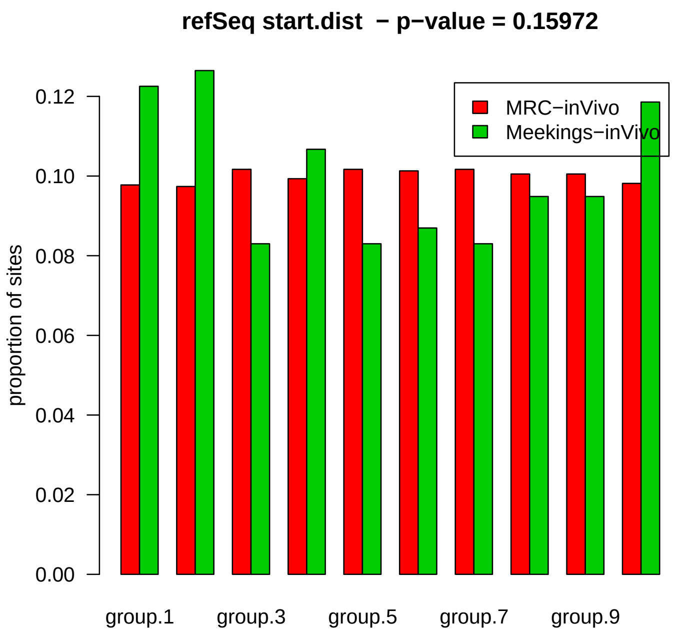
refSeq start.dist − p−value = 0.15972
MRC−inVivo
Meekings−inVivo
proportion of sites
0.00
0.02
0.04
0.06
0.08
0.10
0.12
group.1
group.3
group.5
group.7
group.9

5.3 genScan Annotations

Category limits

	lower	category	upper
1	819.0	group.1	26121.5
2	26121.5	group.2	43886.0
3	43886.0	group.3	59298.5
4	59298.5	group.4	77203.0
5	77203.0	group.5	99598.0
6	99598.0	group.6	122996.0
7	122996.0	group.7	156757.0
8	156757.0	group.8	206318.0
9	206318.0	group.9	285581.5
10	285581.5	group.10	1232888.0

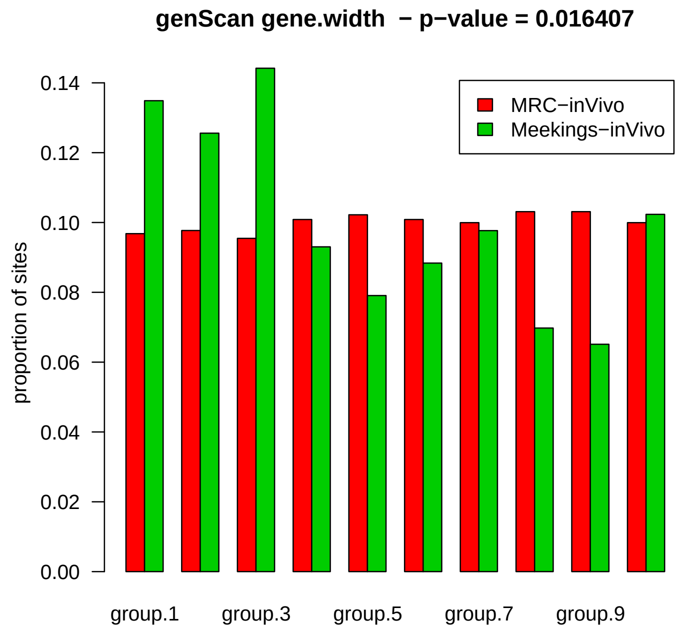

Category limits

	lower	category	upper
1	971.0	group.1	8898.8
2	8898.8	group.2	14437.0
3	14437.0	group.3	19813.7
4	19813.7	group.4	27396.4

5	27396.4	group.5	34843.0
6	34843.0	group.6	42776.2
7	42776.2	group.7	55423.9
8	55423.9	group.8	78447.4
9	78447.4	group.9	140444.5
10	140444.5	group.10	4642660.0

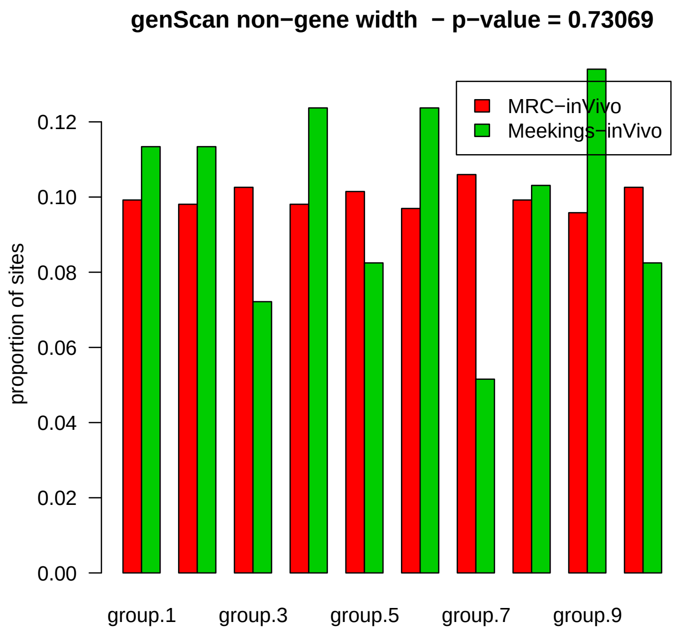

Category limits

	lower	category	upper
1	8.927774e-05	group.1	0.04877858
2	4.877858e-02	group.2	0.09413327
3	9.413327e-02	group.3	0.14633880
4	1.463388e-01	group.4	0.20036384
5	2.003638e-01	group.5	0.25211606
6	2.521161e-01	group.6	0.30236608
7	3.023661e-01	group.7	0.35404268
8	3.540427e-01	group.8	0.40136239
9	4.013624e-01	group.9	0.44856371
10	4.485637e-01	group.10	0.49979502

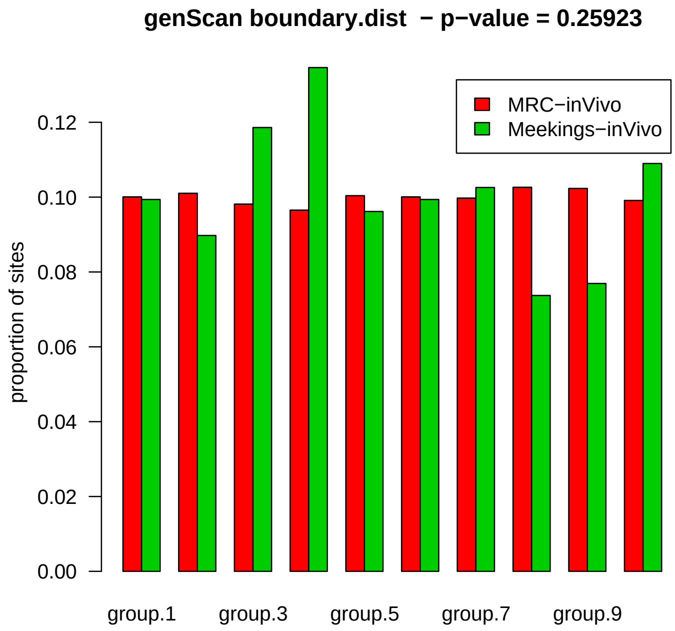

Category limits

	lower	category	upper
1	0.0001007711	group.1	0.08329964
2	0.0832996419	group.2	0.18644323
3	0.1864432298	group.3	0.28778984
4	0.2877898382	group.4	0.37790327
5	0.3779032695	group.5	0.47018927
6	0.4701892733	group.6	0.57020018
7	0.5702001837	group.7	0.67418080
8	0.6741807976	group.8	0.78785037
9	0.7878503693	group.9	0.89779058
10	0.8977905804	group.10	0.99991072

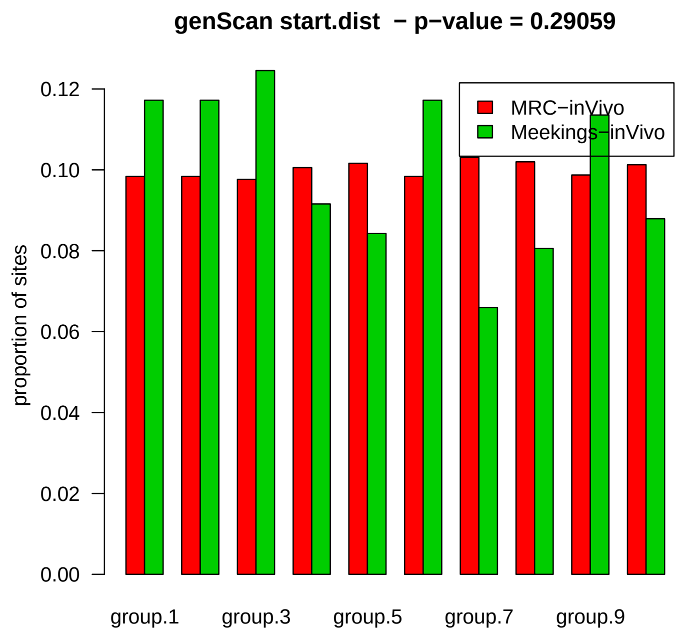
genScan start.dist − p−value = 0.29059
MRC−inVivo
Meekings−inVivo
proportion of sites
0.00
0.02
0.04
0.06
0.08
0.10
0.12
group.1
group.3
group.5
group.7
group.9

5.4 uniGene Annotations

Category limits

	lower	category	upper
1	819.0	group.1	26121.5
2	26121.5	group.2	43886.0
3	43886.0	group.3	59298.5
4	59298.5	group.4	77203.0
5	77203.0	group.5	99598.0
6	99598.0	group.6	122996.0
7	122996.0	group.7	156757.0
8	156757.0	group.8	206318.0
9	206318.0	group.9	285581.5
10	285581.5	group.10	1232888.0

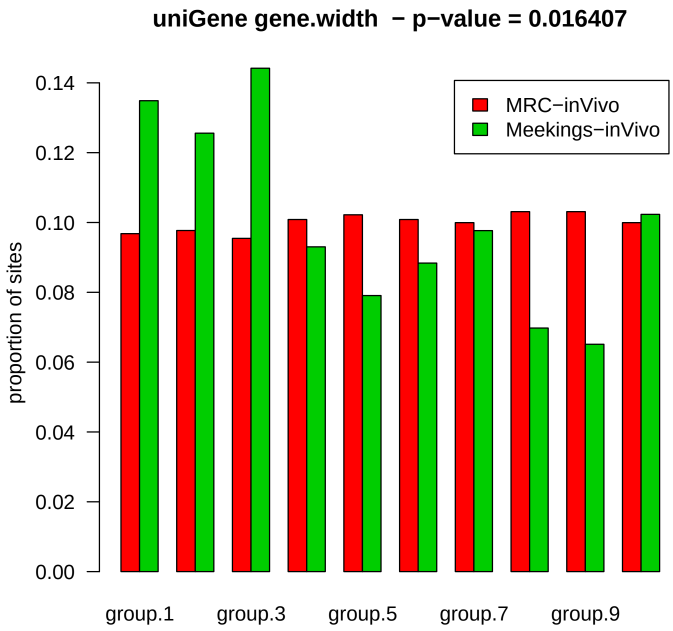

Category limits

	lower	category	upper
1	971.0	group.1	8898.8
2	8898.8	group.2	14437.0
3	14437.0	group.3	19813.7
4	19813.7	group.4	27396.4

5	27396.4	group.5	34843.0
6	34843.0	group.6	42776.2
7	42776.2	group.7	55423.9
8	55423.9	group.8	78447.4
9	78447.4	group.9	140444.5
10	140444.5	group.10	4642660.0

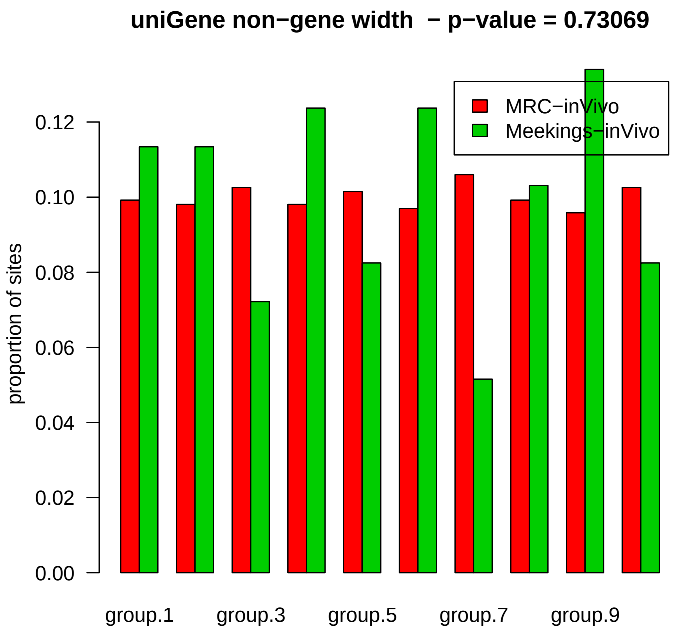

Category limits

	lower	category	upper
1	8.927774e-05	group.1	0.04877858
2	4.877858e-02	group.2	0.09413327
3	9.413327e-02	group.3	0.14633880
4	1.463388e-01	group.4	0.20036384
5	2.003638e-01	group.5	0.25211606
6	2.521161e-01	group.6	0.30236608
7	3.023661e-01	group.7	0.35404268
8	3.540427e-01	group.8	0.40136239
9	4.013624e-01	group.9	0.44856371
10	4.485637e-01	group.10	0.49979502

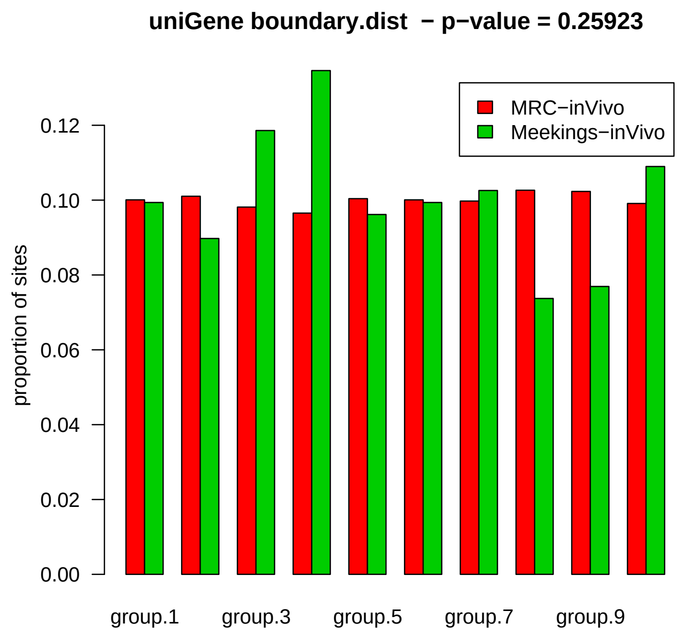

Category limits

	lower	category	upper
1	0.0001007711	group.1	0.08329964
2	0.0832996419	group.2	0.18644323
3	0.1864432298	group.3	0.28778984
4	0.2877898382	group.4	0.37790327
5	0.3779032695	group.5	0.47018927
6	0.4701892733	group.6	0.57020018
7	0.5702001837	group.7	0.67418080
8	0.6741807976	group.8	0.78785037
9	0.7878503693	group.9	0.89779058
10	0.8977905804	group.10	0.99991072

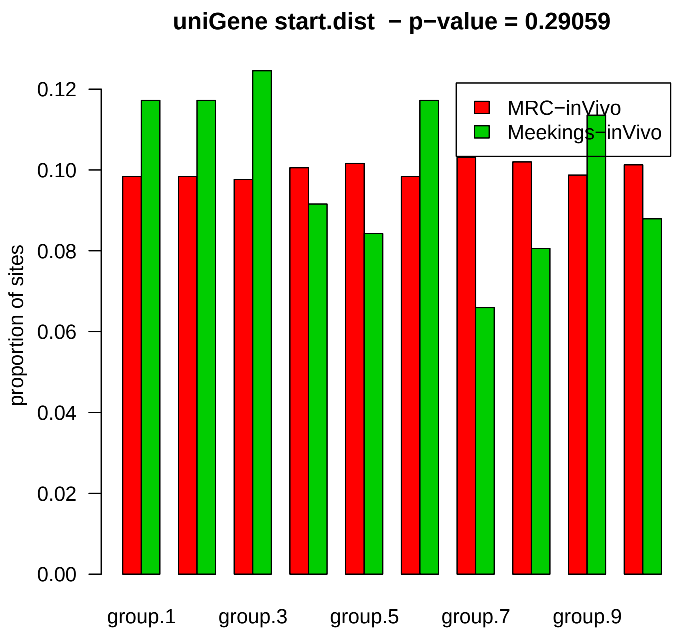
uniGene start.dist − p−value = 0.29059
proportion of sites
0.00
0.02
0.04
0.06
0.08
0.10
0.12
MRC−inVivo
Meekings−inVivo
group.1
group.3
group.5
group.7
group.9

6 GC content

Here we study the effect of GC content on insertion. The GC content is taken from the Mouse Genome Draft at GoldenPath from the table

Following the plot is a table of fitted coefficients based on splitting the GC percent data at the median.

```
 Category limits

  lower category upper
1  0.00  group.1  0.25
2  0.25  group.2  0.30
3  0.30  group.3  0.35
4  0.35  group.4  0.40
5  0.40  group.5  0.45
6  0.45  group.6  0.50
7  0.50  group.7  0.55
8  0.55  group.8  0.60
9  0.60  group.9  0.85
```

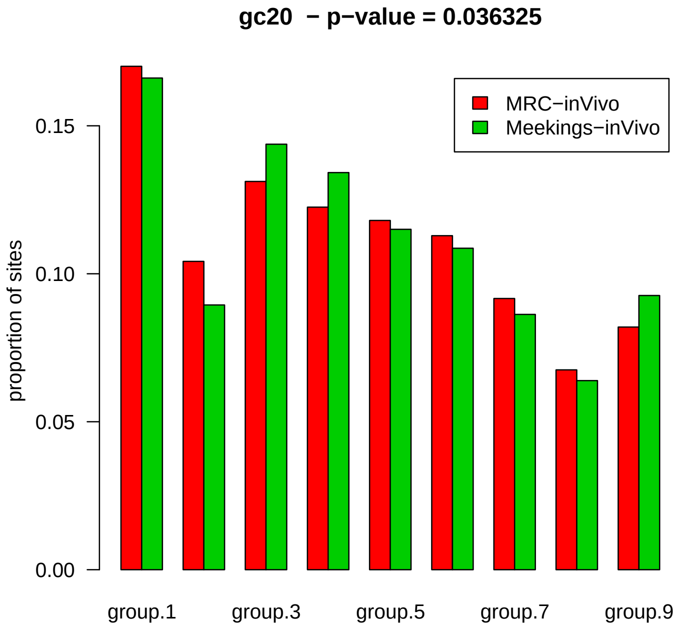

	coef	se	z	p
(Intercept)	-2.510	0.0858	-29.20	9.33e-188
eval(the.gene)	0.443	0.1190	3.73	1.94e-04

Category limits

	lower	category	upper
1	0.04	group.1	0.26
2	0.26	group.2	0.32
3	0.32	group.3	0.34
4	0.34	group.4	0.38
5	0.38	group.5	0.42
6	0.42	group.6	0.44
7	0.44	group.7	0.48
8	0.48	group.8	0.52
9	0.52	group.9	0.58
10	0.58	group.10	0.84

	coef	se	z	p
(Intercept)	-2.510	0.0858	-29.20	9.33e-188
eval(the.gene)	0.443	0.1190	3.73	1.94e-04

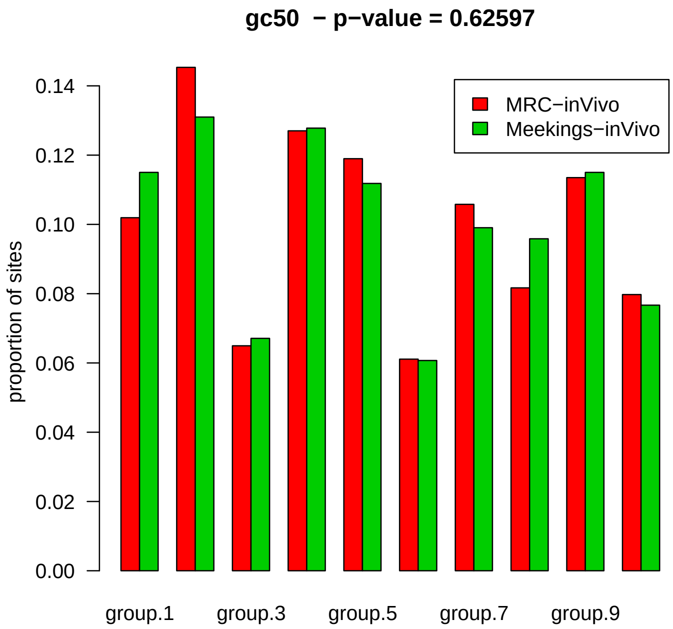

```
 Category limits

   lower category upper
1   0.03  group.1  0.29
2   0.29  group.2  0.32
3   0.32  group.3  0.35
4   0.35  group.4  0.38
5   0.38  group.5  0.41
6   0.41  group.6  0.44
7   0.44  group.7  0.47
8   0.47  group.8  0.51
9   0.51  group.9  0.56
10  0.56 group.10  0.87

                  coef     se      z         p
(Intercept)     -2.510 0.0858 -29.20 9.33e-188
eval(the.gene)   0.443 0.1190   3.73  1.94e-04
```

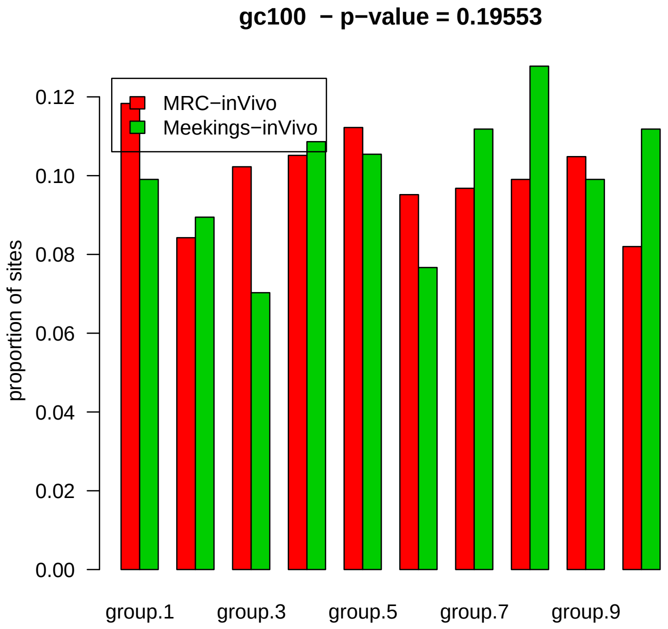

Category limits

	lower	category	upper
1	0.056	group.1	0.308
2	0.308	group.2	0.340
3	0.340	group.3	0.364
4	0.364	group.4	0.388
5	0.388	group.5	0.408
6	0.408	group.6	0.436
7	0.436	group.7	0.460
8	0.460	group.8	0.492
9	0.492	group.9	0.528
10	0.528	group.10	0.820

	coef	se	z	p
(Intercept)	-2.510	0.0858	-29.20	9.33e-188
eval(the.gene)	0.443	0.1190	3.73	1.94e-04

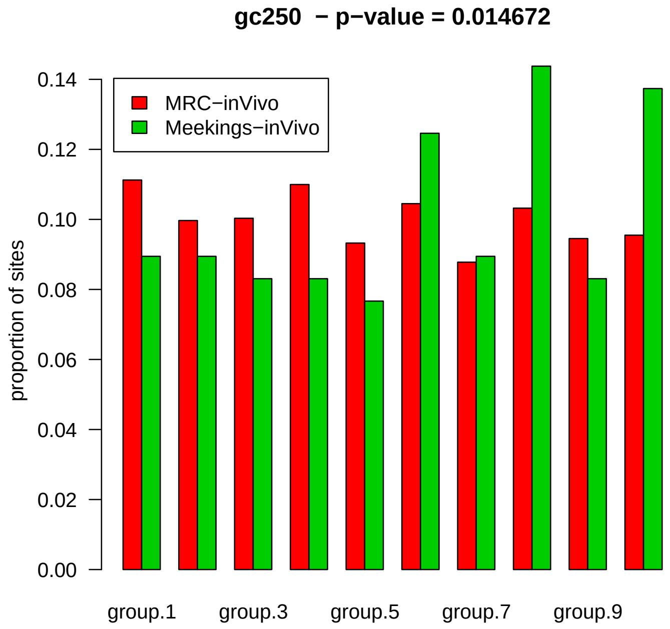

Category limits

	lower	category	upper
1	0.210	group.1	0.316
2	0.316	group.2	0.348
3	0.348	group.3	0.370
4	0.370	group.4	0.390
5	0.390	group.5	0.408
6	0.408	group.6	0.430
7	0.430	group.7	0.452
8	0.452	group.8	0.478
9	0.478	group.9	0.516
10	0.516	group.10	0.818

	coef	se	z	p
(Intercept)	-2.510	0.0858	-29.20	9.33e-188
eval(the.gene)	0.443	0.1190	3.73	1.94e-04

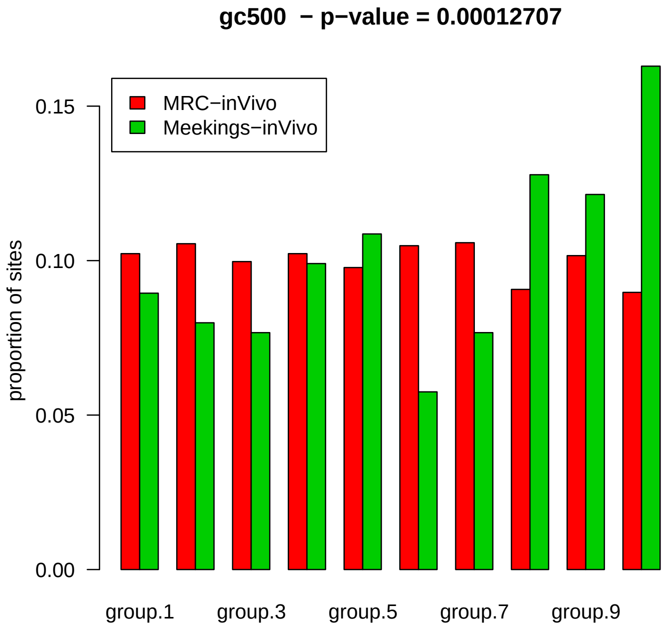

Category limits

	lower	category	upper
1	0.228	group.1	0.330
2	0.330	group.2	0.353
3	0.353	group.3	0.373
4	0.373	group.4	0.389
5	0.389	group.5	0.406
6	0.406	group.6	0.423
7	0.423	group.7	0.443
8	0.443	group.8	0.469
9	0.469	group.9	0.506
10	0.506	group.10	0.751

	coef	se	z	p
(Intercept)	-2.510	0.0858	-29.20	9.33e-188
eval(the.gene)	0.443	0.1190	3.73	1.94e-04

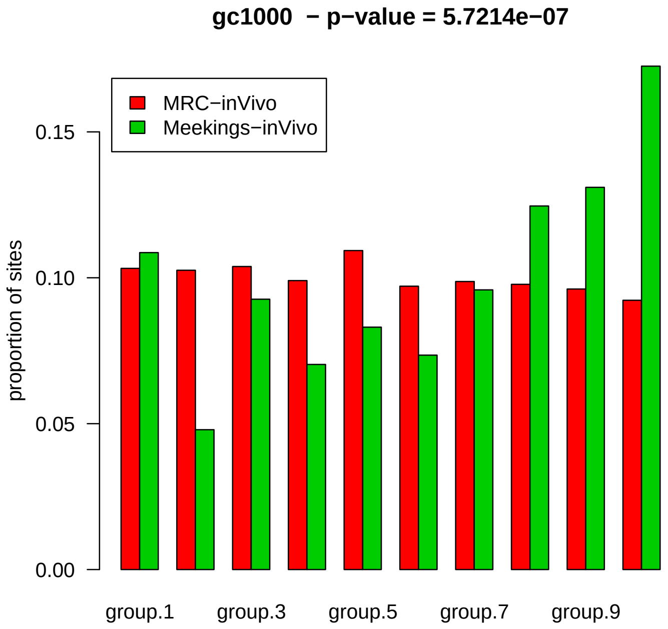

Category limits

	lower	category	upper
1	0.2460	group.1	0.3370
2	0.3370	group.2	0.3580
3	0.3580	group.3	0.3730
4	0.3730	group.4	0.3865
5	0.3865	group.5	0.4015
6	0.4015	group.6	0.4195
7	0.4195	group.7	0.4415
8	0.4415	group.8	0.4645
9	0.4645	group.9	0.5019
10	0.5019	group.10	0.7275

	coef	se	z	p
(Intercept)	-2.510	0.0858	-29.20	9.33e-188
eval(the.gene)	0.443	0.1190	3.73	1.94e-04

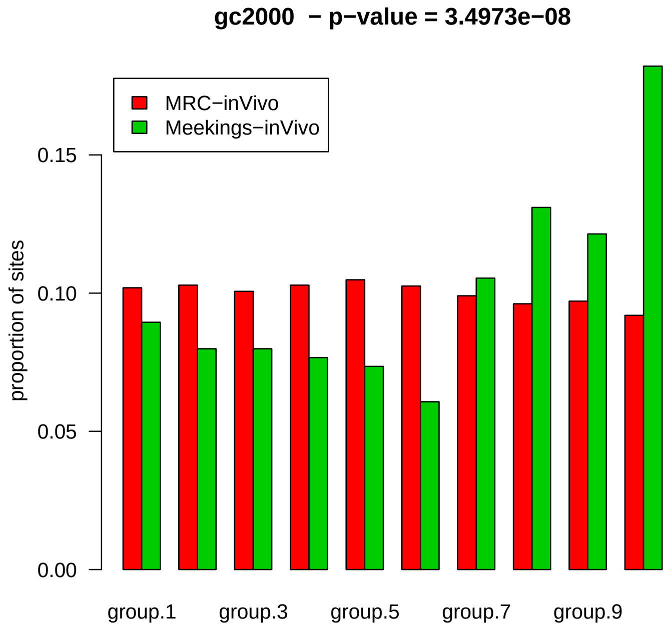

Category limits

	lower	category	upper
1	0.27340	group.1	0.34464
2	0.34464	group.2	0.36260
3	0.36260	group.3	0.37572
4	0.37572	group.4	0.38840
5	0.38840	group.5	0.40160
6	0.40160	group.6	0.41640
7	0.41640	group.7	0.43588
8	0.43588	group.8	0.45920
9	0.45920	group.9	0.49796
10	0.49796	group.10	0.66960

	coef	se	z	p
(Intercept)	-2.510	0.0858	-29.20	9.33e-188
eval(the.gene)	0.443	0.1190	3.73	1.94e-04

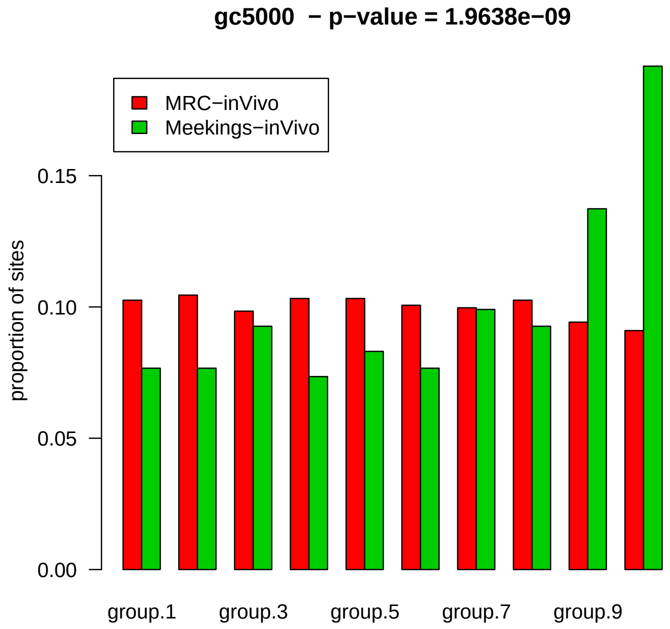

Category limits

	lower	category	upper
1	0.29850	group.1	0.34920
2	0.34920	group.2	0.36520
3	0.36520	group.3	0.37676
4	0.37676	group.4	0.38880
5	0.38880	group.5	0.40100
6	0.40100	group.6	0.41572
7	0.41572	group.7	0.43270
8	0.43270	group.8	0.45632
9	0.45632	group.9	0.49310
10	0.49310	group.10	0.64880

	coef	se	z	p
(Intercept)	-2.510	0.0858	-29.20	9.33e-188
eval(the.gene)	0.443	0.1190	3.73	1.94e-04

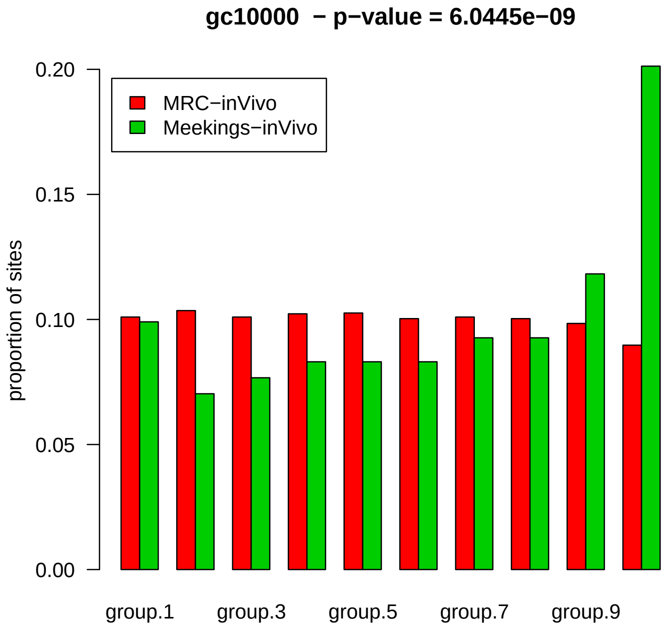

Category limits

	lower	category	upper
1	0.316240	group.1	0.353720
2	0.353720	group.2	0.366560
3	0.366560	group.3	0.377064
4	0.377064	group.4	0.388320
5	0.388320	group.5	0.400320
6	0.400320	group.6	0.414408
7	0.414408	group.7	0.430816
8	0.430816	group.8	0.454448
9	0.454448	group.9	0.492464
10	0.492464	group.10	0.650960

	coef	se	z	p
(Intercept)	-2.510	0.0858	-29.20	9.33e-188
eval(the.gene)	0.443	0.1190	3.73	1.94e-04

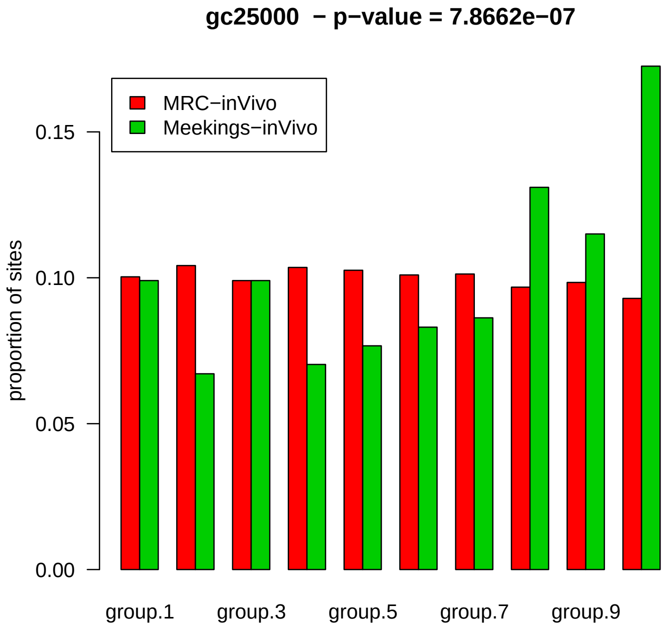

Category limits

	lower	category	upper
1	0.321620	group.1	0.355196
2	0.355196	group.2	0.367404
3	0.367404	group.3	0.377936
4	0.377936	group.4	0.388740
5	0.388740	group.5	0.401840
6	0.401840	group.6	0.414488
7	0.414488	group.7	0.431584
8	0.431584	group.8	0.452096
9	0.452096	group.9	0.489072
10	0.489072	group.10	0.632940

	coef	se	z	p
(Intercept)	-2.510	0.0858	-29.20	9.33e-188
eval(the.gene)	0.443	0.1190	3.73	1.94e-04

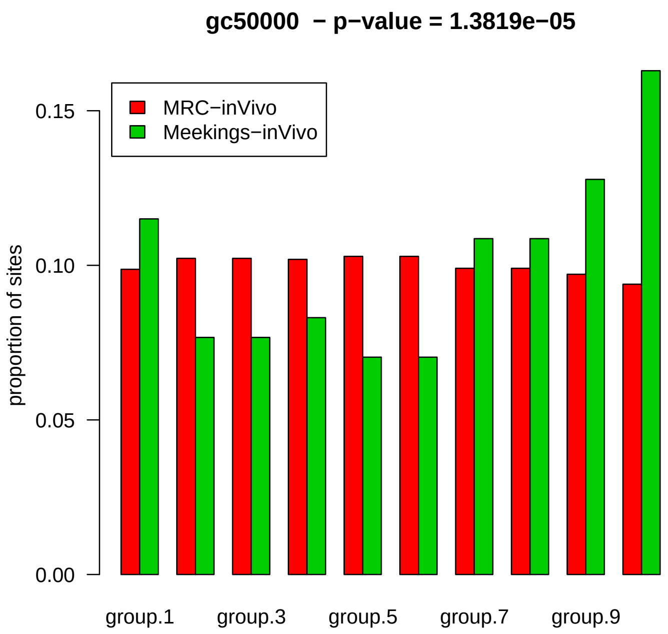

Category limits

	lower	category	upper
1	0.326490	group.1	0.356060
2	0.356060	group.2	0.368334
3	0.368334	group.3	0.379076
4	0.379076	group.4	0.388854
5	0.388854	group.5	0.402000
6	0.402000	group.6	0.415332
7	0.415332	group.7	0.430426
8	0.430426	group.8	0.450908
9	0.450908	group.9	0.485204
10	0.485204	group.10	0.623150

	coef	se	z	p
(Intercept)	-2.510	0.0858	-29.20	9.33e-188
eval(the.gene)	0.443	0.1190	3.73	1.94e-04

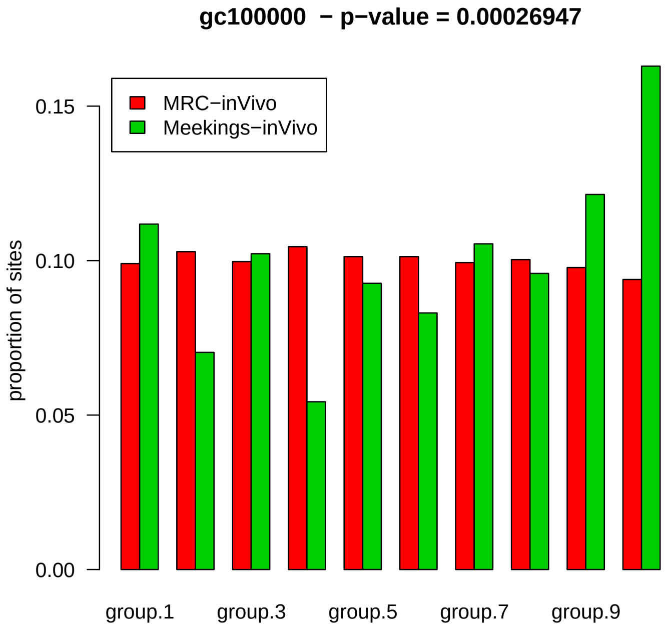

Category limits

	lower	category	upper
1	0.3327160	group.1	0.3567352
2	0.3567352	group.2	0.3693504
3	0.3693504	group.3	0.3805856
4	0.3805856	group.4	0.3906432
5	0.3906432	group.5	0.4030120
6	0.4030120	group.6	0.4152456
7	0.4152456	group.7	0.4296512
8	0.4296512	group.8	0.4500852
9	0.4500852	group.9	0.4811648
10	0.4811648	group.10	0.6238080

	coef	se	z	p
(Intercept)	-2.510	0.0858	-29.20	9.33e-188
eval(the.gene)	0.443	0.1190	3.73	1.94e-04

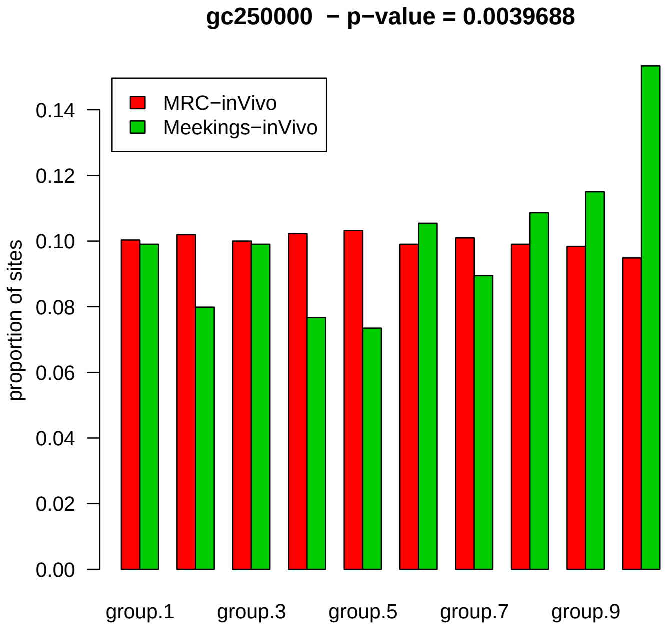

Category limits

	lower	category	upper
1	0.3360480	group.1	0.3569236
2	0.3569236	group.2	0.3706316
3	0.3706316	group.3	0.3817764
4	0.3817764	group.4	0.3916436
5	0.3916436	group.5	0.4040080
6	0.4040080	group.6	0.4155932
7	0.4155932	group.7	0.4298860
8	0.4298860	group.8	0.4494324
9	0.4494324	group.9	0.4776624
10	0.4776624	group.10	0.6081040

	coef	se	z	p
(Intercept)	-2.510	0.0858	-29.20	9.33e-188
eval(the.gene)	0.443	0.1190	3.73	1.94e-04

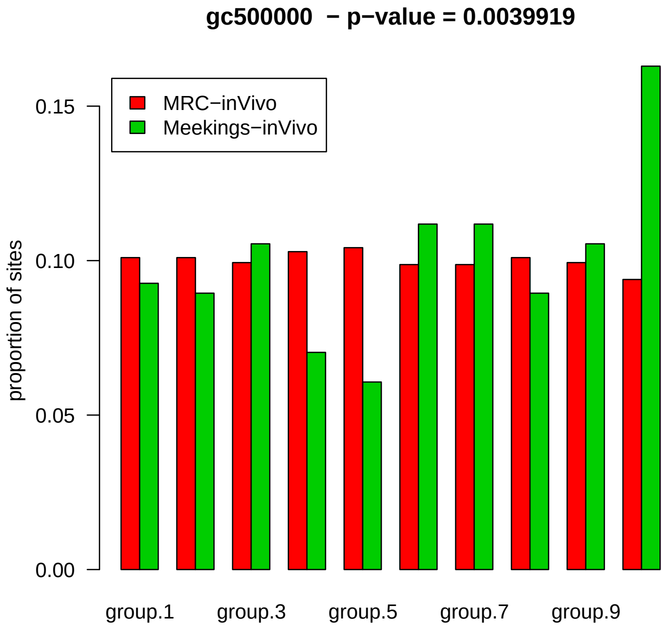

Category limits

	lower	category	upper
1	0.3373960	group.1	0.3583892
2	0.3583892	group.2	0.3712814
3	0.3712814	group.3	0.3826826
4	0.3826826	group.4	0.3934876
5	0.3934876	group.5	0.4053140
6	0.4053140	group.6	0.4158462
7	0.4158462	group.7	0.4292004
8	0.4292004	group.8	0.4478976
9	0.4478976	group.9	0.4740026
10	0.4740026	group.10	0.5973310

	coef	se	z	p
(Intercept)	-2.510	0.0858	-29.20	9.33e-188
eval(the.gene)	0.443	0.1190	3.73	1.94e-04

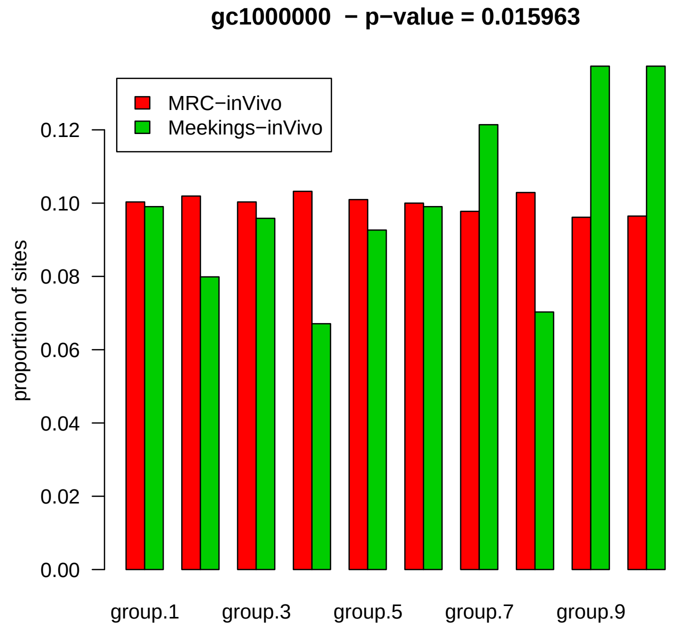

Category limits

	lower	category	upper
1	0.3431200	group.1	0.3655166
2	0.3655166	group.2	0.3761426
3	0.3761426	group.3	0.3867794
4	0.3867794	group.4	0.3959494
5	0.3959494	group.5	0.4050850
6	0.4050850	group.6	0.4157468
7	0.4157468	group.7	0.4275466
8	0.4275466	group.8	0.4428030
9	0.4428030	group.9	0.4676686
10	0.4676686	group.10	0.5723870

	coef	se	z	p
(Intercept)	-2.510	0.0858	-29.20	9.33e-188
eval(the.gene)	0.443	0.1190	3.73	1.94e-04

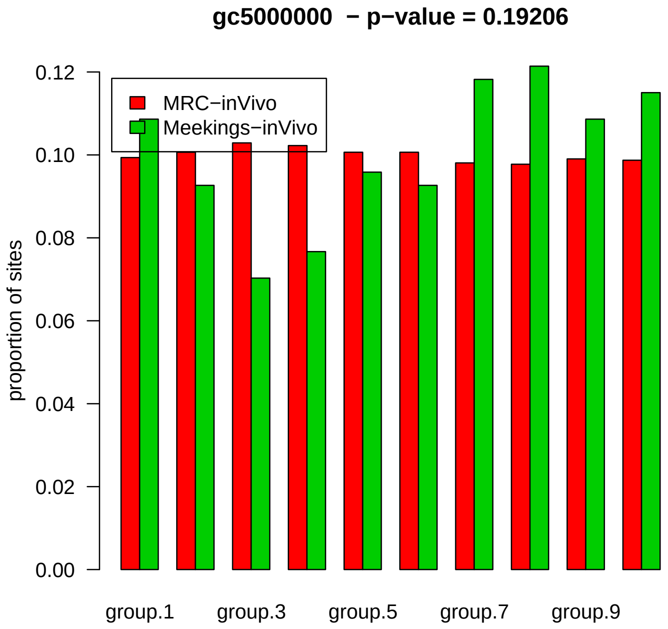

Category limits

	lower	category	upper
1	0.3465080	group.1	0.3689412
2	0.3689412	group.2	0.3792824
3	0.3792824	group.3	0.3888328
4	0.3888328	group.4	0.3972814
5	0.3972814	group.5	0.4061530
6	0.4061530	group.6	0.4149304
7	0.4149304	group.7	0.4239636
8	0.4239636	group.8	0.4396482
9	0.4396482	group.9	0.4606730
10	0.4606730	group.10	0.5545020

	coef	se	z	p
(Intercept)	-2.510	0.0858	-29.20	9.33e-188
eval(the.gene)	0.443	0.1190	3.73	1.94e-04

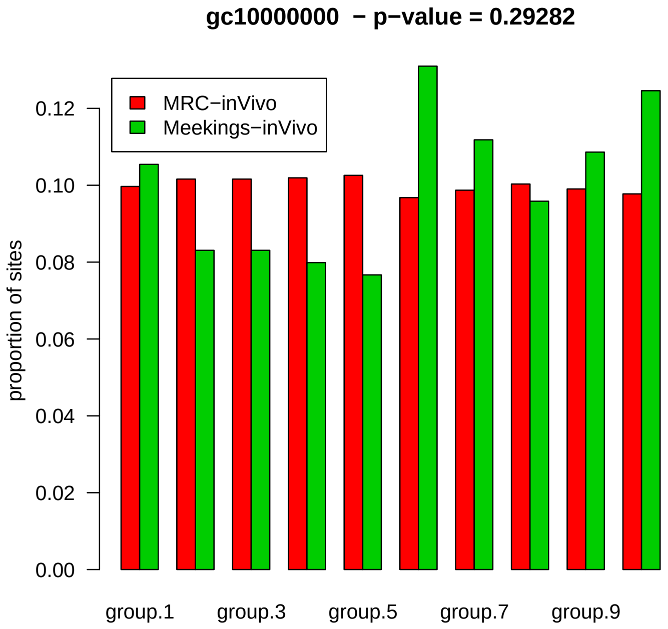

7 Cytobands

Here we study the association of cytoband with insertion intensity. The data are obtained from
`http://genome.ucsc.edu/goldenPath/hg17/database/cytoBand.txt.gz`.

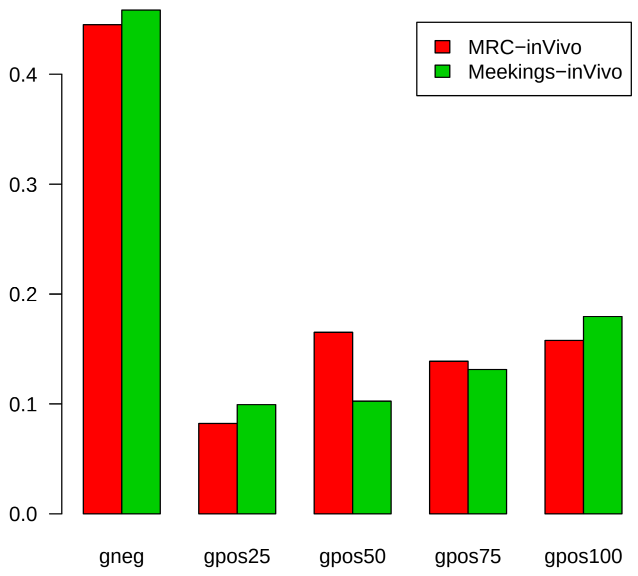

A formal test of significance attains a p-value of 0.044387.

References

[1] P. McCullagh and John A. Nelder. *Generalized linear models.* (Chapman & Hall ltd, 1999).

[2] Xiaolin Wu, Yuan Li, Bruce Crise, Shawn M. Burgess "Transcription Start Regions in the Human Genome Are Favored Targets for MLV Integration," *Science,* **300**(5626), (June 2003): 1749-1751.
