# Supplementary material for: HTLV-1 Integration into Transcriptionally Active Genomic Regions Is Associated with Proviral Expression and with HAM/TSP
Source: PLoS Pathog. 2008 Mar 21;4(3):e1000027. doi: 10.1371/journal.ppat.1000027 (PMC2265437; doi:10.1371/journal.ppat.1000027)
Supplement: Table S1 — Classification of individuals used in this study. Identification codes, Tax expression levels (% of CD4+ T cells expressing Tax), Proviral loads (number of provirus positive cells per 100 PBMCs), disease status and number of contributing sites are given. (0.05 MB DOC) [file ppat.1000027.s004.doc]

**Table S1: Classification of individuals used in this study**

| Patient | Tax^a^ | Load^b^ | Disease Status | Number Mapped Sites: |
| --- | --- | --- | --- | --- |
| HAY | 6.6 | 5.45 | Healthy | 24 |
| HBK | 2.2 | 8.04 | Healthy | 21 |
| HBM | 8 | 15.9 | Healthy | 12 |
| HBO | 0.16 | 3.07 | Healthy | 4 |
| HBQ | 1.6 | 2.2 | Healthy | 15 |
| HBZ | 1.5 | 6.94 | Healthy | 9 |
| HCB | 3.5 | 4.01 | Healthy | 12 |
| HCL | 0 | 0.51 | Healthy | 1 |
| HS | 1 | 10.3 | Healthy | 16 |
| HT | 1.78 | 7.8 | Healthy | 16 |
| HX | 0.03 | 0.49 | Healthy | 2 |
| TAN | 1.9 | 6.51 | HAM/TSP | 11 |
| TAQ | 0.9 | 7.75 | HAM/TSP | 10 |
| TAU | 9.66 | 6.77 | HAM/TSP | 7 |
| TAY | 1.5 | 4.9 | HAM/TSP | 20 |
| TAZ | 25.9 | 36.31 | HAM/TSP | 20 |
| TBA | 4.6 | 16.82 | HAM/TSP | 18 |
| TBG | 12.6 | 22.89 | HAM/TSP | 16 |
| TBK | 5.2 | 23.72 | HAM/TSP | 7 |
| TBO | 5.1 | 8.47 | HAM/TSP | 20 |
| TBR | 3 | 14.25 | HAM/TSP | 21 |
| TBU | 1.6 | 3.92 | HAM/TSP | 3 |
| TBW | 7.1 | 8.82 | HAM/TSP | 11 |
| TBZ | 4.5 | 13.11 | HAM/TSP | 17 |
| Total |  |  |  | 313 |

^a^ Percentage of CD4+ cells expressing Tax protein after 18h incubation *in vitro* of CD8+ cell-depleted PBMCs.

^b^ Proviral load, expressed as number of copies of HTLV-1 provirus/100 PBMCs.
